# On the Validation of Protein Force Fields Based on Structural Criteria

**DOI:** 10.1021/acs.jpcb.3c08469

**Published:** 2024-05-07

**Authors:** Martin Stroet, Martina Setz, Thomas Lee, Alpeshkumar K. Malde, Glen van den Bergen, Peter Sykacek, Chris Oostenbrink, Alan E. Mark

**Affiliations:** † The University of Queensland, St. Lucia, Queensland 4072, Australia; ‡ Institute for Molecular Modeling and Simulation, Department of Material Science and Process Engineering, University of Natural Resources and Life Sciences, Vienna Muthgasse 18, 1190 Vienna, Austria; § Institute for Glycomics and School of Environment and Science, Griffith University, Gold Coast, Queensland 4222, Australia; ∥ Institute of Computational Biology, Department of Biotechnology, University of Natural Resources and Life Sciences, Vienna, Muthgasse 18, 1190 Vienna, Austria; ⊥ Christian Doppler Laboratory for Molecular Informatics in the Biosciences, University of Natural Resources and Life Sciences, Vienna, Muthgasse 18, 1190 Vienna, Austria

## Abstract

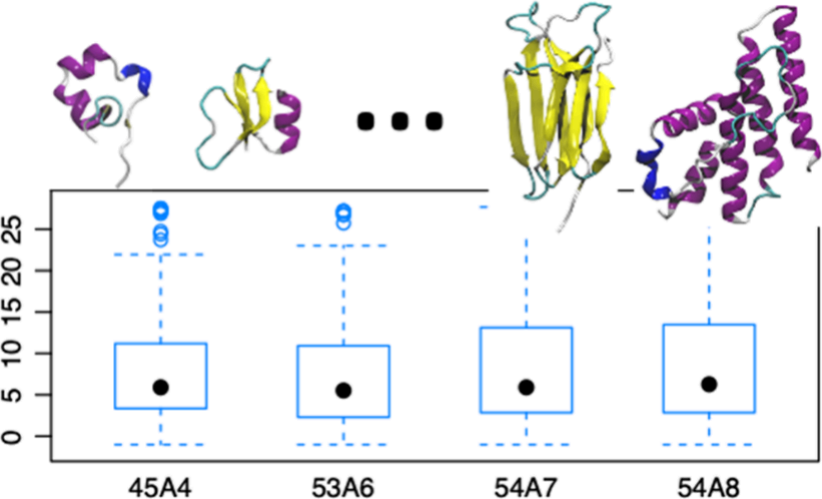

Molecular dynamics simulations depend critically on the quality of the force field used to describe the interatomic interactions and the extent to which it has been validated for use in a specific application. Using a curated test set of 52 high-resolution structures, 39 derived from X-ray diffraction and 13 solved using NMR, we consider the extent to which different parameter sets of the GROMOS protein force field can be distinguished based on comparing a range of structural criteria, including the number of backbone hydrogen bonds, the number of native hydrogen bonds, polar and nonpolar solvent-accessible surface area, radius of gyration, the prevalence of secondary structure elements, *J*-coupling constants, nuclear Overhauser effect (NOE) intensities, positional root-mean-square deviations (RMSD), and the distribution of backbone ϕ and ψ dihedral angles. It is shown that while statistically significant differences between the average values of individual metrics could be detected, these were in general small. Furthermore, improvements in agreement in one metric were often offset by loss of agreement in another. The work establishes a framework and test set against which protein force fields can be validated. It also highlights the danger of inferring the relative quality of a given force field based on a small range of structural properties or small number of proteins.

## Introduction

Molecular dynamics simulations of protein and peptide systems play an ever-growing role in academic and industrial research.^1^ The use of classical mechanics in conjunction with empirical force fields makes it possible to study processes ranging from the reversible folding of peptides to the functional motions of large protein complexes in atomic or near atomic detail. Such empirical force fields use simple analytical functions to describe the potential energy of the system in terms of the atomic coordinates. While the empirical force fields commonly used to simulate protein systems, such as CHARMM,^2^ AMBER,^3^ OPLS,^4^ and GROMOS,^5,6^ employ very similar functional forms to represent the bonded and nonbonded interactions, the strategies used to parametrize these functions vary significantly. In addition, all of the protein force fields listed above have been refined over decades. The parameters have been progressively adjusted in light of new theoretical calculations, the availability of additional experimental data, or the increase in computational power, which has enabled new properties or time scales to be examined. While clearly there have been improvements over time, there is no accepted benchmarking framework for determining whether a given parameter set is a fundamental improvement over another.

The testing and validation of protein force fields is challenging for several reasons. The primary difficultly is that empirical force field parametrization is a poorly constrained problem. Some properties can be exquisitely sensitive to small variations in a particular parameter, while other properties can appear quite insensitive. The parameters used in a given force field are also highly correlated. As a result, alternative parameter combinations can yield very similar results. This also means that varying one parameter may cause a range of other parameters to be no longer optimal.

A second challenge in the testing and validation of force fields is the choice of target properties. Parameters adjusted to reproduce the conformational properties of proteins in one environment may result in the same protein adopting an inappropriate conformation in a different environment. Indeed, some groups have proposed different parameter sets to describe proteins in a folded as compared to an unfolded state, despite the nature of the interactions and the solvent environment being identical.^7,8^ All of the protein force fields listed above have been proposed based primarily on their ability to reproduce a specific set of experimental or theoretical data. Not only does the nature of the data used to develop new versions differ between force fields, but so too does the weighting given to different types of data. This raises the question of what data are most appropriate for validation.

It is also important to consider that the theoretical and experimental data used in force field development and validation will themselves contain uncertainties. For example, experimental data can be direct or derived. In this context, direct data are quantities that can be directly observed by experiment, such as nuclear magnetic resonance (NMR) nuclear Overhauser effect (NOE) intensities, *J*-coupling constants, chemical shifts, residual dipolar couplings, X-ray reflection intensities, and vibrational spectra. Derived data, in contrast, are quantities that can be inferred from experimental data but cannot be measured directly. Examples include protein structural models, torsional angles, NMR order parameters, and NOE-derived interatomic distances. Ideally, direct experimental data would be favored. In practice, derived data such as structural models are often used due to the ability to make direct comparisons between a structural model derived from NMR or X-ray crystallography and conformations from a simulation.

While the use of direct experimental data is in principle to be preferred, there are still multiple challenges. Even when a quantity can be measured experimentally with high accuracy, calculation of the equivalent quantity from a simulation will involve a range of approximations. This, combined with a focus on a narrow range of observables, can lead to overfitting. For example, attempts have been made to validate or refine different parameter sets based on their ability to reproduce residual dipolar couplings, order parameters, and *J*-coupling constants.^9−11^ These parameter sets may show good or improved performance in regard to these specific observables but may show worse performance in regard to structural or thermodynamic properties. This is because the structural interpretation of variations in residual dipolar couplings, order parameters, and *J*-coupling constants are highly model-dependent.^1,12^ Even the comparison of interproton distances to experimental NOEs is dependent on the choice of motional model.^13^

Any force field that accurately represents the underlying potential energy surface is expected not only to reproduce a wide range of experimentally observable and derived structural properties but also to do so for a diverse and representative range of systems. The final challenge is whether the results obtained are statistically meaningful, how to weight the relative importance of different properties, and whether the results of any calculations can be independently validated. Developing a framework to deal with these challenges is the primary focus of the current study.

Attempts to compare the performance of different force fields and to validate them against experiment have long been limited by poor statistics. For example, in the original 1995 paper by Cornell et al.^3^ describing the AMBER ff94 force field, a single 180 ps simulation of ubiquitin in water formed a key part of the validation studies. The finding that the root-mean-square deviation (RMSD) from the crystal structure of all heavy atoms in the first 72 residues was 0.05 nm lower using ff94 compared to the previous parameter set (0.20 nm as opposed to 0.25 nm) was claimed to indicate a significant improvement. Although these simulations were long for their time, the difference in RMSD was clearly within uncertainty. In the same year, Smith et al.^14^ used the results of three 1 ns simulations of hen egg lysozyme (HEWL), one in vacuum and two in water, to calculate in addition to RMSD the radius of gyration, solvent-accessible surface area (SASA), and a range of NMR parameters, including NOEs, backbone ^1^H–^15^N order parameters, and ^3^*J*_HNα_-coupling constants. This work highlighted the difficulty of obtaining sufficient convergence to draw meaningful conclusions in regard to the reproduction of experiment. This finding was reinforced when Stocker and van Gunsteren were unable to reliably distinguish between the GROMOS87 37C4+ and GROMSOS96 43A1 force fields based on the same system.^15^

Price and Brooks^16^ simulated three proteins for 2 ns and concluded that CHARMM22, AMBER94, and OPLSAA were equally good. The widely used 2003 release of AMBER for proteins, which incorporated a new charge model, was validated for proteins based on its ability to distinguish between an experimentally derived model (X-ray or NMR) and a set of decoy structures for 54 unique proteins.^17^ Each trial simulation was just 10 ps in length and performed using a generalized Born model to represent solvation effects.^17^ Van der Spoel and Lindahl^18^ conducted one of the first validation studies involving extended simulation times and many replicates. They performed 28 × 50 ns simulations of the villin headpiece to compare the performance of the OPLS all-atom force field and the GROMOS united atom force field (43A1).^18^ Despite the size of this study, variations between the replicates made it difficult to distinguish between the force fields even for this very simple system.

In addition to short simulation times and small numbers of proteins, the range of proteins considered has historically also been narrow. The GROMOS 43A1, 45A3, and 53A6 parameter sets were all validated by computing a range of structural and NMR parameters for the protein HEWL from 2 to 5 ns simulations.^19−21^ The fact that the NMR structure of lysozyme performs well in the GROMOS family of force fields may not be surprising, as the NMR data were interpreted in part using the GROMOS force field. Likewise, the X-ray and NMR structures solved using X-plor might perform well using CHARMM force fields. In both cases, errors in the force fields are incorporated into the structure used for validation.

In 2007, Villa et al.^22^ attempted to address the effects of poor statistics and the focus on a narrow range of protein systems. They compared three versions of the GROMOS force field (43A1, 53A5, and 53A6) by performing 5–10 ns simulations of 31 proteins in triplicate. Although a wide range of structural properties were examined, including the RMSD from the experimental structure, radius of gyration, SASA, secondary structure retention, and hydrogen-bond propensities, they were unable to demonstrate that the observed differences between the force fields were statistically significant. The variations between proteins and between replicate simulations meant that the scale of the study was simply insufficient. These relatively measured conclusions can be contrasted against those of Lange et al.,^23^ who in 2010 ranked different variants of AMBER, CHARMM, GROMOS, and OPLS on their deviation of the protein from the starting X-ray structure and the ability to back-calculate a series of NMR-derived quantities, in particular residual dipolar couplings (RDCs). The analysis involved just two proteins (GB3 and ubiquitin) and a single 1 μs simulation of each combination of protein force field and long-range electrostatic treatment. The force fields were ranked based on how long into the simulation the average deviation of the back-calculated RDCs from experimental values remained small. Despite the uncertainties in the back-calculation of RDCs, they concluded that force fields that used particle mesh Ewald (PME) to evaluate long-range electrostatics were superior to those that used the reaction field method. This conclusion was reached despite the fact that the effect of a reaction field was only examined in the case of the GROMOS 43A1 and 53A6 parameter sets. In fact, the results presented in the paper show little difference in the results using PME or reaction field. What is shown is a marked difference between the use of PME and a straight cutoff, a result which is as expected.

The reliable calculation of experimental observables for a given protein and force field requires simulations that are long enough to sufficiently sample all the accessible molecular configurations at the temperature and pressure of interest. For some small peptides, it is possible to sample a large number of folding and unfolding events on submicrosecond time scales.^24^ However, for even the smallest proteins, motions over milliseconds often play important functional roles.^25^ Lindorff-Larsen et al.^26^ performed 1.2 μs simulations of four proteins (HEWL, bovine pancreatic trypsin inhibitor (BPTI), ubiquitin, and the B3 domain of protein G (GB3)) in order to validate proposed changes in the torsion parameters in AMBER. They showed improvements in the root-mean-square error of ^3^*J*_HαCαCβHβ_-coupling constants and RDCs but no other properties. The Shaw group also used their special-purpose machine ANTON^27^ to compare the effect of force field on the folding propensity of the villin headpiece.^28^ This involved 100 to 300 μs simulations using AMBER ff03 and ff99SB*-ILDN as well as a local variant of CHARMM22 and CHARMM27. This was perhaps the first study to be on a time scale sufficient to sample most relevant conformational states. Notably, however, the results for each force field corresponded to a different temperature, varying by as much as 60 K. None corresponded to the temperature at which the experimental data were collected. Three of the four were above the boiling point of water at 1 atm.

In one of the few studies involving a wide variety of proteins simulated under comparable conditions, Li and Brüschweiler^10,29^ used a series of 100 ns simulations for a set of six trial and 17 validation proteins in an attempt to refine AMBER torsional parameters based on the ability to predict RDCs, order parameters, and *J*-coupling constants. Schmid et al.,^30^ in testing the GROMOS 54A7 parameter set, used duplicate 50 ns simulations of four systems (HEWL, fox1 RNA binding protein, chorismate mutase, and the peptide GCN4-P1) and considered a range of structural properties, including RMSD, secondary structure propensity, NOE violations, and *J*-coupling constants. The 54A8 parameter set with revised ion and charged residues was tested on a total of six proteins, each simulated for 20 to 100 ns.^31^ Huang and MacKerell^32^ used six proteins and simulations of between 100 ns and 1.2 μs to compare the performance of CHARMM36 against a range of NMR parameters but no other structural parameters. In the case of OPLS-AA, only two proteins (ubiquitin and GB3) were used in the validation of a new set of torsion parameters, with a single 200 ns simulation being performed in each case and only results for *J*-coupling constants being presented.^33^ In similar work, Maier et al.^34^ used four proteins (GB3, ubiquitin, BPTI, and HEWL) and a limited range of NMR parameters to validate AMBER ff14SB. For OPLS3, distributed by Schrödinger, Inc., the average RMSD after 200 ns of simulation for seven relatively small and stable proteins was provided as evidence of a significant improvement over earlier OPLS versions.^35^ In this case, Harder et al.^35^ reported performing the simulations in triplicate. However, only the mean RMSD of the final 100 ns averaged over the three replicates was shown. The variability between the replicates, which is crucial to understand the statistical significance of the variation between force fields, was not given. The work of Robustelli et al.^11^ also deserves mention. The paper primarily focuses on results of microsecond simulations of a proposed benchmark set of 21 systems, of which only four are proteins with well-defined tertiary structures (GB3, ubiquitin, BPTI, and HEWL). However, as part of an extensive Supporting Information, results from multimicrosecond simulations of a wide range of folded proteins using alternative parameter sets are also presented but not analyzed in depth.

While the more recent validation studies routinely simulate microseconds of protein dynamics, a range of fundamental errors continue to be made.^1^ Many studies do not perform simulation replicates in order to mitigate the lack of convergence and assess the degree of variability between runs. Few include a wide variety of protein structures in order to demonstrate transferability of the force field. In some cases there are internal inconsistencies, such as comparison of simulations that were performed under different conditions (different thermostats, temperatures, cutoffs, etc.).^28,36^ The key point is that the original validation studies of the force fields currently in widespread use often only involved a handful of proteins, most frequently HEWL, GB3, and ubiquitin. GB3 and ubiquitin are notable, as they have highly stable folds and very similar structures.^37^ They are not representative of most systems of interest, and the reproduction of the experimental observations of such highly stable proteins is a necessary but not sufficient test of force field quality.^1,38^

Here we ask the question whether the results of previous validation studies involving the structural properties of proteins are likely to be statistically significant and consider what might be required to determine the relative quality of alternative force fields based on structural criteria, given variations between proteins and variations between individual simulations. To provide data with which to illustrate the challenges involved and the types of statistical analysis that can be performed, a wide range of proteins varying both in size and structural properties were simulated under identical conditions using four variants of the GROMOS force field, namely, the 45A4,^39^ 53A6,^20^ 54A7,^30^ and 54A8^31,40^ parameter sets. A range of metrics were considered. These included the number of backbone hydrogen bonds, the number of native hydrogen bonds, polar and nonpolar SASA, radius of gyration, the prevalence of secondary structure elements, *J*-coupling constants, NOE intensities, positional RMSD, and the distribution of backbone ϕ and ψ dihedral angles. These properties were considered because they can be expressed as a deviation from a proposed experimental structure and are commonly used to judge the quality (or otherwise) of an individual simulation.

Note that although the total simulation time considered is significant (>8 μs), we deliberately limited individual runs to be on the same order as those runs used as part of the initial development and validation of the parameter sets examined. This is much less than is required to demonstrate convergence. The goal of this work is not to propose that a particular parameter set is fundamentally better than another but rather to establish a framework for statistical analysis and propose a comprehensive test set against which the structural properties of protein force fields can be validated. The comparison of alternative variants of the GROMOS force field is presented as an example to highlight specific challenges and potential bias without impugning the work of others.

## Methods

**Figure 1:**
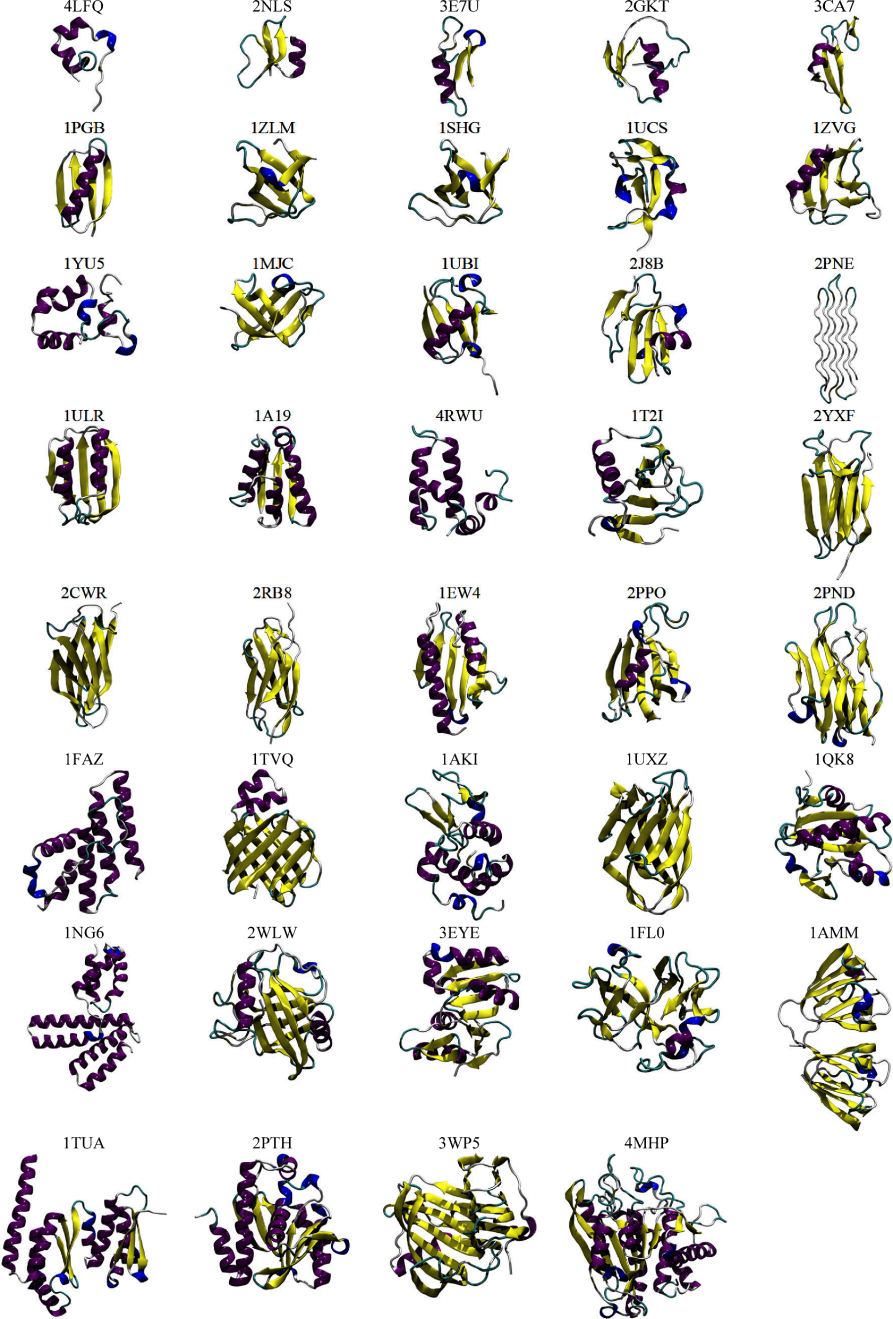
Cartoon depictions of the starting structures derived from X-ray crystallographic data (purple, α-helix; yellow, β-strand; blue, 3_10_-helix; red, π-helix; cyan, turn; white, coil).

**Figure 2:**
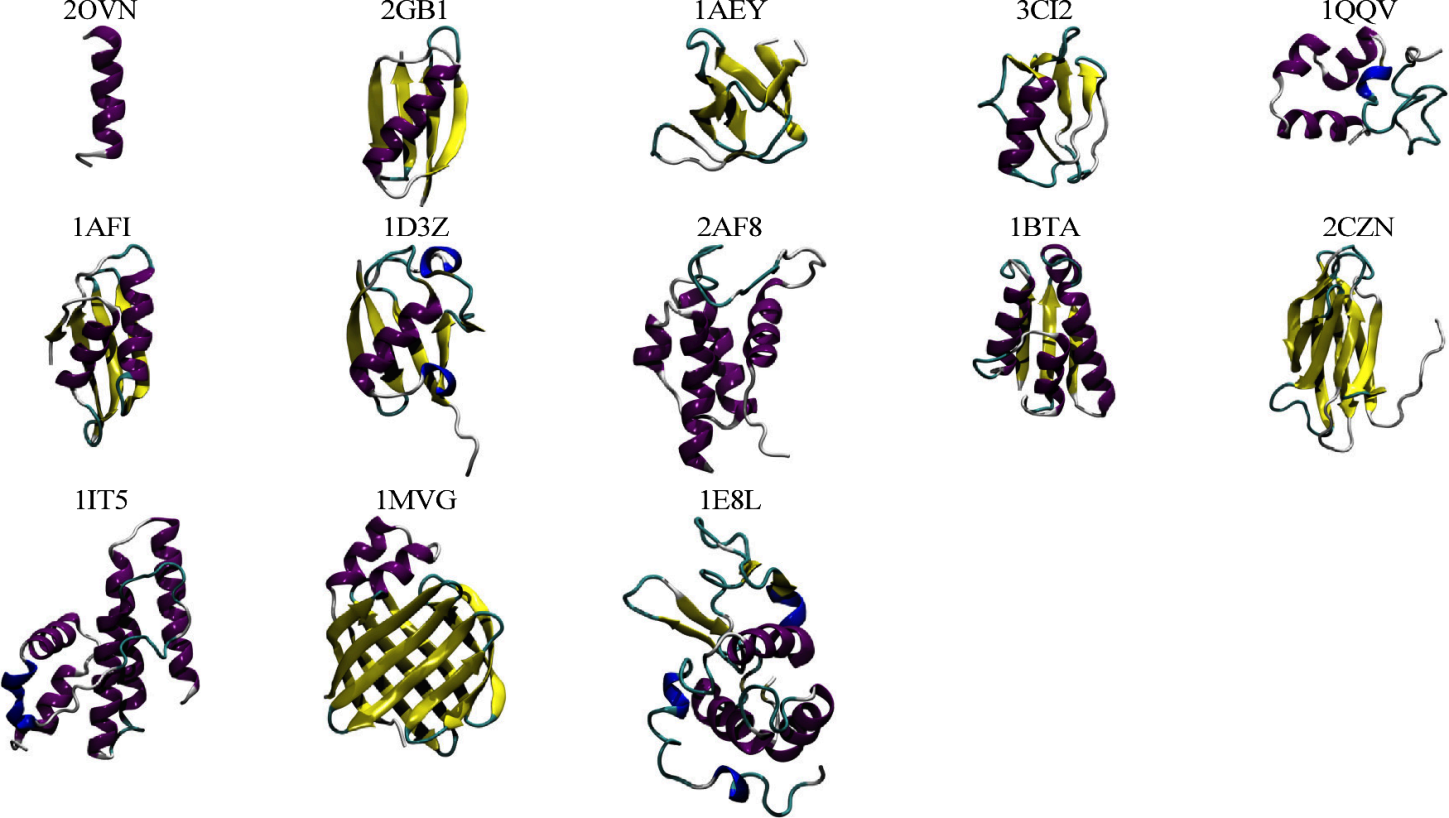
Cartoon depictions of the starting structures derived from NMR data (purple, α-helix; yellow, β-strand; blue, 3_10_-helix; red, π-helix; cyan, turn; white, coil).

A total of 52 protein structures were examined, corresponding to 42 different proteins ranging in size from 17 to 326 residues. This included 39 structures solved by X-ray diffraction (Table 1 and Figure 1) and 13 solved using NMR techniques (Table 2 and Figure 2). All the corresponding proteins are monomeric in solution, do not contain ligands or cofactors (including ions), and are not bound to other macromolecules such as DNA or RNA. An initial set of 32 structures were chosen based on completeness and a resolution of below 1.5 Å. An additional nine proteins were included for which both an X-ray structure and an NMR structure were available. A final structure, that of the major cold shock protein (PDB ID 1MJC) was included to facilitate comparison with a previous validation study.^31^ Reference structures solved by X-ray diffraction and NMR are listed in Tables 1 and 2, respectively. Unless stated otherwise, the experimental data pertaining to each structure were obtained from either the PDB^41^ or the Biological Magnetic Resonance Data Bank.^42^

**Table 1:** List of the 39 Protein Structures Solved by X-ray Diffraction Used in This Study^a^

PDB ID	protein name	organism	NR	3_10_ (%)^b^	α (%)^b^	β (%)^b^	pH	res. [Å]	*R*_work_	*R*_free_	force field
4LFQ	potassium channel toxin L-ShK	*Stichodactyla helianthus*	35	9	34	–	7.0	1.1	0.13	0.16	all
2NLS	human β-defensin-1, mutant Q24A	*Homo sapiens*	36	–	19	31	7.5	1.0	0.11	0.12	54A7/8
3E7U	plectasin	*Pseudoplectania nigrella*	40	3	23	25	7.5	1.4	0.16	0.18	all
2GKT	turkey ovomucoid third domain	*Meleagris gallopavo*	51	–	20	16	7.5	1.2	0.13	0.15	all
3CA7	protein Spitz	*Drosophila melanogaster*	52	–	12	38	6.5	1.5	0.2	0.24	54A7/8
1PGB^c^	protein G, B1 domain	*Streptococcus* sp. gx7805	56	–	25	43	4.5	1.9	0.20	–	all
1ZLM	osteoclast stimulating factor 1	*Homo sapiens*	58	5	–	48	7.5	1.1	0.16	0.21	all
1SHG^c^	α-spectrin	*Gallus gallus*	62	5	–	49	4.0	1.8	0.2	0.28	all
1UCS	antifreeze peptide RD1	*Lycodichthys dearborni*	64	14	6	25	7.5	0.6	0.14	0.15	all
1ZVG	α-like neurotoxin BmK-I	*Mesobuthus martensii*	66	2	15	30	6.0	1.2	0.16	0.16	all
1YU5^c^	villin headpiece	*Gallus gallus*	67	10	45	–	7.0	1.4	0.19	0.22	all
1MJC	major cold shock protein	*Escherichia coli*	69	4	–	46	7.5	2.0	0.19	–	all
1UBI^c^	ubiquitin	*Homo sapiens*	76	8	16	33	5.6	1.8	0.17	–	all
2J8B	human CD59 glycoprotein	*Homo sapiens*	79	8	10	32	7.5	1.1	0.17	0.2	54A7/8
2PNE^d^	glycine-rich antifreeze protein	*Hypogastrura harveyi*	81	–	–	–	6.5	1.0	0.14	0.16	all
1ULR^e^	putative acylphosphatase	*Thermus thermophilus*	88	1	28	38	7.0	1.3	0.19	0.22	all
1A19^c^	barstar, mutant C82A	*Bacillus amyloliquefaciens*	90	2	44	17	6.5	2.8	0.2	0.29	54A7/8
4RWU	protein Sis1	*Saccharomyces cerevisiae* s288c	92	–	62	–	7.0	1.2	0.12	0.15	54A7/8
1T2I	ribonuclease Sa	*Streptomyces aureofaciens*	96	3	11	22	7.2	1.1	0.13	0.17	all
2YXF	β-2-microglobulin	*Homo sapiens*	100	–	–	49	7.0	1.1	0.18	0.2	54A7/8
2CWR^c^	chitin binding domain of chitinase	*Pyrococcus furiosus*	103	–	–	62	6.5	1.7	0.19	0.23	all
2RB8	tenascin	*Homo sapiens*	104	–	–	48	7.5	1.4	0.17	0.2	54A7/8
1EW4	CyaY	*Escherichia coli*	106	–	31	32	5.1	1.4	0.19	0.21	all
2PPO	FK506 binding protein-12 (FKBP12), mutant E60A	*Homo sapiens*	107	4	7	37	7.0	1.3	0.13	0.18	all
2PND	Murine CRIg	*Mus musculus*	119	5	–	51	7.5	1.0	0.12	0.14	54A7/8
1FAZ^c^	phospholipase A2	*Streptomyces violaceoruber*	122	10	57	–	6.0	1.4	0.19	0.23	all
1TVQ^c^	chicken liver basic fatty acid binding protein	*Gallus gallus*	125	–	11	58	7.5	2.0	0.23	0.27	all
1AKI^c^	lysozyme	*Gallus gallus*	129	13	30	11	4.5	1.5	0.21	–	all
1UXZ	cellulase B	*Cellvibrio mixtus*	131	2	–	56	7.0	1.4	0.16	0.18	all
1QK8	tryparedoxin-I	*Crithidia fasciculata*	146	8	27	22	7.5	1.4	0.19	0.22	all
1NG6	hypothetical protein yqeY	*Bacillus subtilis*	148	4	74	–	5.5	1.4	0.21	0.25	all
2WLW	TRIM5-CypA	*Macaca mulatta*	165	3	12	32	7.4	1.5	0.16	0.19	54A7/8
3EYE	PTS system *N*-acetylgalactosamine-specific IIB component 1	*Escherichia coli* o157:h7	168	5	35	24	7.0	1.4	0.19	0.22	54A7/8
1FL0	endothelial monocyte-activating polypeptide II	*Homo sapiens*	171	7	2	37	7.5	1.5	0.22	0.22	all
1AMM	γ-crystallin B	*Bos taurus*	174	6	3	40	6.8	1.2	0.18	–	all
1TUA^e^	hypothetical protein APE0754	*Aeropyrum pernix*	191	7	53	18	7.0	1.5	0.21	0.23	all
2PTH	peptidyl-tRNA hydrolase	*Escherichia coli*	193	7	38	21	7.5	1.2	0.2	0.21	54A7/8
3WP5	xylanase CDBFV, mutant E109A	*Neocallimastix patriciarum*	227	2	8	54	6.5	1.3	0.15	0.18	all
4MHP	putative glutaminyl cyclase	*Ixodes scapularis*	326	6	33	17	7.5	1.1	0.17	0.19	all

a Abbreviations: NR = number of residues; 3_10_ = 3_10_-helix; α = α-helix; β = β-strand + β-bridge.

b Secondary structure percentages as assigned by DSSP.

c Has a paired NMR structure.

d Polyproline II spiral only, not detected by DSSP.

e pH not reported, set to 7.0.

**Table 2:** List of the 13 NMR Protein Structures Used in This Study^a^

PDB ID	protein name	organism	NR	3_10_ (%)^b^	α (%)^b^	β (%)^b^	pH	NMR data	force field
2OVN	GCN4 trigger peptide (p16-31)	*Saccharomyces cerevisiae*	17	–	76	–	7.5	NOE, Jval	all
2GB1^c^^,^^d^	protein G, B1 domain	*Streptococcus* sp. “group g”	56	1	18	44	7.0	–	all
1AEY^c^	α-spectrin	*Gallus gallus*	62	5	–	48	3.5	–	all
3CI2	chymotrypsin inhibitor 2 (CI-2)	*Hordeum vulgare*	66	–	17	17	4.2	NOE, Jval	all
1QQV^c^	villin headpiece	*Gallus gallus*	67	7	37	–	7.0	NOE, Jval	all
1AFI	mercury binding protein (MerP)	*Shigella flexneri*	72	–	29	28	6.5	NOE	all
1D3Z^c^	ubiquitin	*Homo sapiens*	76	8	16	33	6.5	NOE, Jval, RDC	all
2AF8	actinorhodin polyketide synthase acyl carrier protein	*Streptomyces coelicolor*	86	–	50	–	4.9	–	54A7/8
1BTA^c^	barstar	*Bacillus amyloliquefaciens*	89	3	42	18	6.7	Jval	all
2CZN^c^	chitin binding domain of chitinase	*Pyrococcus furiosus*	103	-	–	51	5.6	NOE	all
1IT5^c^	phospholipase A2	*Streptomyces violaceoruber*	122	11	57	–	7.6	–	all
1MVG^c^	chicken liver basic fatty acid binding protein	*Gallus gallus*	125	1	12	59	5.6	NOE, Jval	all
1E8L^c^	lysozyme	*Gallus gallus*	129	2	29	6	3.8	NOE, Jval, RDC	all

a Abbreviations: NR = number of residues; 3_10_ = 3_10_-helix; α = α-helix; β = β-strand + β-bridge; NOE = nuclear Overhauser effect; Jval = ^3^*J*-coupling; RDC = residual dipolar coupling.

b Secondary structure percentages as assigned by DSSP.

c Has a paired X-ray diffraction structure.

d pH not reported, set to 7.0.

### Structure Preparation

The first model provided in the PDB file was used as the starting structure for both the crystal and NMR-derived structures. Where residues showed multiple occupancy in the X-ray structures, a single conformation was selected by visualizing the alternative conformations and choosing a combination of occupancies that avoided atomic clashes. All water molecules contained within the modeled structures were retained. PROPKA3^43^ as implemented in PDB2PQR (version 2.1.0, PARSE force field^44^) was used to assign the protonation state of titratable residues. The pH used to determine the protonation state of titratable side chains was either taken from the PDB file (“PH” entry) or extracted from the relevant publication. Where the pH could not be determined (2GB1, 1TUA, and 1ULR), a value of 7.0 was used. The C- and N-termini were taken to be charged in all structures. The preparation of the systems was performed using the GROMOS++ package (version 1.4.0).^45^ Missing atoms were added, and their positions were energy-minimized using a steepest decent algorithm. During this procedure, all atoms present in the reference structure were positionally constrained. The protein and any crystal waters were placed in a rectangular periodic box such that the minimum distance to the box wall was 1.2 nm. The system was then solvated in SPC water and energy-minimized with all protein atoms positionally restrained, with a force constant of 25 × 10^3^ kJ mol^–1^ nm^–2^. Na^+^ and/or Cl^–^ ions were added by randomly replacing water molecules lying at a distance of 0.4 nm or greater from any protein atom or existing ion. This process was repeated until sufficient ions were added to balance the net charge of the protein and ensure a physiological salt concentration of 0.15 M. If additional ions could not be added using a cutoff distance of 0.4 nm, the cutoff was progressively reduced in steps of 0.01 nm and the process repeated.

### MD Simulations

All MD simulations were performed using the GROMOS11 software package.^46^ Unless stated otherwise, the temperature and pressure were set to 298 K and 1 atm, respectively. The weak-coupling scheme^47^ was used to maintain the temperature and pressure with relaxation times of 0.1 and 0.5 ps, respectively. Protein atoms were coupled to a separate temperature bath to the solvent and ions. A group-based twin-range cutoff scheme for nonbonded interactions was employed, with a short-range cutoff of 0.8 nm and a long-range cutoff of 1.4 nm. To reduce the effects of truncating the electrostatic interactions beyond the 1.4 nm cutoff, a reaction field correction^48^ was applied for which the relative permittivity was set to 61.^49^ The pair list was updated every five steps. The leapfrog algorithm was used to integrate Newton’s equations of motion using a time step of 2 fs. Bond lengths were constrained using the SHAKE algorithm.^50^ Center-of-mass motion was removed every 1000 steps.

The solvated structures were equilibrated by heating the system from 50 to 298 K while simultaneously reducing harmonic position restraints on the protein. This was done by performing a series of six constant-volume simulations, each 20 ps in length, increasing the temperature by 50 K between each simulation (48 K on the last). At the same time, the position restraint force constant was reduced from 25 × 10^3^ kJ mol^–1^ nm^–2^ to 0 in 5 × 10^3^ kJ mol^–1^ nm^–2^ increments. An additional 20 ps simulation was performed to initialize roto-translational constraints on the solute atoms,^51^ and a final 20 ps simulation was performed with pressure coupling at 1 atm. Thus, each system was relaxed over a total of 160 ps. Subsequent production simulations were performed in triplicate for 15 ns using different initial velocities. Analysis was performed on the last 5 ns of each simulation using configurations written every picosecond.

### Parameter Sets

Four variants of the GROMOS force field were considered in this work, namely, the 45A4,^39^ 53A6,^20^ 54A7,^30^ and 54A8^31,40^ parameter sets. The GROMOS force field has been parametrized to reproduce the structural and thermodynamic properties of biomolecules such as peptides, nucleic acids, lipids, and sugars. It has been fitted against the densities and heats of vaporization of simple liquids containing functional groups found in biomolecules as well as the free energy of solvation of a set of reference molecules in SPC water and cyclohexane. The 45A4 parameter set was the last iteration of the 4x parameter set, which was first released in 1996. The “45” refers to the number of Lennard-Jones atom types, the letter “A” indicates that the parameters are for a condensed-phase system, and the number “4” signifies the fourth revision of the force field. The 53A6^20^ parameter set was released 2004. In addition to eight new atom types, the Lennard-Jones parameters and partial charges of polar groups were reparametrized to better reproduce the solvation free energies in water and cyclohexane for analogues of the side chains of the 14 neutral amino acids. The 54A7^30^ parameter set included a reparametrization of the peptide backbone dihedrals. Finally, 54A8^40^ added refinements to the Lennard-Jones parameters and partial charges of the charged amino acid side chains.

### Hydrogen Bonds

A hydrogen bond was considered to exist if the distance between the hydrogen and acceptor was below 0.25 nm and the donor–H–acceptor angle was larger than 120°.

### SASA

The solvent-accessible surface area was calculated using a probe radius of 0.14 nm.^52^ The radii of the protein (solute) atoms were assumed to correspond to the minimum in the Lennard-Jones potential function describing the interaction between the oxygen of SPC water and the solute atom. In this work, arginine, aspartic acid, asparagine, glutamic acid, glutamine, glycine, histidine, lysine, serine, threonine, and proline were considered to be polar residues. Alanine, cysteine, isoleucine, leucine, methionine, phenylalanine, tryptophan, tyrosine, and valine were considered to be nonpolar.^53^

### Radius of Gyration

The mass-weighted radius of gyration was calculated as
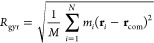
1where *m*_*i*_ is the mass and **r**_*i*_ the position vector of atom *i*, **r**_com_ is the position vector of the center of mass, and *M* is the total mass of all *N* atoms.

### Secondary Structure

The secondary structure was assigned using the Dictionary of Secondary Structures of Proteins (DSSP) criteria proposed by Kabsch and Sander.^54^

### Positional RMSD

The positional root-mean-square-deviation was calculated after performing a least-squares fit on the protein backbone (heavy atoms) excluding the first and the last residue, using
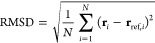
2where **r**_*i*_ and **r**_ref,*i*_ are the position vectors of atom *i* in the test and the reference conformations, respectively, and *N* is the total number of atoms. As the significance of the magnitude of the RMSD between two conformations is dependent on the size of the protein, the RMSD_100_ as proposed by Carugo and Pongor^55^ was also calculated:
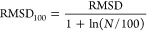
3The RMSD_100_ attempts to normalize the RMSD (eq 2) to a value equivalent to that of a protein with 100 residues. This approach has not been validated for structures with fewer than 40 residues, so the RMSD values for proteins with 40 residues or less were not adjusted.

### ^3^*J*-Coupling Constants

^3^*J*-coupling constants were estimated from the associated dihedral angles using the Karplus relation:^56^
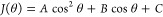
4where the empirical parameters *A*, *B*, and *C* vary depending on which heavy atoms are used to define the dihedral.^57^ A large variety of alternative empirical parameter sets have been proposed for use with the Karplus curve, with different studies using different sets of reference structures and different sets of NMR data and incorporating different corrections for motional averaging.^58−61^ In this work, *J*(θ) calculations were performed using the parameters proposed by Lindorff-Larsen et al.^60^ Note that as the united atom GROMOS force field was used, Φ was defined in terms of the atoms C–N–C_α_–C. The resulting dihedral angle used in the Karplus relation was θ = Φ – 60°.

### NOE Intensities

To compare experimental NOE data to the results from simulations, one should in principle compare the buildup of NOE intensities to the magnitude of the spectral density (including relaxation effects) as a function of time. However, for the cases considered in this work, only upper or upper and lower distance bounds are available. These distance bounds have been inferred from NOE intensities assuming the system is freely rotating, that a single rotational correlation time can be used to describe the motion of the entire protein, and that the intensity of the NOE at a given mixing time is proportional to ⟨*r*^–6^⟩, where *r* is the interproton distance and the angle brackets indicate an ensemble average. Here, given the short time scales examined in the simulations, the ⟨*r*^–3^⟩^–1/3^ averaged distances from the simulations are compared to the proposed experimental upper bounds, in line with the work of Tropp.^13^ Violations were computed by averaging over the replicates. To correct for cases where the assignment is ambiguous, the pseudoatoms proposed by Wüthrich were used.^62,63^ Where other corrections had been applied, these were first removed and replaced by the pseudoatom corrections.

### Statistical Treatment

Ensemble averages for secondary structure propensities, radius of gyration, SASA, and the number of backbone hydrogen bonds were computed and compared to the corresponding quantities obtained from the proposed experimental model. Unless otherwise noted, each replicate was treated separately before the results were combined and averaged.

To determine whether the observed differences in a particular property were significant given the size of the data set (number of proteins) and variations between individual runs, a similar approach to that proposed by Villa et al.^22^ was employed. In that work, multiple replicates and multiple proteins were used in an effort to remove the protein-specific contribution to the variance in the properties examined in order to test whether any observed differences were due to the force field. A fixed-effects multivariate analysis of variance (MANOVA) was then used to determine the influence of the force fields on the variance. However, the differences in a particular metric between alternative parameter sets will be affected by both the variability due to the choice of protein and the variability between replicates. The data therefore have three levels of variability: the effect of the parameter set, the effect of the protein, and the variability between replicates. Given this, a more appropriate statistical treatment is to use a multivariate mixed effects model, as is for example proposed in ref (64).

The work of Villa et al.^22^ was therefore extended by considering a nested representation of the data where the replicate simulations are nested within each of the proteins and the latter are nested within a given parameter set. This representation requires the consideration of mixed effects in the final regression models.^64^ The choice of protein and replicate are treated as two random effects with different variabilities. All parameter settings used to perform the simulations (the parameter set) were considered fixed effects. To perform the analysis, the properties used to characterize a particular simulation were first re-expressed as a one-dimensional “metric” value denoting a particular observation *y* of a given property. The type of metric which is represented by these *y* values was coded by a separate factor variable. To obtain a complete representation of the original multivariate data, we recorded the parameter set, the protein, and the replicate number. One advantage of this representation is that it allows for unbalanced situations where some values are missing. We refer below to this assessment as multivariate mixed effects likelihood ratio test (MVMELRT).

When performing a mixed model linear analysis, it is required that the residuals be Gaussian-distributed. To enforce this requirement, all metrics that were not Gaussian-distributed were transformed using an appropriate Box-Cox transformation.^65^ For this, the protein and the parameter set were used as regressors. To determine the heteroscedasticity of particular metrics as well as any correlation between metrics, the linear mixed effects regression model *lme* implemented in the statistical package R (*nlme*) was used. Whether the effect of the parameter set has a significant influence on a given metric vector was then determined by a likelihood ratio test. The heteroscedasticity and correlations were determined using the *weights* and *corr* parameters in *lme,* respectively. The nesting between protein and replicate was considered by regarding them as random effects in *lme*.

Whether any observed dependence of a given metric or set of metrics on the choice of parameter set was significant was tested by fitting two *lme* models: a simple model that disregarded the influence of the parameter set and a more complex model including the influence of the parameter set. Both models were fitted using a maximum likelihood approach. A subsequent likelihood ratio test provided a *p* value, which was used to assess whether a given metric vector had a significant dependence on the parameter set. Once significance was established, a more specific analysis of significant interactions between force field and protein characteristics could be obtained with a univariate mixed effects model. For this analysis, the R package *lme4* was used for modeling and the package *emmeans* was used for pairwise comparisons of parameter sets. Since this analysis gives rise to multiple tests, all *p* values were adjusted to Benjamini–Yekutieli false discovery rates (FDRs).^66^ We refer to this assessment as Benjamini–Yekutieli adjusted mixed effects likelihood ratio test (BYMELRT).

As noted above, all the statistical analysis was performed using R, version 4.1.2 (2021-11-01). Full details are provided in the Supporting Information. Furthermore, to ensure complete transparency and reproducibility of our results the raw data files plus the R code and other commands needed to repeat the analysis are also provided in the Supporting Information and through a GitHub repository (https://github.com/psykacek/rcode4ff).

## Results

In this work, the extent to which it is possible to assess the relative performance of alternative versions of the GROMOS force field based on structural criteria is considered. Following the approach of Villa et al.,^22^ multiple replicates and multiple proteins were used in an effort to remove any protein-specific contributions to the observed variance in the properties examined, i.e., to distinguish differences due to the choice in force field from the effects due to alternative initial conditions or the specific proteins simulated. Of the 52 protein structures considered, a total of 40 structures were simulated with all parameter sets (45A4, 53A6, 54A7, and 54A8) for 15 ns in triplicate. A further 12 structures were simulated for 15 ns in triplicate using just 54A7 and 54A8. This provided a sample size of 120 for the 45A4 and 53A6 parameter sets and 156 for the 54A7 and 54A8 parameter sets. All systems were simulated under identical conditions. In total the analysis presented is based on 8.3 μs of simulation time. To quantify the relative performance of the different parameter sets, a range of metrics were considered. Properties such as the number of native hydrogen bonds, the radius of gyration, and *J*-coupling constants were chosen, as they can be expressed as a deviation from a proposed experimental structure. These properties are also often used as metrics to judge the quality (or otherwise) of an individual simulation.

The first question that must be asked is whether the differences in the simulations obtained using the four different parameter sets are statistically significant given the number of proteins, the number of replicates, and the length of time simulated. The answer to this question is overwhelmingly yes. Combining all data using MVMELRT and performing a multivariate multilevel analysis, the probability that the differences observed are due to chance is very low (*p* ≪ 0.0001). The deviations are rapid, systematic, and dependent on the force field. The effect of the force field is evident despite the relatively short simulation times, the fact that the simulations were initiated from the same initial structures, and the small number of replicates, all of which would suggest that the true variance is severely underestimated. An underestimation of the variance in properties calculated using finite trajectories is a general problem when analyzing MD simulations. Unless the simulation is ergodic and the Boltzmann probabilities of all relevant states are well-converged, the variance will depend on the precise starting configuration (coordinates and velocities) as well as the length of the simulation(s). Without complete sampling, the variance will be underestimated or at least contain a degree of uncertainty. Furthermore, any uncertainty in the variance implies uncertainty in the *p* value. However, unlike the variance, which is an absolute measure, the *p* value listed above corresponds to a relative measure. It is based on a comparison of variances as opposed to the variance itself. Thus, the uncertainty in the *p* value can be mitigated by ensuring that the trajectories analyzed are of equal length and performed under similar conditions. This is a prime reason why only the initial velocities were varied between replicate runs. Nevertheless, it is important to stress that the *p* values have a high degree of uncertainty and should not be overinterpreted. They are presented primarily to illustrate how the proposed framework to compare force fields can be used.

Considering each of the metrics individually by a mixed effects model likelihood ratio test, there was more variation between the effects of the choice of parameter set. The effect of the choice of parameter set on the number of backbone hydrogen bonds, the number of native backbone hydrogen bonds, the polar and nonpolar SASA, the radius of gyration, RMSD_100_, and the proportions of α-helix and β-strand was highly significant, with *p* < 0.0001. The effect on other metrics, including the proportions of π-helix, 3_10_-helix, and β-bridge and both NOE and *J*-coupling constants was marginal (0.03 < *p* < 1.0). Pairwise comparisons between alternative parameter sets and individual metrics tell a different story again. Specific parameter sets affect a given metric much more than others. These differences are described in detail below. In order that a reader can compare the values of the specific metrics provided directly to what they would obtain from their own simulations, the figures and tables presented in the main paper contain untransformed data. All statistical analysis was, however, performed using Box-Cox-transformed data. Individual values for transformed and untransformed data are provided in the Supporting Information (data files S2, S3, and S4).

### Structural Metrics

**Figure 3:**
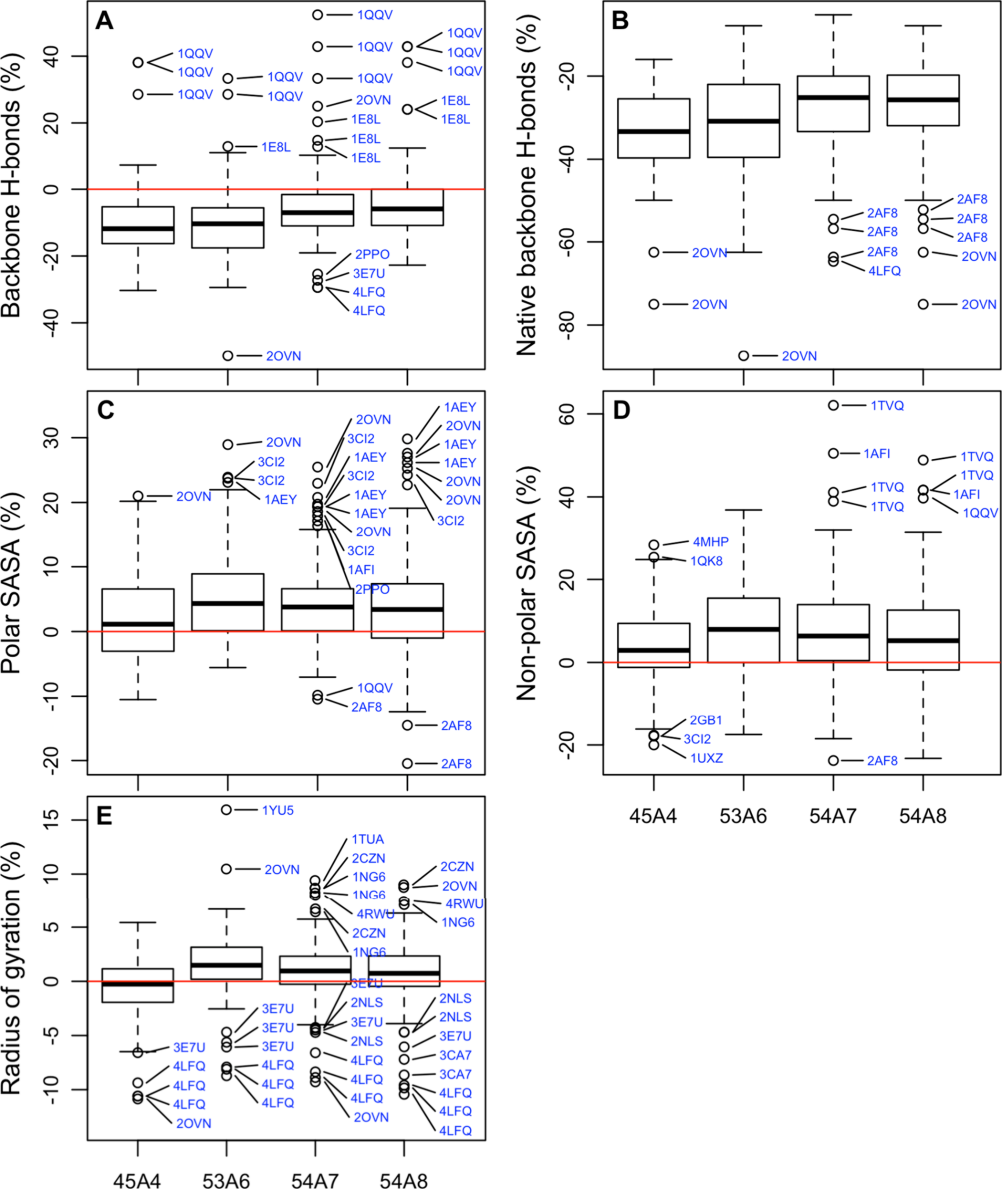
Boxplots of the percentage change in (A) the number of backbone hydrogen bonds, (B) the number of native backbone hydrogen bonds, (C) the total polar solvent accessible surface area (SASA), (D) the total nonpolar SASA, and (E) the radius of gyration for all proteins simulated with a given parameter set compared to the corresponding starting conformation (see the text). Outliers are marked with blue text. An outlier was defined as a value which lies above or below the quartile boundary plus 1.5 times the interquartile range. Multiple occurrences of the same code refer to replicas.

**Figure 4:**
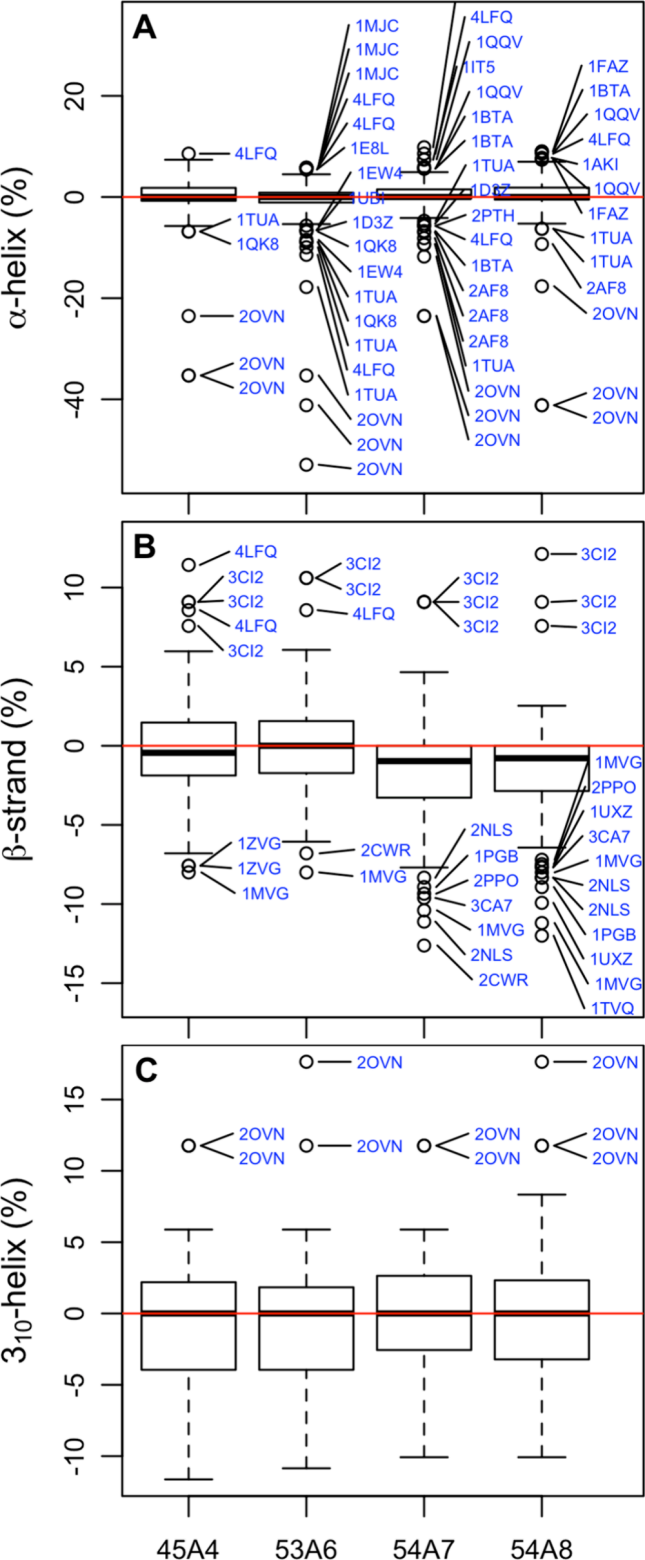
Boxplots of the percentage difference between the fraction of the sequence assigned to an individual element of secondary structure by the program DSSP in the simulation compared to the corresponding starting conformation for (A) α-helix, (B) β-helix, and (C) 3_10_-helix. Note: to avoid overlap in (A), only outliers 2.25 times the interquartile range or greater are shown.

The most straightforward of the metrics listed above to examine are structural metrics, which can be compared directly to the equivalent value calculated from the experimental structure using the same theoretical model. These include the number of backbone hydrogen bonds, the number of native backbone hydrogen bonds, the polar and nonpolar SASA, the radius of gyration, and the presence of specific elements of secondary structure (α-helix, β-strand, and 3_10_-helix). For each simulation, the percentage deviation from the value of the quantity *q* in the experimental structure, Δ*q*^%^, was calculated with respect to the average over the last 5 ns of each simulation:
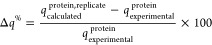
Boxplots of these percentage differences for each of the parameter sets for the number of backbone hydrogen bonds, the number of native backbone hydrogen bonds, the polar and nonpolar SASA, and the radius of gyration are shown in Figure 3. The prevalence of the main elements of secondary structure are shown in Figure 4. In addition, the pairwise differences, Δμ, between the absolute value of the mean of a given metric, μ = mean(Δ*q*), considering all proteins and all replicates obtained using the alternative parameter sets are presented in Table 3. Note, Δ*q* is the difference between the metric from the simulation and the reference. In Table 3, a positive number indicates that the value obtained in the simulation is closer to that calculated from the starting structure or measured experimentally for the newer of the two parameter sets compared. Also presented in Table 3 is an assessment of whether the differences between the values obtained using alternative parameter sets for that specific metric are significant. Box-Cox-transformed data on these and additional metrics (β-bend and π-helix) as well as boxplots of the transformed data are provided in Supporting Information (data file S1).

**Table 3:** Difference in the Absolute Value of the Mean Error Obtained Using Different Parameter Sets for the Properties Considered^a^^,^^b^

	54A7 → 54A8	53A6 → 54A8	45A4 → 54A8	53A6 → 54A7	45A4 → 54A7	45A4 → 53A6
backbone H-bonds (%)^d^	0.6	5.7***	5.2***	5.0***	4.5***	–0.5
native H-bonds (%)^d^	0.9	4.5***	6.8***	3.6***	6.0***	2.3**
polar SASA (%)^d^	0.6***	1.8***	–1.2***	1.2	–1.8***	–2.9***
nonpolar SASA (%)^d^	1.5	2.1	–2.4***	0.6	–4.0***	–4.6***
radius of gyration (%)^d^	0.2	0.8	∼0.0***^e^	0.5	–0.3***	–0.8***
α-helix (%)^d^	0.1	1.6***	0.3	1.5***	0.2	–1.2
β-strand (%)^d^	0.2	–1.4***	–1.2***	–1.5***	–1.3***	0.2
3_10_-helix (%)^d^	–0.1	0.5	0.4	0.6**	0.5	–0.1
*J*-coupling (Hz)	0.071	0.171	0.186	0.100	0.114	0.014
NOE violations (nm)	0.003	0.004	0.001	0.001	–0.002	–0.003
RMSD100^c^ (nm)	0.006	0.040***	0.019***	0.034***	0.013**	–0.021

a Positive values indicate an improvement in performance between parameter sets.

b The statistical significance was determined using transformed data as described in the text. It refers only to whether the results obtained using different parameter sets could be distinguished. Significance is denoted as follows: ***, *p* ≤ 0.001; **, 0.001 < *p* ≤ 0.01l *, 0.01 < *p* ≤ 0.05.

c RMSD normalized to 100 residues (only proteins with >40 residues were normalized).

d Differences in the percent error with respect to the experimentally derived quantity.

e The deviations from experiment are of the same magnitude with opposite signs.

The primary point to note in relation to Figures 3 and 4 as well as Table 3 is that the range of the values is large compared to the differences in the medians and means. There are also many outliers. As a result, observed differences in any one metric for any given protein have little (if any) significance when taken in isolation. Figures 3 and 4 also show little apparent correlation between the parameter sets for different metrics. The greatest loss of native hydrogen bonds is not obviously associated with the largest change in the radius of gyration or the greatest loss of a particular element of secondary structure. Thus, although a certain parameter set may appear to perform better with respect to a particular metric or a given protein may appear to perform better using a certain parameter set for a particular metric, comparisons based on individual metrics or involving a limited sample (i.e., a small set of proteins or few replicates) would appear to be of very little value when attempting to quantify the relative performance of different parameters sets.

The difficulty in identifying which parameter set performs best overall is also evident in Table 3. The first thing to note is that statistically significant differences in the means of the number of backbone hydrogen bonds, the number of native backbone hydrogen bonds, the polar and nonpolar SASA, and the radius of gyration could be readily observed despite short time scales examined. For example, it is clear that 54A8 and 54A7 yield similar results and that both are easily distinguished from 53A6 and 45A4. However, determining whether one of the parameter sets performs significantly better than another is much more challenging. For example, the only structural metric for which there was a significant difference between 54A8 and 54A7 is the polar SASA. As the primary difference between these parameter sets is the description of charged residues, this is not surprising. While statistically significant, the difference in the means was only 0.6%. This means the agreement between the polar SASA calculated from the simulations and that calculated from the corresponding experimental models was slightly better in the case of 54A8. Two points should be noted. First, a difference of 0.6% would be barely detectable in a single simulation. Second, X-ray structures might be expected to underestimate the polar SASA due to crystal packing effects. However, using either 54A8 or 54A7, the number of native backbone hydrogen bonds maintained during the simulations is significantly higher than when using 45A4 or 53A6. The difficulty is that in no case did the agreement with experiment improve for all metrics considered in the pairwise comparisons. For example, from Table 3 it can be seen that in going from 45A4 to 54A8, seven of the metrics examined showed a statistically significant difference regarding the agreement with experiment. Three showed an improvement, three showed a loss of agreement, and in one case the deviations from experiment are of the same magnitude with opposite signs, leading to no net change. Which parameter set might be judged to be better would depend on the weighting given to each of these individual metrics. Also, not all metrics improved with time: using either of the older parameter sets considered (45A4 or 53A6), the percentage of β-strand is much closer to the values obtained from the experimental structures than using any of the more recent parameter sets, suggesting that there are compensating effects.

It is possible that global properties based on the structure in the crystal and subject to crystal packing effects, such as the radius of gyration, might not be representative of the system free in solution and that local properties such as variations in elements of secondary structure are a better measure of whether the structure of the protein is maintained appropriately. Figure 4 shows boxplots of the difference in the percentage of residues assigned to the three main types of secondary structure observed in these structures based on the DSSP criteria. In the vast majority of cases, the deviation in the proportion of a given type of secondary structure compared to the experimental structures is less than 5%. Nevertheless, it has been suggested previously^30^ that the 53A6 parameter set leads to a higher proportion of β-strand than earlier or later parameter sets. However, the statistical significance of the observed differences between 53A6 and either 54A7 or 54A8 is marginal. In all cases the differences in the means are less than 2% (Table 3). The change in the percentage of residues assigned to α-helix and 3_10_-helix shows little dependence on the parameter set. This shows how the use of a limited test set can easily lead to bias.

### Distance Metrics

**Figure 5:**
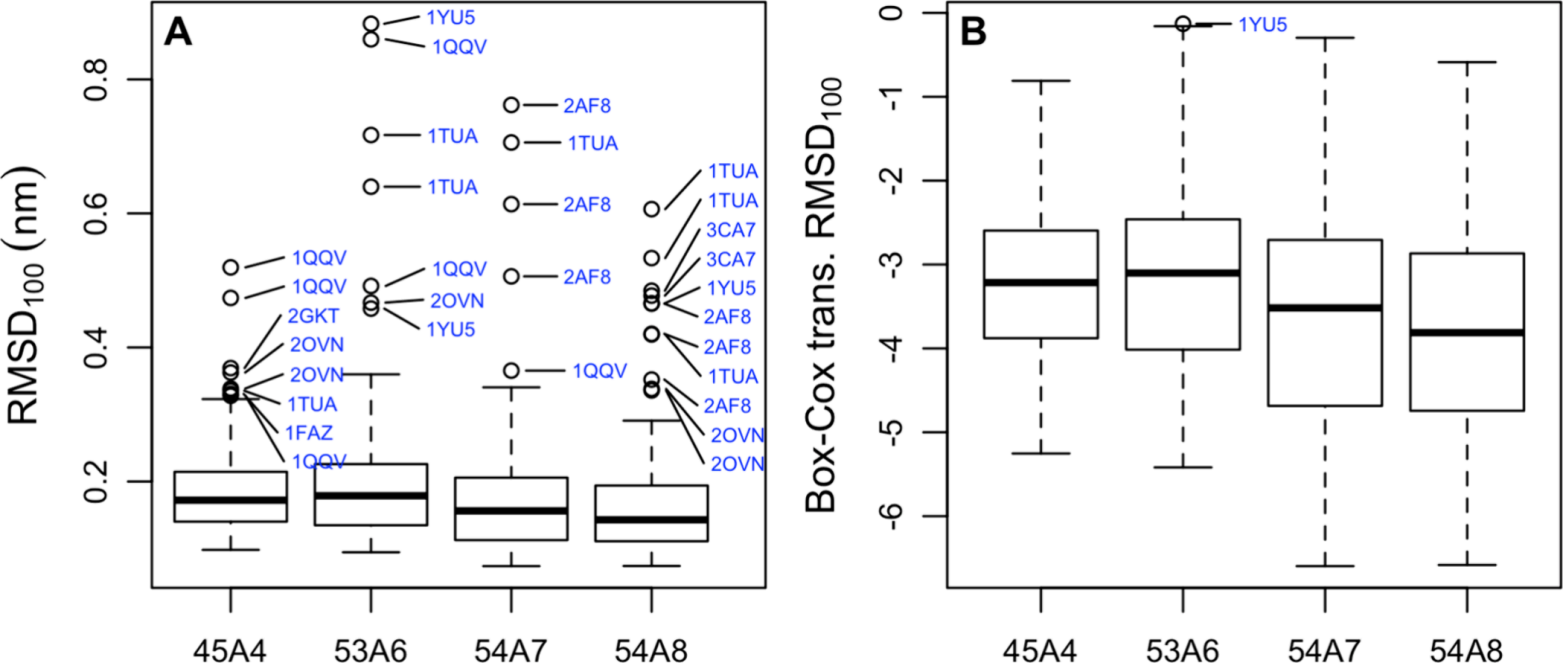
(A) Boxplots of the scaled positional root-mean-square deviation (RMSD_100_)^55^ between the backbone of conformations sampled during the last 5 ns of simulation and the corresponding starting conformation for a given parameter set. Note that the RMSD was not scaled for the three proteins that contained fewer than 40 residues (see the text). (B) The data in (A) after a Box-Cox transformation was performed.

The quality of a simulation is often judged in terms of a relative distance between a reference and the simulated structures. Most commonly this is done by calculating the positional RMSD from the starting configuration taken from the crystal or a given conformer in the NMR ensemble. The relative distance can also be quantified in terms of pairwise distances or dihedral angles. Each of these measures has limitations. For example, the positional RMSD is non-Euclidian and dependent on the size and shape of the molecule. While a change of 0.1 or 0.2 nm in positional RMSD may be highly significant in the case of a small globular protein, in the case of a large protein with an elongated structure it could be well within uncertainty. In an attempt to reduce the size and shape dependence and ensure that all proteins were given equal weighting, the backbone positional RMSD values were scaled to that of a 100-residue protein (RMSD_100_). Figure 5 shows a boxplot of the average of the RMSD_100_ between the starting experimental structure and conformations sampled over the last 5 ns of simulation. The median RMSD_100_ varies between a low of 0.15 nm in the case of 54A8 and a high of 0.19 nm in the case of 53A6. The 45A4 parameter set shows the least spread. However, the difference between the first and third quartile suggests that the differences observed are difficult to distinguish from noise. The mean values are provided in the Supporting Information (data file S4). Figure 5 also shows the same data after a Box-Cox transformation was performed to obtain data that are normally distributed. As indicted in Table 3, 54A8 did not differ significantly from 54A7, but 54A7 and 54A8 showed significant improvements over 53A6 and 45A4. Again, the effects were subtle, with the largest difference in the averages being only 0.04 nm between 54A8 and 53A6. There were also significant variations between individual proteins. The protein which showed the lowest RMSD_100_ differed for each parameter set. In fact, only one protein (4MHP) was among the lowest three proteins for more than one parameter set (i.e., second for 53A6 and third for 54A7). 4MHP is highly compact and the largest protein examined (Table 1 and Figure 1). The individual protein showing the largest RMSD_100_ also differed for each parameter set, although 1QQV, 1YU5, and 1TUA occurred multiple times in the top three. As can be seen from Figures 1 and 2, these three proteins are much less densely packed than others in the test set.

### Discrimination Based on Structural and Distance Metrics

When attempting to perform a validation study, it is important to determine whether any combination of the available criteria could be used to unambiguously distinguish between the alternative parameter sets given the time scale and range of proteins examined. As can be seen from Table 3, the results for the pairwise comparison are inconclusive, meaning that it is not possible to unambiguously rank the performance of the four parameter sets using the set of structural and distance-based metrics examined in this work.

This raises the question of whether the parameter sets are significantly different. In this case, we can treat this as a multivariate problem and combine the data from different metrics. For example, we can use BYMELRT to ask whether the choice of parameter set affects the outcome of the simulations. If we consider the RMSD_100_, the DSSP types α-helix, β-strand, β-bridge, and π-helix, the number of backbone hydrogen bonds, polar and nonpolar SASA, and radius of gyration for all 40 systems that were simulated with the four parameter sets simultaneously, the answer is clearly yes. In fact, the BYMELRT analysis suggests that the effect of the choice of parameter set on the combined outcomes from the simulations is highly significant (*p* < 1 × 10^–4^). Alternatively, we can use R’s *lme* function to generate a series of linear models to represent the available data. If we generate two sets of models that either include or exclude the choice of parameter set as a variable and then ask whether these two sets of linear models differ, again the result is positive, with the likelihood ratio test suggesting that the choice of parameter set has a significant effect on the results (*p* < 1 × 10^–4^). The analysis of the combined data clearly suggests that the alternative parameter sets lead to different outcomes. It is the determination of which parameter set performs best that is challenging. Based on the number of backbone hydrogen bonds and the adjusted positional RMSD_100_, the 54A8 parameter set would appear to be optimum. If properties such as the number of native backbone hydrogen bonds, polar and nonpolar SASA, radius of gyration, and DSSP secondary structure types are also included, the 54A7 parameter set has a better overall match to experiment. However, when the deviations from the target values for the structural and distance metrics were considered in combination, the results obtained using 54A7 and 54A8 did not differ significantly.

### NMR Observables

A potential criticism of the preceding analysis is that it is based on a comparison of single conformations and that the conformations of the proteins are primarily derived from crystallographic studies while the simulations yield an ensemble of conformations for a protein free in solution. In principle, one can directly compare the ensemble of conformations obtained in a simulation to NMR observables such as *J*-coupling constants, NOE intensities, or RDCs. However, while such NMR data are often used in both structure determination and to validate simulations, there are multiple challenges.^1,12^ First, *J*-coupling constants, NOE intensities, and RDCs are ensemble-averaged data, sensitive to motions on a millisecond time scale. Second, even if one ignores the effect of large-scale motions and structural heterogeneity, to directly calculate NOE intensities without assuming a motional model, one must compute spectral density functions. This requires simulation times well in excess of the rotational correlation time of the protein. Despite enormous advances in the time scales that can be accessed in simulations, a direct comparison to NMR observables is still only tractable for relatively small systems. Instead, derived data are used. *J*-coupling constants are related to dihedral angles using the Karplus curve and a given set of empirical parameters (as described in Methods) while comparisons to NOE intensity measurements are made based on distances derived assuming that the structure is spherical, rigid, and tumbling uniformly. RDCs avoid the need for a motional model or an empirical function such as the Karplus curve but instead require fitting of the orientation of the molecule and assumptions in regard to the number of distinct conformational states.

**Figure 6:**
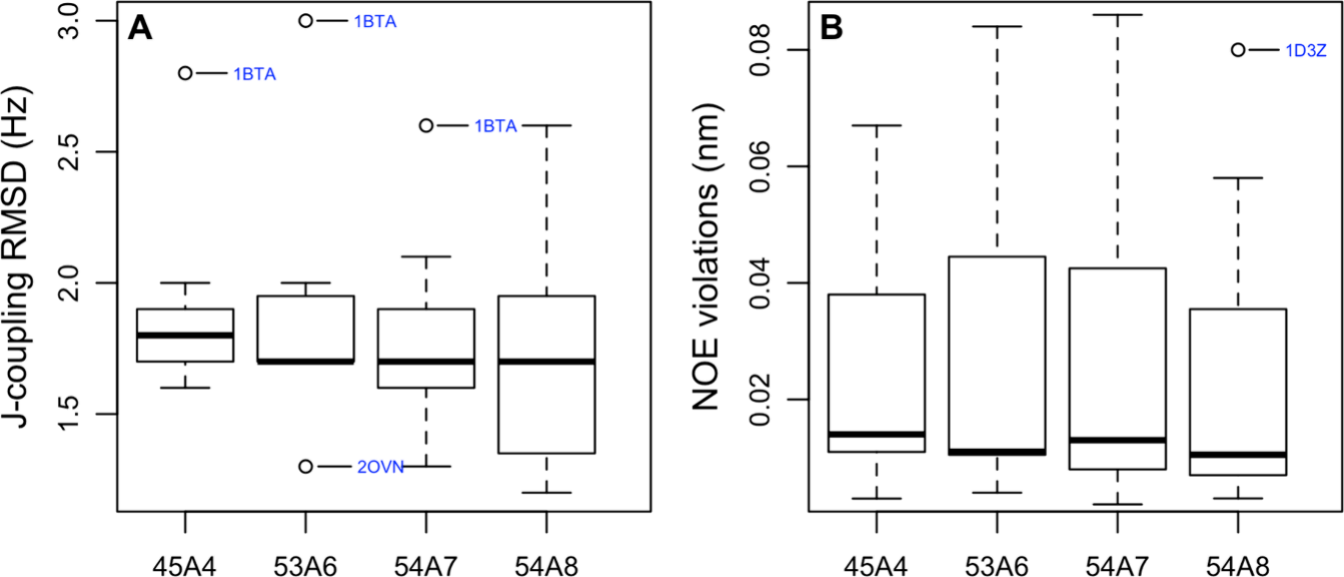
Boxplots showing (A) the RMSD between the *J*-coupling value measured experimentally and the *J*-coupling value calculated from the simulations configurations and (**B**) the average NOE violation. Only systems for which the starting conformation was derived based on NMR data and for which the corresponding experimental data were available were considered. The calculated values were based on conformations sampled during the last 5 ns of each simulation. Data from different replicas were pooled. The average NOE violation for each protein was obtained by summing the NOE distance violations and dividing by the total number of reported NOEs.

Of the 52 structures considered in this work, 13 were solved using NMR data. The structures of nine of the proteins were determined by both X-ray crystallography and NMR. Boxplots of the RMSD between the calculated *J*-value and experiment (*J*-coupling RMSD) for the protein backbone and the average NOE violation for the 13 structures solved using NMR data are shown in Figure 6. The average NOE violations were calculated by summing all NOE distance violations of the pooled replicates and dividing this by the total number of reported NOEs. It might be argued that the results obtained using 45A4 are the most consistent between proteins or that the 54A7 and 54A8 force fields perform slightly better. However, as indicated in Table 3, none of the differences between the parameter sets is statistically significant. This is due to the small number of proteins considered in this aspect of the study and the need to pool replicates. Which parameter set appears to perform best also depends on how the data are selected. For example, in the case of 1AFI, larger NOE violations were observed when using 54A7 or 54A8 compared to 53A6. However, the most significant NOE violations related to a single phenylalanine residue (Phe47). Inspection of the structure and the violations strongly suggests that the phenyl ring hydrogens HE1/2 and HD1/2 are wrongly assigned. Treating this assignment as ambiguous and applying pseudoatom corrections removed all NOE violations >0.05 nm in this structure. This would alter the statistics in favor of the more recent parameter sets. In contrast, 2OVN, a short helical peptide of just 17 residues, partly unfolds in replicates of 53A6 and 54A7 but not 54A8, suggesting that the results involving this peptide are statistically unreliable. Disregarding this case would remove much of the apparent difference between 54A8 and the other parameter sets by eliminating a case with high violations in 53A6 and 54A7 but low violations in 54A8.

### Backbone ϕ and ψ Dihedral Angles

**Figure 7:**
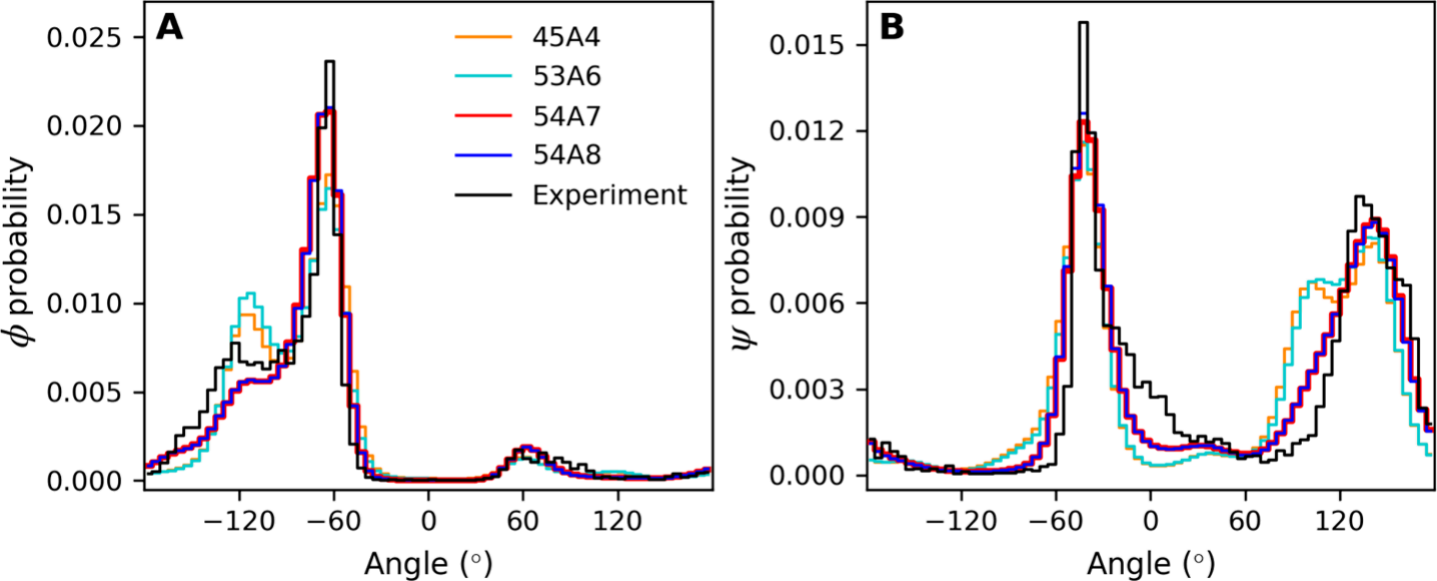
Normalized (A) ϕ and (B) ψ dihedral angle distributions for all proteins. The distributions were obtained by combining data from conformations sampled during the last 5 ns for all proteins simulated with a given parameter set. The equivalent plot obtained by combining data from each of the starting conformations is shown for comparison. The bin size was 5°. Note that the line width used for 54A7 is increased so that it can be distinguished from that of 54A8.

The backbone ϕ and ψ dihedral angles play a central role in determining the secondary and tertiary structure of a protein. Figure 7 shows the distributions of (A) ϕ and (B) ψ derived from conformations sampled during the last 5 ns of the simulations performed using each of the parameter sets for all systems. The dihedral distributions derived from the starting conformations are also shown for comparison.

In the case of the ϕ angles, the most pronounced differences between the parameter sets occur in the region between −140° and −100°. This region is over-represented compared to the X-ray-derived starting structures using 45A4 and 53A6 but under-represented using 54A7 and 54A8. Greater deviations between the distribution of angles observed in the simulations and the starting X-ray structures were found for ψ. For all four versions of the force field considered, angles between −100° and −50° and between 70° and 120° are over-represented in the simulations compared to the experiment, whereas angles between −30° and 30° are under-represented. The largest differences between the simulations and experiment are in the region between 70° and 120° using 45A4 or 53A6.

**Figure 8:**
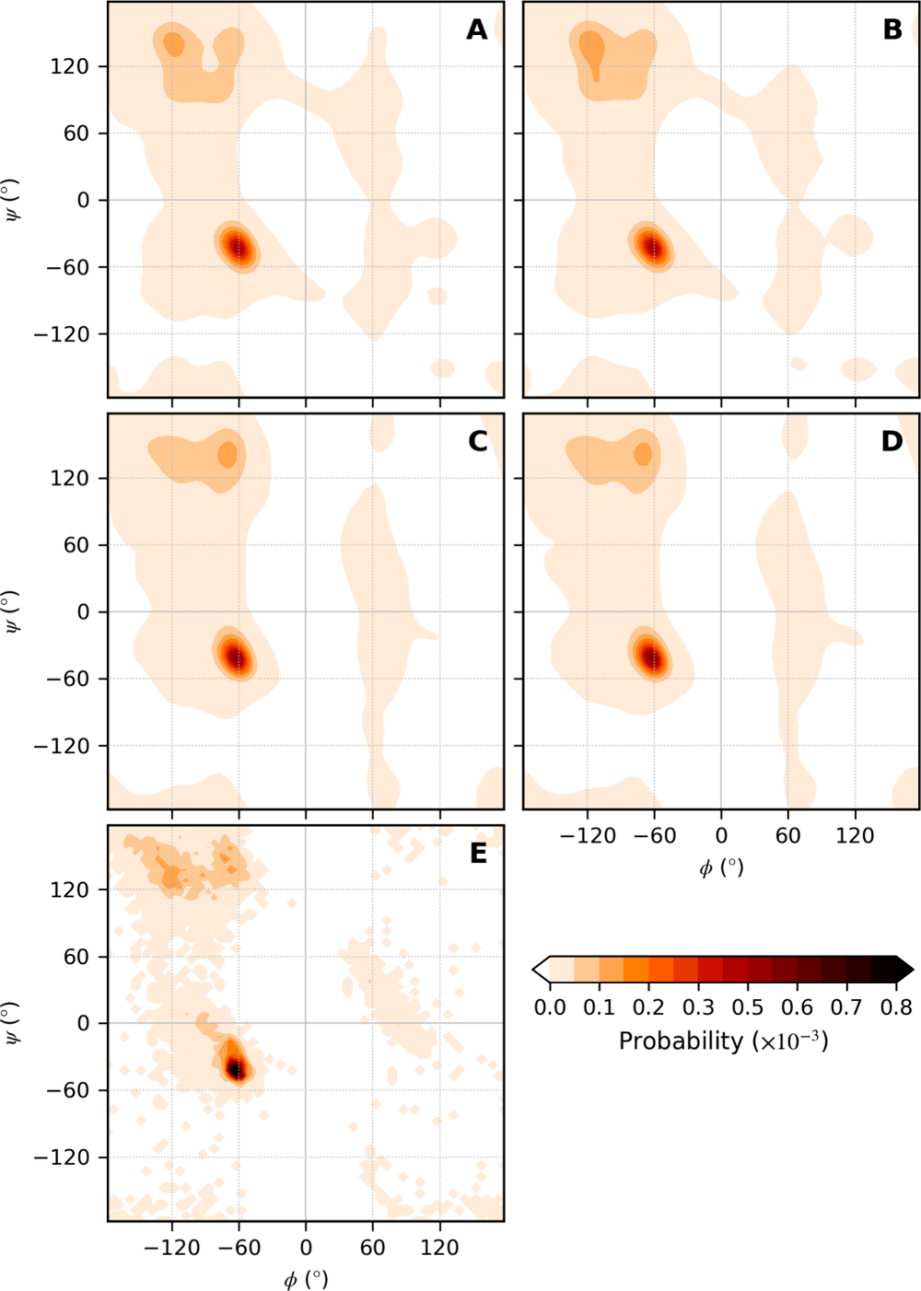
Ramachandran plots showing the relative probability of finding a given combination of ϕ and ψ angles for all structures simulated with the (A) 45A4, (B) 53A6, (C) 54A7, and (D) 54A8 parameter sets. The plots were constructed by combining all conformations sampled during the last 5 ns of the simulations using a bin size of 0.5°. (E) Equivalent plot for the starting conformations. Due to the more limited statistics in the case of the starting conformations, the probabilities were calculated using a bin size of 5°, resulting in the pixelated appearance.

While the histograms of the backbone ϕ and ψ angles clearly highlight the differences between the force fields, it is also useful to examine the relationship between the backbone ϕ and ψ angles as captured in a Ramachandran plot. Figure 8 shows Ramachandran plots derived from conformations sampled during the last 5 ns of the simulations initiated from structural models based on X-ray crystallography. The equivalent plot derived from the starting conformations is presented in Figure 8E. Although the overall helical propensity using 53A6 is known to be less than when using 45A4, 54A7 or 54A8, the α-helical region around φ = −65° and ψ = −40° differs little between the parameter sets with all being quite similar to the starting structures. The main difference is that the center of the distribution in 45A4 and 53A6 is shifted by 5° in ϕ to be just below −60°. In contrast, there are marked differences between the parameter sets in the β-strand region from ϕ = −130° to −60° and ψ = 60° to 130°. In the starting configurations the angles sampled in these structures are evenly distributed between ϕ = −120° and −70° and the density falls rapidly below ψ = 120°. In 45A4 and 53A6 the density in this region is primarily centered at ϕ = −120° and extends to ψ = 90°. In contrast, using 54A7 and 54A8 the density is primarily centered at ϕ = −70°. The density decreases below ψ = 120° but less than in the starting configurations.

**Figure 9:**
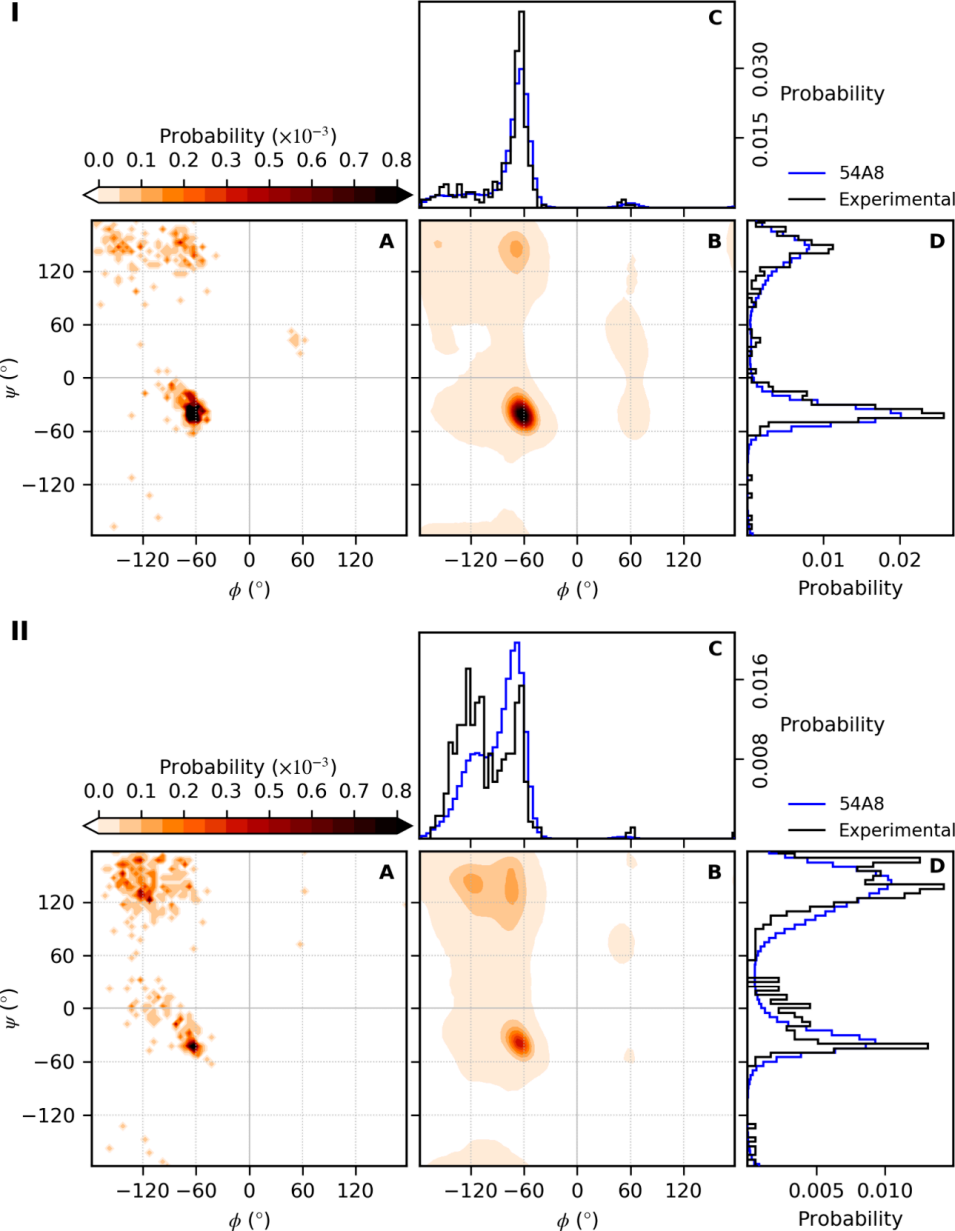
Example dihedral probability distributions for individual amino acids (alanine, panel I; threonine, panel II): (A) probability of finding a given combination of ϕ and ψ angles in the experimentally obtained starting conformations; (B) probability of finding a given combination of ϕ and ψ angles simulated with the 54A8 parameter set; (C) probability distributions of the ϕ dihedral angle; (D) probability distributions of the ψ dihedral angle. The plots were constructed from conformations sampled during the last 5 ns of the simulations as described in Figures 7 and 8. The total numbers of alanine and threonine residues included in this analysis are 357 and 351, respectively.

Many of the deviations result from individual amino acids. Figure 9 shows ϕ and ψ values for alanine and threonine using the 54A8 parameter set. Equivalent plots for all amino acids simulated with each of the four parameter sets are provided in the Supporting Information (data file S5). Alanine and threonine are common amino acids. Nevertheless, ϕ and ψ statistics even using 52 proteins are limited (∼350 ϕ and ψ angles). For alanine, the distributions from the simulations closely match the starting conformations. For threonine, there are marked differences, especially in the region ϕ = −140° to −60°. Indeed, the under-representation in the region ϕ = −150° to −100° for 54A7 and 54A8 evident in Figure 7B is primarily due to the C_β_-branched amino acids (isoleucine, valine, and threonine). Problems with these residues have been noted previously, and corrections have been proposed.^67,68^ The combined Ramachandran plots shown in Figure 8 also suggest an over-representation of ϕ and ψ combinations nominally associated with disorder or a left-handed helix with significant density in the region ψ = −60° to −120°. This stems in part from residues that form strong local interactions (aspartic acid, glutamic acid, asparagine, and histidine). The major contribution, however, is from glycine. In particular, the differences in the values around ψ = 0° in Figure 7B are primarily associated with glycine.^67^ Whether the under-representation of ψ = 0° values in the simulations is a limitation in how glycine is described in the force field or a reflection of averaging in the X-ray data is uncertain.

## Discussion

To validate a particular protein force field or demonstrate that a proposed change in parameters results in a fundamental improvement in predictive ability, it is not sufficient to simply consider whether simulations performed for a given set of proteins are compatible with a particular (experimental) observable. Instead, one must consider a range of factors. These include the extent of variation between simulation replicas, the extent to which the replicates are independent, the extent of variation between different proteins, the accuracy with which the (experimental) observable has been determined, the accuracy with which the (experimental) observable can be calculated from the simulations, the sensitivity of the property calculated to changes in the parameters used in the simulations, and whether the improvement in the ability to predict one property is associated with a degradation in the ability to predict other properties of interest. All these factors affect the statistical robustness of the results. This makes determining whether changes in a parameter set leads to a fundamental improvement in the ability to represent protein systems extremely difficult.

In this work, 52 systems (39 X-ray diffraction structures and 13 NMR structures) were simulated for 15 ns in triplicate starting from the same initial structure with the GROMOS11 software package^46^ using four alternative parameter sets (45A4,^39,69^ 53A6,^20^ 54A7,^30^ and 54A8^40^). A wide range of structural properties were calculated and compared to the available experimental data. Two questions were examined. First, were the differences between simulations performed using alternative parameter sets statistically significant? Second, did a particular parameter set lead to a better overall match with experiment?

Before discussing the statistical analysis, it is important to highlight the choice of reference structures. The structures were selected primarily based on quality and the ability to compare results from structures of the same protein obtained in different environments. They varied from 17 to 326 amino acids in length and contained a diversity of secondary structure from all α-helix to all β-strand as well as various combinations of α-helix, β-strand, and 3_10_-helix. Some structures were highly compact, others elongated. Some contained disulfide bridges, others not. The set also contained multiple examples of some more common folds. In short, the set was chosen to be both robust and representative of systems of interest.

The most striking finding of this work is evident from simple visual inspection of the boxplots presented in Figures 3, 4 and 5: the variation due to the different parameter sets is small compared with the variation between alternative proteins and even replicate simulations of a given protein. This is despite the relatively short simulation times and the fact that the replicates were initiated from the same structures. While on average the results obtained with one parameter set may be closer to experiment for a specific quantity than another, if one performed a single simulation of a novel structure, the expected uncertainty in the results would be much greater than any difference due to the choice of parameter set. This does not mean the results obtained using the different parameter sets were equivalent. If one considers each of the structural metrics examined in isolation, the differences in the averages due to the choice of parameter set were statistically significant in at least one pairwise comparison for the number of backbone hydrogen bonds, the number of native hydrogen bonds, polar and nonpolar SASA, radius of gyration, the prevalence of secondary structure elements, and the RMSD_100_. However, even though the alternative parameter sets yielded significantly different results, the differences in the averages (Table 3) are small, and it is not possible to state unequivocally which set shows better overall agreement with experiment.

The inability to demonstrate which parameter set agrees best with experiment is not simply due to limitations in the number of proteins and/or the length of the replicate simulations. It is also due to changes in the parameter sets having opposing effects. For example, in the case of 45A4 and 54A8 an increase in the relative agreement with experiment for the RMSD_100_ and the presence of specific backbone hydrogen bonds is offset by a decrease in the relative agreement for the SASA and the radius of gyration (Figures 3 and 5).

The results presented here might lead one to question whether the changes introduced in going from the 45A4 parameter set to the 54A8 parameter set of the GROMOS force field are important. The introduction of the 53A6 parameter set was associated with improvement in the ability to reproduce the partitioning behavior of analogues of amino acids between polar and nonpolar environments. The 54A7 and 54A8 parameter sets clearly reproduce the φ and ψ distributions observed in the experimental structures better than earlier versions. Both represent fundamental advances in the underlying model irrespective of whether the changes lead to fundamentally different outcomes in an individual simulation.

A key aim in compiling this work was to highlight the difficulty of determining the relative utility of a given force field or parameter set. Despite marked differences between the parameter sets in terms of partitioning behavior and the distributions of the backbone dihedrals, the differences in structural criteria (RMSD, hydrogen-bonding patterns, etc.) or the ability to reproduce NMR parameters are minor. Even combining the results from over 8 μs of simulation involving 52 protein structures varying in size and secondary structure composition, it is simply not possible to rigorously determine which parameter set (if any) provides a better overall match to experiment. In short, given the variation between replicate runs and the variation between proteins, any differences obtained when performing a single simulation (or even a set of simulations) using any of these four parameter sets would be within the expected statistical uncertainty. This draws into question the utility of many studies in the literature purporting to show the superiority of a particular variant of a force field based on comparisons of structural properties, especially those which compare results from just a handful of proteins.

Finally, it should be noted that this work, and indeed all similar studies, involve trade-offs between the number of parameter sets examined, the number and size of the individual systems, the number of replicates, and the length of each simulation. We focused on quantifying differences in specific metrics due to the choice of parameter set given the variation between systems and the variation between replicate simulations on time scales similar to those used in the initial development of the GROMOS force field. The length of the individual simulations and factors that affect the accuracy of the forces calculated using a given parameter set will also be important.^70−72^ The length of the individual replicates (15 ns) means only the local conformational space was explored. The simulations were too short to probe large-scale protein motions. Much longer simulation times (μs range or longer) would be required to achieve complete sampling of the conformational space accessible to even the smallest of the systems in this test set. We chose to perform short simulations of a large number of diverse proteins as opposed to performing long simulations on a small number of proteins in order to increase the number of independent samples. By including structural models of the same protein derived from NMR as well as X-ray crystallography and by performing replicate simulations using the same initial structure but alternative starting velocities, we have been able to highlight the variation in the results both between runs and between closely related but different initial structures. It is also important to note that performing short runs, as done here, restricts the conformational space sampled to be close to the starting configuration (NMR or X-ray model). This artificially reduces sampling noise and facilitates the comparison to quantities derived from the experimental models. For example, it allowed us to illustrate how rapidly specific properties diverged and the extent of variations between runs. Indeed, the variation between runs and systems suggests that it would be very easy to bias the results of this or other studies by varying the reference system and/or run length.

## Conclusions

The parametrization and validation of empirical force fields used for simulating proteins is challenging. While small differences in parameters can lead to large deviations in whole proteins, variations between proteins and between replicates make it difficult to obtain statistically meaningful results. This, together with uncertainties in how structural properties are characterized, means that the robustness of many force field validation studies is questionable. To help provide a framework for achieving statistical robustness, a test set of 52 protein systems (39 X-ray diffraction structures and 13 NMR structures) has been developed. To illustrate how this set might be used as part of a validation study, each protein was simulated for 15 ns in triplicate using four alternative GROMOS parameter sets. A wide range of structural metrics were examined, including changes in backbone hydrogen bonding, polar and nonpolar SASA, the radius of gyration, the occurrence of elements of secondary structure, and positional RMSD, as well as the ability to reproduce experimental NOE upper bound distances and *J*-coupling constants. Though the individual simulations were short, the combinations of a large number of proteins, multiple replicate simulations, and a diverse range of metrics meant that it could be readily shown that the results obtained using the alternative parameter sets differed significantly. However, the statistical scatter and variations between systems for different metrics meant it was not possible to determine, in a statistically rigorous manner, which parameter set best reproduced experiment, irrespective of which combination of metrics was used. That it was not possible to determine which parameter set was optimal despite combining results over 8.2 μs of simulation and multiple metrics should not be surprising. The times scales over which the structural properties of proteins vary are much longer than that sampled in this work. Additionally, the metrics commonly used to compare the structures sampled during a simulation to either models based on experimental data or the experimental observations themselves have multiple ambiguities. Indeed, the aim of this study was not to prove the superiority of a given parameter set or to promote the use of a particular set of structural metrics in validation studies. The aims were instead to highlight the challenges associated with the validation of protein force fields based on structural criteria and to establish a general framework for the testing and validation of protein force fields in a statistically robust manner for use in future studies by ourselves and others.

## Data Availability

To facilitate the use of this framework within the broader simulation community, all starting configurations, simulation input parameter files, and trajectories used in this analysis are available as part of the Australasian Computational and Simulation Commons (ACSC) Molecular Simulation Data Repository^73^ at https://molecular-dynamics.atb.uq.edu.au/collection/protein-force-field-validation-set. Furthermore, the code used for the statistical analysis and the relevant datafiles are available through the following GitHub repository: https://github.com/psykacek/rcode4ff.

## Supporting Information Available

Detailed description of the statistical analysis (PDF)
Lengths (in amino acids) of the individual proteins (XLSX)
Reference data for all properties analyzed (XLSX)
Simulation data for all properties analyzed (XLSX)
Individual ϕ and ψ distributions for all amino acids obtained using each parameter set (PDF)
