# Supplementary material for: On the Validation of Protein Force Fields Based on Structural Criteria
Source: J Phys Chem B. 2024 May 7;128(19):4602–20. doi: 10.1021/acs.jpcb.3c08469 (PMC11103706; doi:10.1021/acs.jpcb.3c08469)
Supplement: Supplementary file 1 — jp3c08469_si_001.pdf [file jp3c08469_si_001.pdf]

Supporting information for

On the Validation of Protein Force Fields Based on Structural Criteria

Martin Stroet[1*], Martina Setz[2*], Thomas Lee[1], Alpeshkumar K. Malde[3], Glen van den Bergen[1], Peter Sykacek[4], Chris Oostenbrink[2,5†] and Alan E. Mark[1†]

[1]The University of Queensland, St. Lucia, Queensland, 4072, Australia

[2]Institute for Molecular Modeling and Simulation, Department of Material Science and Process Engineering, University of Natural Resources and Life Sciences, Vienna, Muthgasse 18, 1190 Vienna, Austria

[3]Institute for Glycomics and School of Environment and Science, Griffith University, Gold Coast, Queensland, 4222, Australia.

[4]Institute of Computational Biology, Department of Biotechnology, University of Natural Resources and Life Sciences, Vienna, Muthgasse 18, 1190 Vienna, Austria

[5]Christian Doppler Laboratory for Molecular Informatics in the Biosciences, University of Natural Resources and Life Sciences, Vienna, Muthgasse 18, 1190 Vienna, Austria

* Martina Setz and Martin Stroet contributed equally to the work.

[†] Corresponding authors: chris.oostenbrink@boku.ac.at and a.e.mark@uq.edu.au

Data analysis supplement to "Challenges associated with the validation of protein force-fields based on structural criteria."

M. Stroet, M. Setz, T. Lee, A. Malde, G. van den Bergen,
P. Sykacek*, C. Oostenbrink and Alan E. Mark

March 28, 2024

1 Introduction

All calculations in this document are based on the statistics package R version 4.1.2 (2021-11-01). For improved reproducibility we provide the document source "ff_validation_nlme.Rnw" as supplement. When placed together with the files "Simulation_data.csv", "Reference_data.csv" and "pdb_id_lengths.csv" in the same directory, the document source can be processed by R and the package knitr [Xie, 2014, Xie, 2015], as long as all additional dependencies (availability of the R packages "kableExtra", "lme4", "emmeans", "car", "lattice", "nlme" and "dplyr") are met. To reproduce the pdf version of this supplement, interested readers have to use the following commands:

```
## in R:
library(knitr)
knit("ff_validation.Rnw")
## on the command line:
## > pdflatex ff_validation.tex
```

It should be kept in mind that optimization control in the scripts below were set to assure that a suitably small error tolerance is reached. In case you wish to replicate the analysis, you have to consider that as a result of these settings *the knitr phase in R takes a considerable amount of time.*

2 Characterization of simulated proteins

A detailed discussion of all metrics that were calculated from simulation outcome is provided in the main paper. From a data analysis perspective it is however important to describe the nature of the metrics as this is important for further statistical consideration.

Variable name	Term in paper	Type of metric
B_Strand:	β-strand	count metric
A_Helix:	α-helix	count metric
B_Bridge:	bridges between two β-strands in a β-sheet	count metric
ThreeTen_Helix:	3_{10}-helix	count metric
Pi_Helix:	π-helix	count metric
Hbond_bb_0.25_120:	Hydrogen bonds	count metric
SASA_polar:	solvent accessible surface area for polar residues	positive quantity
SASA_nonpolar:	solvent accessible surface area for non polar residues	positive quantity
Rgyr:	Radius of gyration	positive quantity
RMSD.ADJ:	Length adjusted positional RMSD	positive quantity
phi_rmsd:	Angular RMSD	positive quantity
psi_rmsd:	Angular RMSD	positive quantity
NOE_repl_merged:	NOE intensities	positive quantity
Jvalue:	J-coupling constants	positive quantity

*The responsibility for the analyses reported in this supplement lies with P. Sykacek email: peter.sykacek@boku.ac.at

3 Data preprocessing

Our approach for assessing protein characteristics follows [Villa et al., 2007], who proposed analyzing the effects of force fields on molecular-simulation derived protein characteristics with a MANOVA. MANOVA type analyses have the advantage of increased power of detecting significance in case of *correlated* multivariate responses. MANOVA or multivariate linear models assume that the residuals are multivariate Gaussian distributions.

The metrics which characterize proteins are however either counts or positive quantities. Analysis of such data will likely result in residuals which deviate from Gaussian distributions, thus violating assumptions which are inherent to MANOVA and linear models. To improve compliance with Gaussian distributed residuals, we apply Box-Cox power transformations [Box and Cox, 1964] on all individual metrics before subjecting the data to a multivariate multilevel analysis. We used to this end the functions provided in the R package car [Fox and Weisberg, 2019]. To fulfill the constraint of the Box-Cox power transformation in the car package that values must be larger zero we set all zero or negative values before transformation to a value which equals 10% of the smallest non zero value. This preprocessing was motivated to allow for a multivariate analysis of all protein characteristics in combination.

An additional complication arises in this particular situation with RMSD values which are known to depend on protein size. In combination with the unbalanced nature of the simulation experiment a protein length could confound the algorithm effect which we wish to assess. To avoid any chances of being mislead, we apply therefore the normalization procedure of RMSD values that was proposed in [Carugo and Pongor, 2001], before subjecting the adjusted RMSD values to a Box-Cox transformation as well.

A significance analysis for pair wise differences of metric values between force fields is supplemented by analyses which assess differences between simulation derived and measured protein characteristics. With measured protein characteristics we refer to characteristics whcih are derived from experimentally determined crystal structure. The latter result allows for judgements of the influence of force fileds on the agreement between simulation and measurement.

4 MANOVA and multivariate multilevel analysis

The analysis in this work is inspired by [Villa et al., 2007], who proposed an analysis of the effects of force fields on molecular-simulation derived protein characteristics. Their original approach is based on MANOVA type analyses which have the advantage of increased power of detecting significance in case of *correlated* multivariate responses. Albeit straightforward to apply, a conventional MANOVA has in our situation several shortcomings.

1. MANOVA is only applicable to complete multivariate vectors of protein characteristics. Including missing information requires in such analyses additional steps like multiple imputations.

2. Using linear model terminology, our assessment of force fields require to consider three effects: a) the force field, b) the simulated protein and c) technical replication of simulation runs. Technical drop outs (e.g. problems of the compute infrastructure) or a deliberate reduction of the number of lengthy simulations runs will in general cause unbalanced designs. This renders fixed effects analyses as in [Villa et al., 2007] poorly specified, as unbalanced multi-effects models will in general lead to a dependency of p-values on the chosen type of sums of squares calculation (type I, II and III ANOVA).

3. An even more profound implication on our assessments of force fields results from the fact that the replication of simulation runs and the variation of simulated proteins must be considered as independent random effects. Fixed effects analyses have in such situations a general tendency to overestimate significance. Such situations should thus preferably be assessed with mixed effects models [Pinheiro and Bates, 2000].

For considering the multivariate multilevel aspect of the data, we suggest following [Snijders and Boskers, 2012] pages 282 ff. To implement their proposition in our setting, we rely on the R-nlme package [Pinheiro and Bates, 2000]. In the context of multivariate multilevel analysis, we can use a likelihood ratio test [Mood et al., 1984] to assess all metrics in combination for significant dependencies on different force fields. Using the notion in [Snijders and Boskers, 2012], chapter 16, we have to compare an "empty" model which expresses the derived values of all metrics by a metric effect and attributes all further variation to the random effects "protein" and "simulation run". The more complex alternative hypothesis considers "force field" and interactions between "force field" and "metric" as additional fixed effects. A subsequent assessment of the likelihood ratio of these two models provides the required p-value for assessing the molecular simulation derived protein characteristics for significant dependencies on "force field".

4.1 Multivariate multilevel analysis

After data preprocessing the proposed assessment of whether predicted protein characteristics depend significantly on "force field" may be obtained by a step by step translation of the R and nlme based implementation of the example in [Snijders and Boskers, 2012] chapter 16. The authors provide a respective sample script at `http://www.stats.ox.ac.uk/~snijders/ch16.r` for download.

4.1.1 Rearranging the multivariate input data

1. The first step in applying a multivariate multilevel analysis requires us to rearrange the multivariate data to obtain a univariate response variable "all.y" which holds the preprocessed metric values. To allow the identification of the metric which corresponds to the value we need to add a factor variable "quant.fact" which denotes the corresponding type of metric. To complete the description of the data, additional factors are required to identify the applied force field ("all.alg"), the simulated protein ("all.comp") and the simulation run ("all.rep"). All variables are constructed from the preprocessed protein characteristics and bound to an R data frame.

2. Missing protein characterizations which appear as missing values in the multivariate input vectors are subsequently removed by reducing the data to all complete cases.

3. To allow calculating the correlation structure by the nlme package, the final step in rearranging the data is to reorder the data by metric type "quant.fact", protein id "all.comp" and simulation run "all.rep".

4.1.2 The "empty" multivariate multilevel model

Using the mixed effects linear model lme from the R nlme package for a multivariate multilevel analysis requires us to specify four parts.

1. Function lme requires to specify the fixed effects term of the model equation separately. For the "empty" model we assume that metric values are independent of the force field. Hence only depending on "quant.fact" the fixed effects model equation is:

   ```
   all.y~-1+quant.fact
   ```

2. The second specification concerns the random effects contribution. Irrespective of how we structure the fixed effects formula, we have to identify the metric value as conditional on the random effect "protein". The second random effect "simulation run" which is nested within "protein" determines the residual variance of the model and requires no separate specification. The required random effect model formula is thus:

   ```
   ~ -1+quant.fact|all.comp
   ```

3. An important characteristic of MANOVA analyses is their ability to model multivariate residuals. In order to unlock this ability for the essentially univariate lme model, we need to use its "weights" parameter. By an appropriate parametrization we allow for heteroscedasticity thus obtaining relations similar to MANOVA analyses where each sequence characteristic gets its own variance component. In the context of lme, we achieve this by using the "VarIdent" variance function (see [Pinheiro and Bates, 2000], page 209) which allows for residual variances to differ across levels of a stratification variable. In our case we have to use "quant.fact" as stratification variable and parameterize the weights parameter of lme as:

   ```
   weights=varIdent(form=~1|quant.fact)
   ```

4. To arrive at a noise model which mimics the multivariate residuals of a MANOVA we have finally also got to consider the correlation structure between different "quant.fact" levels. This is achieved by using the "corr" parameter of lme and a parametrization by one of the "corStruct" classes in nlme. In order to obtain a MANOVA compatible correlation structure, we use the generic corSym class (see [Pinheiro and Bates, 2000], page 234) and parameterize it by a formula which regards the numeric representation of the factor variable "quant.fact" as conditional on the random effects "simulation run" which is nested within "protein". The respective corr parametrization is thus:

```
corr=corSymm(form=~as.numeric(quant.fact)|all.comp/all.rep)
```

4.1.3 Adding force field as regressor

Our assessment of whether different force fields lead to statistically significant variations of sequence characteristics rely on a likelihood ratio test. This is achieved by comparing the “empty” model with a more complex multivariate multilevel model, which uses the factor “all.alg” representing different force fields as additional regressor. The only difference between the “empty” model and this alternative explanation of sequence characteristics is a different fixed effects formula which in our case is:

```
all.y~quant.fact*all.alg
```

4.1.4 Likelihood ratio test

The likelihood ratio test (see [Pinheiro and Bates, 2000], page 83) which allows us to infer whether the sequence characteristics we predict from simulation runs depend significantly on chosen force fields requires two model fits which are passed as parameters to function anova. The latter function calculates the p-value of the likelihood ratio test and provides a textual summary of the model comparison. Note that for reasons of clarity, details like the necessary adjustments of optimization control have been left out in this code chunk.

```
## fit of the empty model
lme.fit.n <- lme(all.y~-1+quant.fact, random = rnd.frm,
                 weights=varIdent(form=~1|quant.fact),
                 corr=corSymm(form=~as.numeric(quant.fact)|all.comp/all.rep),
                 data=univ.analyse.data,
                 control=alg.ctrl, method='ML')
## fit of the alternative model
lme.fit.a <- lme(all.y~quant.fact*all.alg, random = rnd.frm,
                 weights=varIdent(form=~1|quant.fact),
                 corr=corSymm(form=~as.numeric(quant.fact)|all.comp/all.rep),
                 data=univ.analyse.data,
                 control=alg.ctrl, method='ML')

## likelihood ratio test and summary of fit
anova(lme.fit.n, lme.fit.a)
```

4.2 Metric and force field specific analyses

Having shown by multivariate analysis that force fields lead to significant variation in the assessment metrics we will now prepare a more detailed assessment. To gain insight about interactions between force fields and assessment metrics we will now switch to analyses of univariate metrics which were previously transformed to approximate Gaussian residuals. As mentioned above the nesting of replicate simulation runs within compounds require this analysis to be carried out with linear mixed effects models. By keeping the metrics separate, adjustments for metric dependent residual variances and correlations between metrics are not required. To considerably simplify analysis we switch to a univariate mixed effects analysis with the lme4 package [Bates et al., 2015] for modeling and the emsmeans package [Lenth et al., 2019] for assessing the binary comparisons between force fields. The planned univariate assessments consist of several significance tests. The p-values are thus adjusted for multiple testing using the R p.adjust function and the Benjamini & Yekutieli FDR approach [Benjamini and Yekutieli, 2001]. The code fragments below illustrate analysis of RMSDs. The result tables are obtained by looping such code over all metrics (not shown but available in the accompanying source file ff_validation.Rnw).

4.2.1 Metric specific null model with lme4

The null model considers only the random effects all.comp (proteins) and replicate simulation run which determines the within compound residuals.

```r
## the lme4 package for linear mixed modeling
library(lme4)
library(emmeans)
## we illustrate RMSD as an example
rw.sel <- univ.analyse.data$quant.fact=="RMSD.ADJ"
fit.n <- lmer(all.y~(1|all.comp), data=univ.analyse.data[rw.sel,])
```

4.2.2 Metric specific alternative model and ANOVA

The alternative more complex model considers force field as fixed effect. Applying the anova function to both fits contrasts the goodness of fit with the increased complexity by a likelihood ratio test. To allow us to correct for multiple testing, we have to extract the p-value from the returned object.

```r
fit.a <- lmer(all.y~all.alg+(1|all.comp), data= univ.analyse.data[rw.sel,])
res <- anova(fit.n, fit.a)
p.value <-  res[["Pr(>Chisq)"]][2]
```

If we may assess the improvement by the alternative model as statistically significant, a more detailed inspection of force field induced differences with pairwise comparisons makes sense.

4.2.3 Pairwise contrasts with the emmeans package

The emmeans package is the preferred package for assessments whether contrasts deviate significantly from zero. Since we are interested in assessing all pairwise contrasts between algorithms for significance, we may use the standard emmeans workflow. To control the overall false positive rate for multiple testing, the p-values of all comparisons are finally adjusted using the Benjamini & Yekutieli FDR approach as implemented in the standard R p.adjust function.

```r
## next step: analysis of pairwise comparisons we do not adjust
## as we do that for all pairwise comparisons together.
res <- emmeans(fit.a, list(pairwise ~ all.alg), adjust = "none")
## convert the emmeans result to a dataframe
pdtab <- as.data.frame(res$"pairwise differences of all.alg")
## extract comparisons between force fields as strings
all.pairs <- levels(pdtab$contrast)[pdtab$contrast]
## and the corresponding p-values
all.pmp <- pdtab$p.value
BY.adj <- p.adjust(all.pmp, method="BY")
```

5 Results

5.1 Summary statistics of raw data

```
##  Hbond_bb_0.25_120 Hbond_native_bb_0.25_120   SASA_polar      SASA_nonpolar
##  Min.   :  4.00    Min.   :  1.00            Min.   : 9.316   Min.   : 4.584
##  1st Qu.: 29.00    1st Qu.: 22.75            1st Qu.:25.584   1st Qu.: 7.588
##  Median : 45.00    Median : 34.50            Median :32.255   Median : 9.604
##  Mean   : 51.33    Mean   : 40.17            Mean   :36.121   Mean   :10.183
##  3rd Qu.: 67.00    3rd Qu.: 51.00            3rd Qu.:44.090   3rd Qu.:11.431
##  Max.   :169.00    Max.   :141.00            Max.   :96.359   Max.   :22.722
##
##       Rgyr           A_Helix          B_Strand     ThreeTen_Helix
##  Min.   :0.723   Min.   :  0.00   Min.   :  0.0   Min.   : 0.000
##  1st Qu.:1.102   1st Qu.:  5.00   1st Qu.:  8.0   1st Qu.: 2.000
```

```
##  Median :1.278   Median : 12.00   Median : 22.0   Median : 3.000
##  Mean   :1.297   Mean   : 23.68   Mean   : 25.9   Mean   : 3.107
##  3rd Qu.:1.434   3rd Qu.: 32.25   3rd Qu.: 37.5   3rd Qu.: 3.000
##  Max.   :2.077   Max.   :113.00   Max.   :123.0   Max.   :12.000
##
##      Jvalue     NOE_repl_merged     RMSD.ADJ          B_Bridge
##  Min.   :1.20   Min.   :0.0020   Min.   :0.07371   Min.   : 0.000
##  1st Qu.:1.70   1st Qu.:0.0090   1st Qu.:0.12072   1st Qu.: 1.000
##  Median :1.70   Median :0.0120   Median :0.16293   Median : 2.000
##  Mean   :1.83   Mean   :0.0253   Mean   :0.18371   Mean   : 2.761
##  3rd Qu.:2.00   3rd Qu.:0.0328   3rd Qu.:0.21013   3rd Qu.: 4.000
##  Max.   :3.00   Max.   :0.0860   Max.   :0.88296   Max.   :13.000
##  NA's   :506    NA's   :506
##     Pi_Helix
##  Min.   : 0.000
##  1st Qu.: 0.000
##  Median : 1.000
##  Mean   : 1.245
##  3rd Qu.: 2.000
##  Max.   :10.000
##
```

5.2 Box Cox transformed data

The panel of box plots below provides a visualization of the preprocessed sequence characteristics. Every panel shows five boxes which illustrate the distribution of a particular sequence characteristic for the force fields “45A4”, “53A6”, “54A7”, and “54A8”.

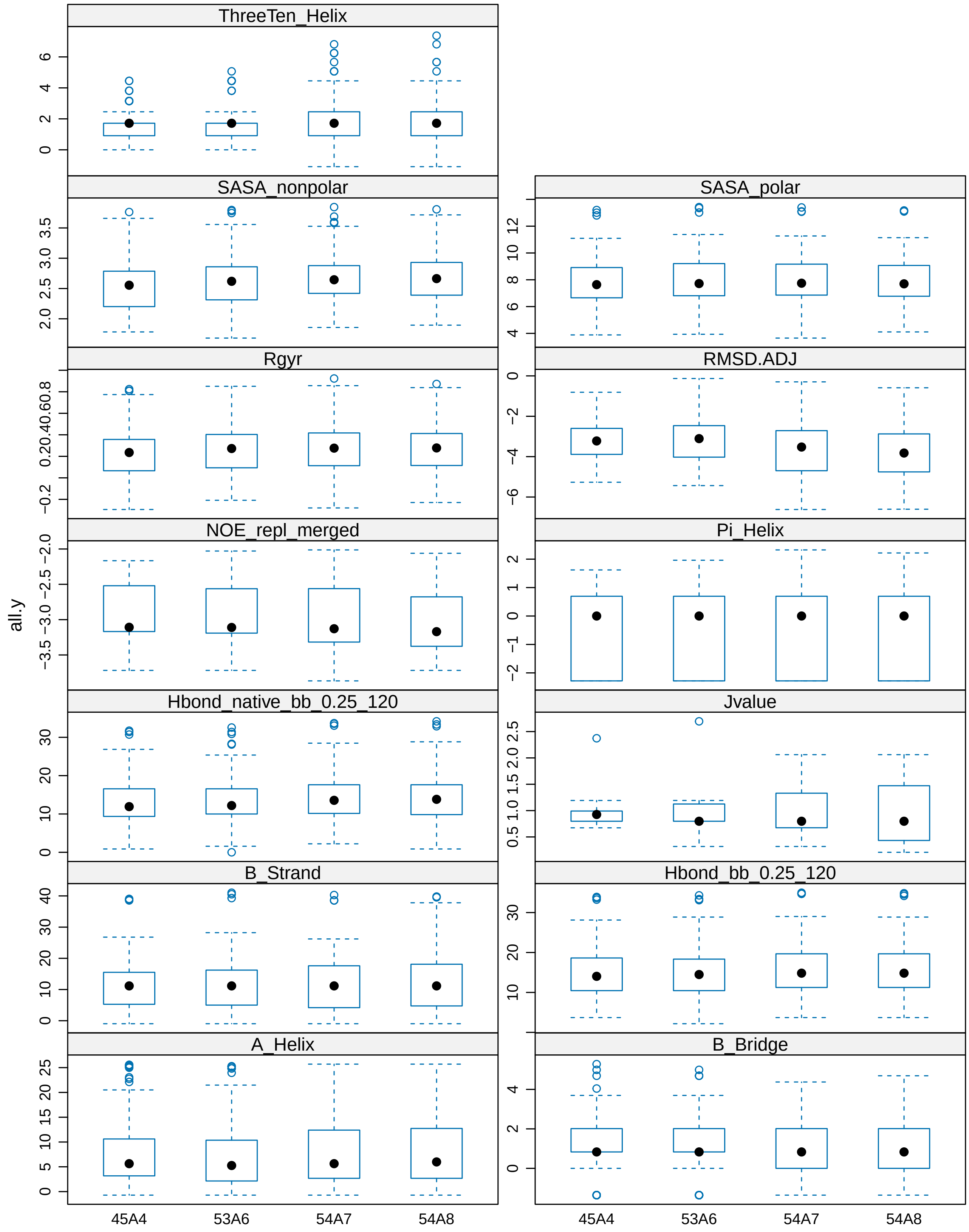

Figure S1. Distribution of structural properties for each protein force field.

5.3 MANOVA and multivariate multilevel analysis

```
##           Model  df      AIC      BIC    logLik   Test  L.Ratio p-value
## lme.fit.n     1 195 5768.429 7080.093 -2689.215
## lme.fit.a     2 234 5206.833 6780.830 -2369.417 1 vs 2 639.5958  <.0001
```

The ANOVA output compares the empty model against the more complex model which uses "all.alg" (representing force field) as regressor. A strongly increased likelihood and far superior AIC and BIC of the more complex model provide evidence that "force field" has a considerable effect on predicted sequence characteristics. Leading for the likelihood ratio test to a p-value of < 0.0001, the differences are of very high statistical significance. The p-value reported by the anova function follows common practice in statistics, where small p-values are usually only reported as "smaller than a threshold". The actual p-value from the likelihood ratio test is $\mathrm{p-val} = 1.088e-109$.

6 Property and algorithm specific mixed model analysis

After having concluded by a mixed effects regression analysis that the multivariate metric vector depends significantly on force field, we will now perform a more detailed analysis. We will to this end rely on two analyses which assess within metric.

1. by a likelihood ratio test whether adding force field as regressor leads to significant improvements over the empty (null) model.

2. by formulating all ten pairwise contrasts, subsequent significance analysis and multiple testing correction whether differences between two force fields are statistically significant.

The first stage of this fine grained analysis provides us thus with twelve p-values which result from the per metric likelihood ratio tests 1). The second stage of this analysis in 2) provides us across all metrics and pairwise contrasts with 80 p-values which assess whether the respective pair of force fields leads for a particular metric to significantly different expectations. To control the overall false positive rate, we adjust the p-values with the Benjamini & Yekutieli FDR (BY) for multiple testing.

```
## nr pairs: 78
## Order in all.pairs and all.rw.pairs match in:  0  % cases.
## Order in all.pairs and all.dyt.pairs match in:  100  % cases.
## Properties     : A_Helix B_Bridge B_Strand Hbond_bb_0.25_120 Hbond_native_bb_0.25_120 Jvalue NOE_repl_me
## Raw p-values   : 1.900596e-06 0.00622542 9.639084e-15 2.188943e-31 5.113798e-29 0.4009499 0.287061 0.234
## BH adjusted    : 3.088469e-06 0.008093046 2.506162e-14 1.422813e-30 2.215979e-28 0.4009499 0.3109828 0.2
## BY adjusted    : 9.821744e-06 0.02573697 7.96993e-14 4.524735e-30 7.04711e-28 1 0.9889668 0.8831639 1.15
```

6.1 Significance of metric specific likelihood ratio tests

By analyzing the results table we conclude that except for the Pi_Helix counts, Jvalue and NOE, all other metrics depend significantly on force field. The adjustment changes significance levels but does not change our assessment regarding significance.

6.2 Pairwise comparisons of force fields within metric

The rows in the subsequent table denote certain binary contrasts. The table columns contain information about the expected value of the contrast and the standard error (a 95% confidence interval). The Benjamini & Yekutieli adjusted significance levels assess whether the respective metric (transformed values as illustrated in the box plots)

Table S1: Significance of dependency of univariate metric values on force field

metric	raw.pval	BY.adj
A_Helix	1.90e-06 (***)	9.82e-06 (***)
B_Bridge	6.23e-03 (**)	2.57e-02 (*)
B_Strand	9.64e-15 (***)	7.97e-14 (***)
Hbond_bb_0.25_120	2.19e-31 (***)	4.52e-30 (***)
Hbond_native_bb_0.25_120	5.11e-29 (***)	7.05e-28 (***)
Jvalue	4.01e-01 ( )	1.00e+00 ( )
NOE_repl_merged	2.87e-01 ( )	9.89e-01 ( )
Pi_Helix	2.35e-01 ( )	8.83e-01 ( )
Rgyr	1.12e-25 (***)	1.16e-24 (***)
RMSD.ADJ	5.46e-11 (***)	3.22e-10 (***)
SASA_nonpolar	9.34e-13 (***)	6.44e-12 (***)
SASA_polar	1.12e-40 (***)	4.63e-39 (***)
ThreeTen_Helix	4.31e-03 (**)	1.98e-02 (*)

[a] metric: name of analysed metric; raw.pval: raw p-vale assessing dependencies of metric values on force field for significance; BY.adj: Benjamini & Yekutieli adjusted raw p-values.

Table S2: Binary contrasts between force fields[a]

	A_Helix	B_Bridge	B_Strand	Hbond_bb_0.25_120	Hbond_native_bb_0.25_120	Jvalue	NOE_repl_merged	Pi_Helix	Rgyr	RMSD.ADJ	SASA_nonpolar	SASA_polar	ThreeTen_Helix
45A4 - 53A6	0.36 (***)	0.09 ( )	-0.13 ( )	0.03 ( )	-0.31 (**)	0.02 ( )	-0.05 ( )	-0.06 ( )	-0.03 (***)	-0.07 ( )	-0.07 (***)	-0.19 (***)	0.08 ( )
45A4 - 54A7	0.00 ( )	0.16 (*)	0.46 (***)	-0.50 (***)	-0.75 (***)	0.11 ( )	-0.02 ( )	0.12 ( )	-0.02 (***)	0.32 (**)	-0.08 (***)	-0.16 (***)	-0.20 ( )
45A4 - 54A8	-0.07 ( )	0.18 (*)	0.45 (***)	-0.56 (***)	-0.89 (***)	0.13 ( )	0.03 ( )	0.16 ( )	-0.02 (***)	0.45 (***)	-0.07 (***)	-0.10 (***)	-0.10 ( )
53A6 - 54A7	-0.36 (***)	0.07 ( )	0.59 (***)	-0.53 (***)	-0.44 (***)	0.09 ( )	0.03 ( )	0.17 ( )	0.00 ( )	0.39 (***)	-0.00 ( )	0.02 ( )	-0.28 (**)
53A6 - 54A8	-0.43 (***)	0.09 ( )	0.58 (***)	-0.59 (***)	-0.58 (***)	0.11 ( )	0.08 ( )	0.21 ( )	0.00 ( )	0.52 (***)	0.00 ( )	0.09 (***)	-0.18 ( )
54A7 - 54A8	-0.07 ( )	0.02 ( )	-0.01 ( )	-0.07 ( )	-0.14 ( )	0.02 ( )	0.05 ( )	0.04 ( )	0.00 ( )	0.13 ( )	0.00 ( )	0.07 (***)	0.10 ( )

[a] Metrics are represented as expected value of the contrast ± standard error (95% confidence) and an indication of significance with (***) → p-value <0.001, (**) → p-value <0.01, (*) → p-value <0.05 and (.) → p-value <0.1. The p-values are adjusted for multiple testing using the Benjamini & Yekutieli FDR correction.

6.3 Pairwise assessments of simulation derived values

Table S3: Pairwise differences of protein characteristics between force fields. Metrics are represented as expected value of the contrast ± standard error (95% confidence) and an indication of significance with (***) → p-value < 0.001, (**) → p-value < 0.01, (*) → p-value < 0.05 and (.) → p-value < 0.1. Combinations of contrasts between force fields and metrics which we find significant are highlighted in blue.

Table S3: Binary contrasts of protein characteristics

	contrast	metric	value	rawval	significance
1	45A4 - 53A6	A_Helix	0.36 ± 0.09	1.26 ± 0.30	(***)
7	45A4 - 53A6	B_Bridge	0.09 ± 0.06	0.18 ± 0.09	( )
13	45A4 - 53A6	B_Strand	-0.13 ± 0.09	-0.43 ± 0.21	( )
19	45A4 - 53A6	Hbond_bb_0.25_120	0.03 ± 0.06	0.13 ± 0.29	( )
25	45A4 - 53A6	Hbond_native_bb_0.25_120	-0.31 ± 0.08	-1.26 ± 0.35	(**)
31	45A4 - 53A6	Jvalue	0.02 ± 0.09	0.03 ± 0.07	( )
37	45A4 - 53A6	NOE_repl_merged	-0.05 ± 0.05	-0.01 ± 0.00	( )
43	45A4 - 53A6	Pi_Helix	-0.06 ± 0.12	-0.09 ± 0.13	( )
49	45A4 - 53A6	Rgyr	-0.03 ± 0.00	-0.03 ± 0.00	(***)
55	45A4 - 53A6	RMSD.ADJ	-0.07 ± 0.09	-0.02 ± 0.01	( )
61	45A4 - 53A6	SASA_nonpolar	-0.07 ± 0.01	-0.54 ± 0.09	(***)
67	45A4 - 53A6	SASA_polar	-0.19 ± 0.01	-1.56 ± 0.12	(***)
73	45A4 - 53A6	ThreeTen_Helix	0.08 ± 0.08	0.10 ± 0.11	( )
2	45A4 - 54A7	A_Helix	0.00 ± 0.08	-0.42 ± 0.29	( )
8	45A4 - 54A7	B_Bridge	0.16 ± 0.06	0.20 ± 0.08	(*)
14	45A4 - 54A7	B_Strand	0.46 ± 0.08	0.94 ± 0.20	(***)
20	45A4 - 54A7	Hbond_bb_0.25_120	-0.50 ± 0.06	-2.46 ± 0.28	(***)
26	45A4 - 54A7	Hbond_native_bb_0.25_120	-0.75 ± 0.08	-3.12 ± 0.34	(***)
32	45A4 - 54A7	Jvalue	0.11 ± 0.09	0.09 ± 0.07	( )
38	45A4 - 54A7	NOE_repl_merged	-0.02 ± 0.05	-0.00 ± 0.00	( )
44	45A4 - 54A7	Pi_Helix	0.12 ± 0.11	0.13 ± 0.13	( )
50	45A4 - 54A7	Rgyr	-0.02 ± 0.00	-0.03 ± 0.00	(***)
56	45A4 - 54A7	RMSD.ADJ	0.32 ± 0.08	0.01 ± 0.01	(**)
62	45A4 - 54A7	SASA_nonpolar	-0.08 ± 0.01	-0.53 ± 0.09	(***)
68	45A4 - 54A7	SASA_polar	-0.16 ± 0.01	-1.35 ± 0.12	(***)
74	45A4 - 54A7	ThreeTen_Helix	-0.20 ± 0.08	-0.29 ± 0.11	( )
3	45A4 - 54A8	A_Helix	-0.07 ± 0.08	-0.67 ± 0.29	( )
9	45A4 - 54A8	B_Bridge	0.18 ± 0.06	0.22 ± 0.08	(*)
15	45A4 - 54A8	B_Strand	0.45 ± 0.08	0.88 ± 0.20	(***)
21	45A4 - 54A8	Hbond_bb_0.25_120	-0.56 ± 0.06	-2.75 ± 0.28	(***)
27	45A4 - 54A8	Hbond_native_bb_0.25_120	-0.89 ± 0.08	-3.66 ± 0.34	(***)
33	45A4 - 54A8	Jvalue	0.13 ± 0.09	0.11 ± 0.07	( )
39	45A4 - 54A8	NOE_repl_merged	0.03 ± 0.05	-0.00 ± 0.00	( )
45	45A4 - 54A8	Pi_Helix	0.16 ± 0.11	0.21 ± 0.13	( )
51	45A4 - 54A8	Rgyr	-0.02 ± 0.00	-0.02 ± 0.00	(***)
57	45A4 - 54A8	RMSD.ADJ	0.45 ± 0.08	0.02 ± 0.01	(***)
63	45A4 - 54A8	SASA_nonpolar	-0.07 ± 0.01	-0.50 ± 0.09	(***)
69	45A4 - 54A8	SASA_polar	-0.10 ± 0.01	-0.79 ± 0.12	(***)
75	45A4 - 54A8	ThreeTen_Helix	-0.10 ± 0.08	-0.17 ± 0.11	( )
4	53A6 - 54A7	A_Helix	-0.36 ± 0.08	-1.68 ± 0.29	(***)
10	53A6 - 54A7	B_Bridge	0.07 ± 0.06	0.01 ± 0.08	( )
16	53A6 - 54A7	B_Strand	0.59 ± 0.08	1.38 ± 0.20	(***)
22	53A6 - 54A7	Hbond_bb_0.25_120	-0.53 ± 0.06	-2.58 ± 0.28	(***)
28	53A6 - 54A7	Hbond_native_bb_0.25_120	-0.44 ± 0.08	-1.86 ± 0.34	(***)
34	53A6 - 54A7	Jvalue	0.09 ± 0.09	0.06 ± 0.07	( )

40	53A6 - 54A7	NOE_repl_merged	0.03 ± 0.04	0.00 ± 0.00	( )
46	53A6 - 54A7	Pi_Helix	0.17 ± 0.11	0.22 ± 0.13	( )
52	53A6 - 54A7	Rgyr	0.00 ± 0.00	0.00 ± 0.00	( )
58	53A6 - 54A7	RMSD.ADJ	0.39 ± 0.08	0.03 ± 0.01	(***)
64	53A6 - 54A7	SASA_nonpolar	-0.00 ± 0.01	0.01 ± 0.09	( )
70	53A6 - 54A7	SASA_polar	0.02 ± 0.01	0.22 ± 0.12	( )
76	53A6 - 54A7	ThreeTen_Helix	-0.28 ± 0.08	-0.39 ± 0.11	(**)
5	53A6 - 54A8	A_Helix	-0.43 ± 0.08	-1.94 ± 0.29	(***)
11	53A6 - 54A8	B_Bridge	0.09 ± 0.06	0.04 ± 0.08	( )
17	53A6 - 54A8	B_Strand	0.58 ± 0.08	1.31 ± 0.20	(***)
23	53A6 - 54A8	Hbond_bb_0.25_120	-0.59 ± 0.06	-2.88 ± 0.28	(***)
29	53A6 - 54A8	Hbond_native_bb_0.25_120	-0.58 ± 0.08	-2.40 ± 0.34	(***)
35	53A6 - 54A8	Jvalue	0.11 ± 0.09	0.08 ± 0.07	( )
41	53A6 - 54A8	NOE_repl_merged	0.08 ± 0.04	0.00 ± 0.00	( )
47	53A6 - 54A8	Pi_Helix	0.21 ± 0.11	0.30 ± 0.13	( )
53	53A6 - 54A8	Rgyr	0.00 ± 0.00	0.01 ± 0.00	( )
59	53A6 - 54A8	RMSD.ADJ	0.52 ± 0.08	0.04 ± 0.01	(***)
65	53A6 - 54A8	SASA_nonpolar	0.00 ± 0.01	0.04 ± 0.09	( )
71	53A6 - 54A8	SASA_polar	0.09 ± 0.01	0.78 ± 0.12	(***)
77	53A6 - 54A8	ThreeTen_Helix	-0.18 ± 0.08	-0.27 ± 0.11	( )
6	54A7 - 54A8	A_Helix	-0.07 ± 0.08	-0.26 ± 0.26	( )
12	54A7 - 54A8	B_Bridge	0.02 ± 0.05	0.02 ± 0.08	( )
18	54A7 - 54A8	B_Strand	-0.01 ± 0.08	-0.06 ± 0.18	( )
24	54A7 - 54A8	Hbond_bb_0.25_120	-0.07 ± 0.05	-0.29 ± 0.26	( )
30	54A7 - 54A8	Hbond_native_bb_0.25_120	-0.14 ± 0.07	-0.54 ± 0.30	( )
36	54A7 - 54A8	Jvalue	0.02 ± 0.09	0.03 ± 0.07	( )
42	54A7 - 54A8	NOE_repl_merged	0.05 ± 0.04	0.00 ± 0.00	( )
48	54A7 - 54A8	Pi_Helix	0.04 ± 0.10	0.08 ± 0.11	( )
54	54A7 - 54A8	Rgyr	0.00 ± 0.00	0.00 ± 0.00	( )
60	54A7 - 54A8	RMSD.ADJ	0.13 ± 0.07	0.01 ± 0.01	( )
66	54A7 - 54A8	SASA_nonpolar	0.00 ± 0.01	0.03 ± 0.08	( )
72	54A7 - 54A8	SASA_polar	0.07 ± 0.01	0.56 ± 0.10	(***)
78	54A7 - 54A8	ThreeTen_Helix	0.10 ± 0.07	0.12 ± 0.10	( )

6.4 Pairwise assessments of simulation derived differences from crystal structure derived truth

Table S4: Pairwise assessments of the discrepancies of simulation derived and experimentally validated protein characteristics. Differences between simulation derived and structure based protein characteristics are represented as expected value of the contrast ± standard error (95% confidence) and an indication of significance with (***) $\rightarrow$ p-value < 0.001, (**) $\rightarrow$ p-value < 0.01, (*) $\rightarrow$ p-value < 0.05 and (.) $\rightarrow$ p-value < 0.1. Combinations of contrasts between force fields and metrics which we find significant are highlighted in blue. To put these results into relation with table S1, we note that the results reported in table S2 will in general differ in p-vale and sign and absolute value of the expected contrast value from those in table S1. The exact same result will however be observed if the crystal derived "truth" is *smaller* than the simulation derived characteristics obtained with both force fields considered. The same p-value and an expected contrast with identical absolute value and alternating sign will be observed if the crystal derived "truth" is *larger* than the simulation derived characteristics obtained with both force fields considered.

A positive sign of the expected contrast value indicates that the force field which enters the contrast positively is closer to the crystal structure derived value. The alternating signs we observe in dependence of metric for a particular pair of force fields indicate that preference depends on the metrics considered. Unequivocal preferences for a particular force field can thus in general not be stated from these analyses.

Table S4: Binary contrasts of protein characteristic differences in simulation and measurement

	contrast	metric	value	significance
1	45A4 - 53A6	A_Helix	0.36 ± 0.09	(***)
7	45A4 - 53A6	B_Bridge	0.09 ± 0.06	( )
13	45A4 - 53A6	B_Strand	-0.13 ± 0.09	( )
19	45A4 - 53A6	Hbond_bb_0.25_120	0.03 ± 0.06	( )
25	45A4 - 53A6	Hbond_native_bb_0.25_120	-0.31 ± 0.08	(**)
31	45A4 - 53A6	Jvalue	0.02 ± 0.09	( )
37	45A4 - 53A6	NOE_repl_merged	-0.05 ± 0.05	( )
43	45A4 - 53A6	Pi_Helix	-0.06 ± 0.12	( )
49	45A4 - 53A6	Rgyr	-0.02 ± 0.00	(***)
55	45A4 - 53A6	RMSD.ADJ	-0.07 ± 0.09	( )
61	45A4 - 53A6	SASA_nonpolar	-0.06 ± 0.01	(***)
67	45A4 - 53A6	SASA_polar	-0.13 ± 0.02	(***)
73	45A4 - 53A6	ThreeTen_Helix	0.08 ± 0.08	( )
2	45A4 - 54A7	A_Helix	0.00 ± 0.08	( )
8	45A4 - 54A7	B_Bridge	0.16 ± 0.06	(*)
14	45A4 - 54A7	B_Strand	0.47 ± 0.08	(***)
20	45A4 - 54A7	Hbond_bb_0.25_120	-0.51 ± 0.06	(***)
26	45A4 - 54A7	Hbond_native_bb_0.25_120	-0.77 ± 0.08	(***)
32	45A4 - 54A7	Jvalue	0.12 ± 0.09	( )
38	45A4 - 54A7	NOE_repl_merged	-0.02 ± 0.05	( )
44	45A4 - 54A7	Pi_Helix	0.12 ± 0.11	( )
50	45A4 - 54A7	Rgyr	-0.02 ± 0.00	(***)
56	45A4 - 54A7	RMSD.ADJ	0.32 ± 0.08	(**)
62	45A4 - 54A7	SASA_nonpolar	-0.06 ± 0.01	(***)
68	45A4 - 54A7	SASA_polar	-0.11 ± 0.02	(***)
74	45A4 - 54A7	ThreeTen_Helix	-0.20 ± 0.08	( )
3	45A4 - 54A8	A_Helix	-0.07 ± 0.08	( )
9	45A4 - 54A8	B_Bridge	0.18 ± 0.06	(*)
15	45A4 - 54A8	B_Strand	0.45 ± 0.08	(***)
21	45A4 - 54A8	Hbond_bb_0.25_120	-0.57 ± 0.06	(***)
27	45A4 - 54A8	Hbond_native_bb_0.25_120	-0.90 ± 0.08	(***)
33	45A4 - 54A8	Jvalue	0.14 ± 0.09	( )
39	45A4 - 54A8	NOE_repl_merged	0.03 ± 0.05	( )
45	45A4 - 54A8	Pi_Helix	0.16 ± 0.11	( )
51	45A4 - 54A8	Rgyr	-0.02 ± 0.00	(***)
57	45A4 - 54A8	RMSD.ADJ	0.45 ± 0.08	(***)
63	45A4 - 54A8	SASA_nonpolar	-0.04 ± 0.01	(**)
69	45A4 - 54A8	SASA_polar	-0.08 ± 0.02	(***)
75	45A4 - 54A8	ThreeTen_Helix	-0.10 ± 0.08	( )
4	53A6 - 54A7	A_Helix	-0.36 ± 0.08	(***)
10	53A6 - 54A7	B_Bridge	0.07 ± 0.06	( )
16	53A6 - 54A7	B_Strand	0.60 ± 0.08	(***)
22	53A6 - 54A7	Hbond_bb_0.25_120	-0.54 ± 0.06	(***)
28	53A6 - 54A7	Hbond_native_bb_0.25_120	-0.46 ± 0.08	(***)
34	53A6 - 54A7	Jvalue	0.09 ± 0.09	( )
40	53A6 - 54A7	NOE_repl_merged	0.03 ± 0.04	( )
46	53A6 - 54A7	Pi_Helix	0.18 ± 0.11	( )
52	53A6 - 54A7	Rgyr	0.00 ± 0.00	( )
58	53A6 - 54A7	RMSD.ADJ	0.39 ± 0.08	(***)
64	53A6 - 54A7	SASA_nonpolar	-0.01 ± 0.01	( )
70	53A6 - 54A7	SASA_polar	0.02 ± 0.02	( )
76	53A6 - 54A7	ThreeTen_Helix	-0.28 ± 0.08	(**)
5	53A6 - 54A8	A_Helix	-0.43 ± 0.08	(***)

11	53A6 - 54A8	B_Bridge	0.09 ± 0.06	( )
17	53A6 - 54A8	B_Strand	0.58 ± 0.08	(***)
23	53A6 - 54A8	Hbond_bb_0.25_120	-0.60 ± 0.06	(***)
29	53A6 - 54A8	Hbond_native_bb_0.25_120	-0.59 ± 0.08	(***)
35	53A6 - 54A8	Jvalue	0.12 ± 0.09	( )
41	53A6 - 54A8	NOE_repl_merged	0.08 ± 0.04	( )
47	53A6 - 54A8	Pi_Helix	0.21 ± 0.11	( )
53	53A6 - 54A8	Rgyr	0.01 ± 0.00	( )
59	53A6 - 54A8	RMSD.ADJ	0.52 ± 0.08	(***)
65	53A6 - 54A8	SASA_nonpolar	0.01 ± 0.01	( )
71	53A6 - 54A8	SASA_polar	0.05 ± 0.02	(*)
77	53A6 - 54A8	ThreeTen_Helix	-0.18 ± 0.08	( )
6	54A7 - 54A8	A_Helix	-0.07 ± 0.08	( )
12	54A7 - 54A8	B_Bridge	0.02 ± 0.05	( )
18	54A7 - 54A8	B_Strand	-0.02 ± 0.08	( )
24	54A7 - 54A8	Hbond_bb_0.25_120	-0.06 ± 0.05	( )
30	54A7 - 54A8	Hbond_native_bb_0.25_120	-0.14 ± 0.07	( )
36	54A7 - 54A8	Jvalue	0.02 ± 0.09	( )
42	54A7 - 54A8	NOE_repl_merged	0.05 ± 0.04	( )
48	54A7 - 54A8	Pi_Helix	0.04 ± 0.10	( )
54	54A7 - 54A8	Rgyr	0.00 ± 0.00	( )
60	54A7 - 54A8	RMSD.ADJ	0.13 ± 0.07	( )
66	54A7 - 54A8	SASA_nonpolar	0.02 ± 0.01	( )
72	54A7 - 54A8	SASA_polar	0.03 ± 0.01	( )
78	54A7 - 54A8	ThreeTen_Helix	0.10 ± 0.07	( )

References

[Bates et al., 2015] Bates, D., Mächler, M., Bolker, B., and Walker, S. (2015). Fitting linear mixed-effects models using lme4. *Journal of Statistical Software*, 67(1):1–48.

[Benjamini and Yekutieli, 2001] Benjamini, Y. and Yekutieli, D. (2001). The control of the false discovery rate in multiple testing under dependency. *Ann. Statist.*, 29(4):1165–1188.

[Box and Cox, 1964] Box, G. E. P. and Cox, D. R. (1964). An analysis of transformations. *Journal of the Royal Statistical Society. Series B*, 26(2):211–252.

[Carugo and Pongor, 2001] Carugo, O. and Pongor, S. (2001). A normalized root-mean-square distance for comparing protein three-dimensional structures. *Protein Sci.*, 10(7):1470–1473.

[Fox and Weisberg, 2019] Fox, J. and Weisberg, S. (2019). *An R Companion to Applied Regression*. Sage, Thousand Oaks CA, third edition.

[Lenth et al., 2019] Lenth, R., Singmann, H., Love, J., Buerkner, P., and Herve, M. (2019). Estimated marginal means, aka least-squares means. Technical report, The University of Iowa. URL [https://CRAN.R-project.org/package=emmeans].

[Mood et al., 1984] Mood, A., Franklin, F. A., and Boes, D. C. (1984). *Introduction to the theory of statistics*. McGraw-Hill, Auckland, 3 edition.

[Pinheiro and Bates, 2000] Pinheiro, J. C. and Bates, D. M. (2000). *Mixed-Effects Models in S and S-PLUS*. Springer, New York.

[Snijders and Boskers, 2012] Snijders, T. A. B. and Boskers, R. J. (2012). *Multilevel Analysis: An Introduction to Basic and Advanced Multilevel Modeling*. Sage Publishers.

[Villa et al., 2007] Villa, A., Fan, H., Wassenaar, T., and Mark, A. E. (2007). How sensitive are nanosecond molecular dynamics simulations of proteins to changes in the force field? *J Phys Chem B*, 111(21):6015–6025.

[Xie, 2014] Xie, Y. (2014). knitr: A comprehensive tool for reproducible research in R. In Stodden, V., Leisch, F., and Peng, R. D., editors, *Implementing Reproducible Computational Research*. Chapman and Hall/CRC. ISBN 978-1466561595.

[Xie, 2015] Xie, Y. (2015). *Dynamic Documents with R and knitr*. Chapman and Hall/CRC, Boca Raton, Florida, 2nd edition. ISBN 978-1498716963.
