# Supplementary material for: On the Validation of Protein Force Fields Based on Structural Criteria
Source: J Phys Chem B. 2024 May 7;128(19):4602–20. doi: 10.1021/acs.jpcb.3c08469 (PMC11103706; doi:10.1021/acs.jpcb.3c08469)
Supplement: Supplementary file 5 — jp3c08469_si_005.pdf [file jp3c08469_si_005.pdf]

Supporting information for

On the Validation of Protein Force Fields Based on Structural Criteria

Martin Stroet[1*], Martina Setz[2*], Thomas Lee[1], Alpeshkumar K. Malde[3], Glen van den Bergen[1], Peter Sykacek[4], Chris Oostenbrink[2,5†] and Alan E. Mark[1†]

[1]The University of Queensland, St. Lucia, Queensland, 4072, Australia

[2]Institute for Molecular Modeling and Simulation, Department of Material Science and Process Engineering, University of Natural Resources and Life Sciences, Vienna, Muthgasse 18, 1190 Vienna, Austria

[3]Institute for Glycomics and School of Environment and Science, Griffith University, Gold Coast, Queensland, 4222, Australia.

[4]Institute of Computational Biology, Department of Biotechnology, University of Natural Resources and Life Sciences, Vienna, Muthgasse 18, 1190 Vienna, Austria

[5]Christian Doppler Laboratory for Molecular Informatics in the Biosciences, University of Natural Resources and Life Sciences, Vienna, Muthgasse 18, 1190 Vienna, Austria

* Martina Setz and Martin Stroet contributed equally to the work.

† Corresponding authors: chris.oostenbrink@boku.ac.at and a.e.mark@uq.edu.au

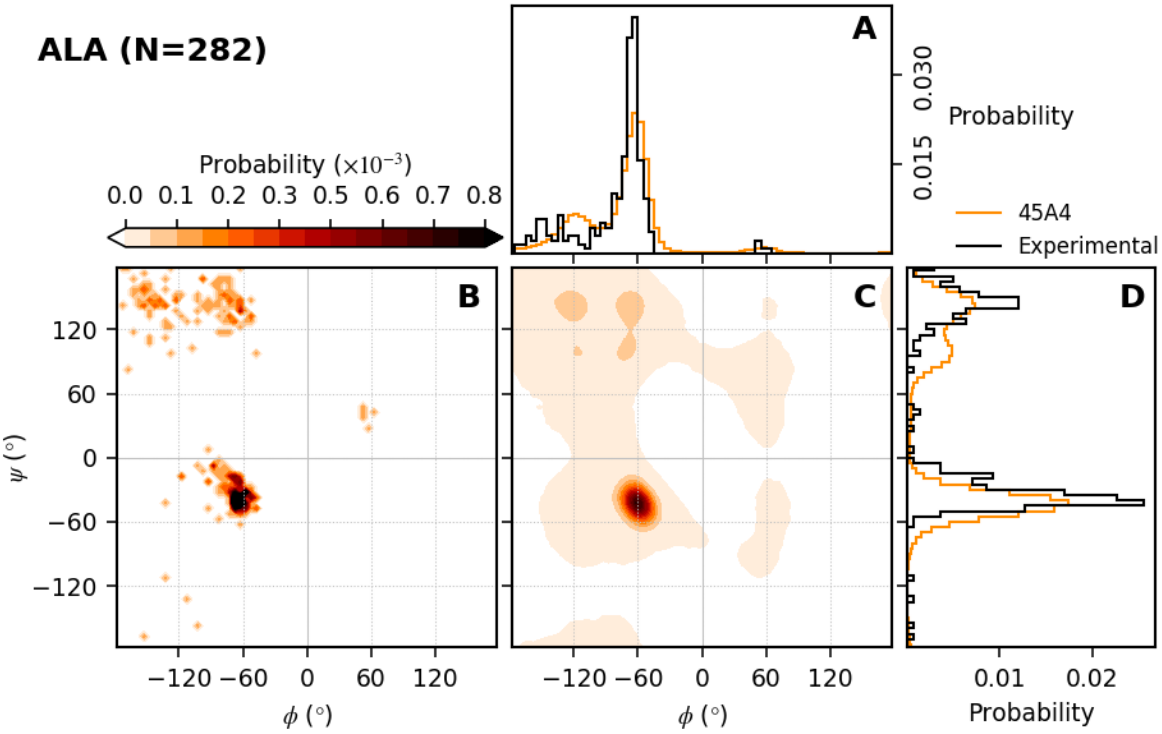
ALA (N=282)
Probability (×10⁻³)
0.0 0.1 0.2 0.3 0.5 0.6 0.7 0.8
A
B
C
D
0.015 0.030
Probability
45A4
Experimental
120
60
0
−60
−120
ψ (°)
−120 −60 0 60 120
ϕ (°)
0.01 0.02
Probability

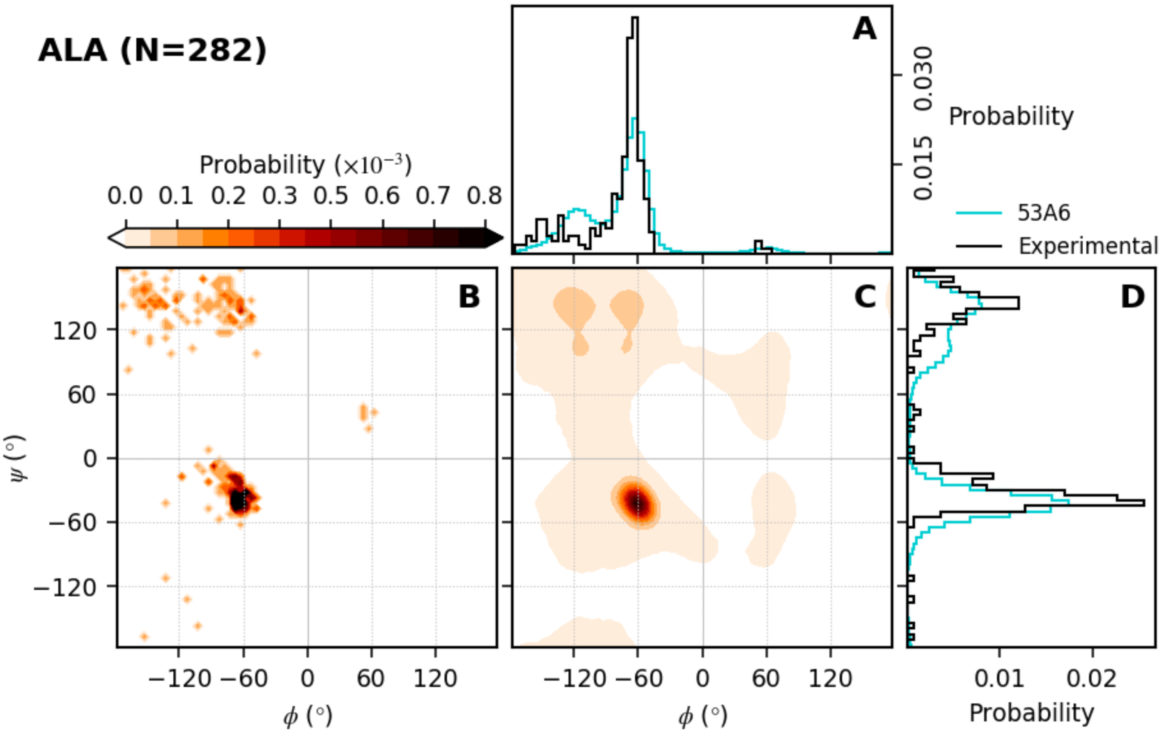
ALA (N=282)
Probability (×10⁻³)
0.0 0.1 0.2 0.3 0.5 0.6 0.7 0.8
A
B
C
D
0.015 0.030
Probability
53A6
Experimental
120
60
0
−60
−120
ψ (°)
−120 −60 0 60 120
ϕ (°)
0.01 0.02
Probability

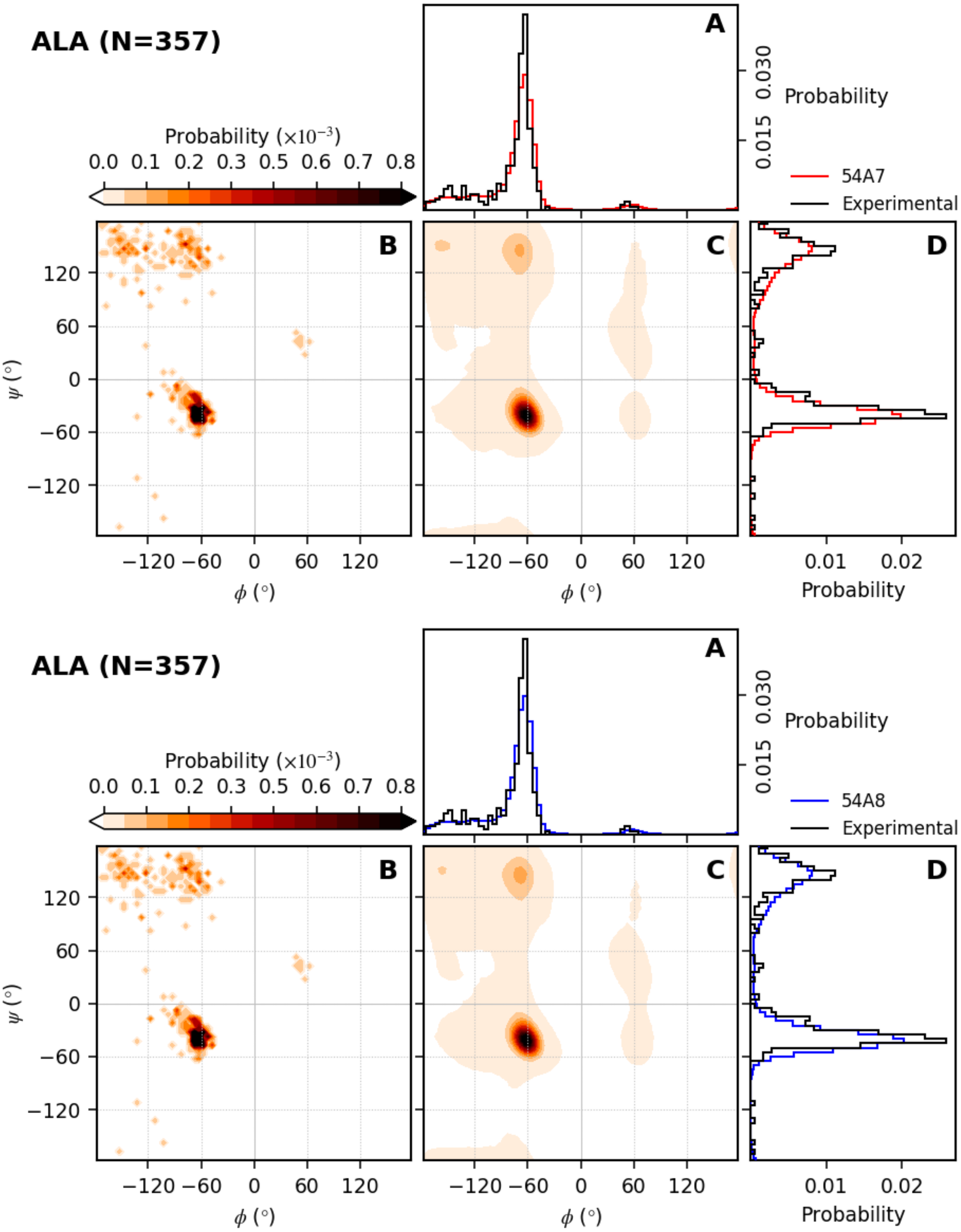

Figure S2. Distribution of alanine (ALA) backbone dihedral angles.

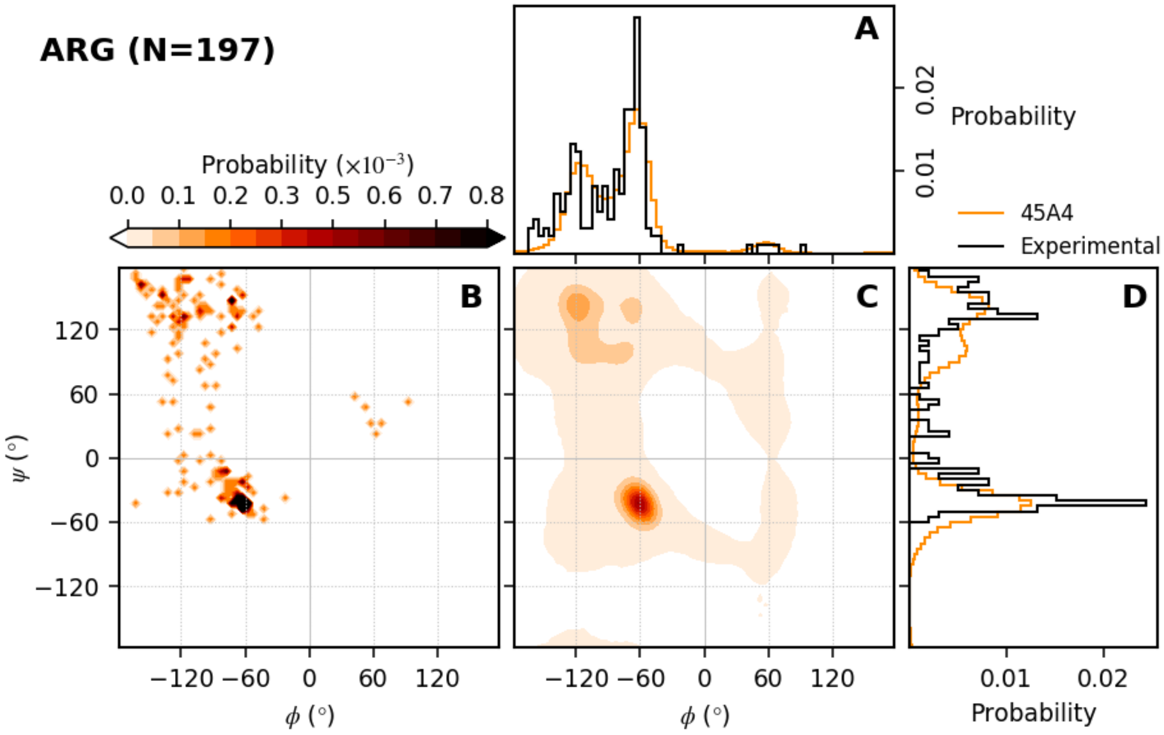
ARG (N=197)
Probability (×10⁻³)
0.0 0.1 0.2 0.3 0.5 0.6 0.7 0.8
A
B
C
D
Probability
45A4
Experimental
ψ (°)
ϕ (°)

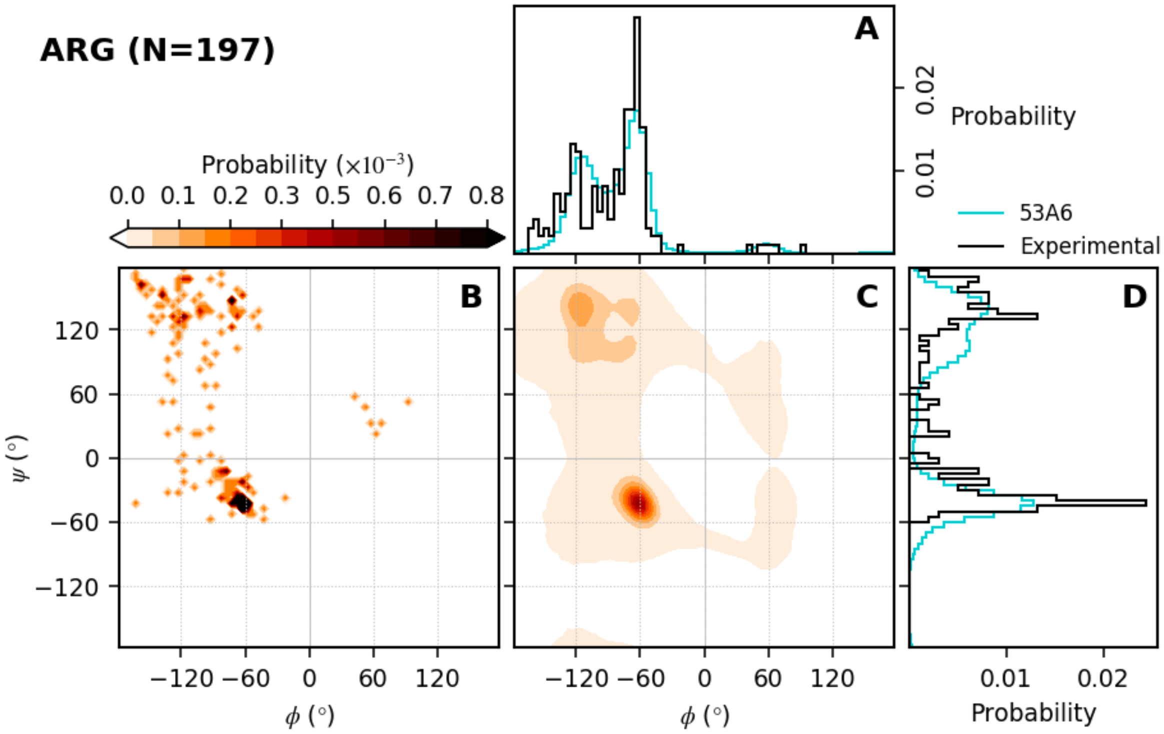
ARG (N=197)
Probability (×10⁻³)
0.0 0.1 0.2 0.3 0.5 0.6 0.7 0.8
A
B
C
D
Probability
53A6
Experimental
ψ (°)
ϕ (°)

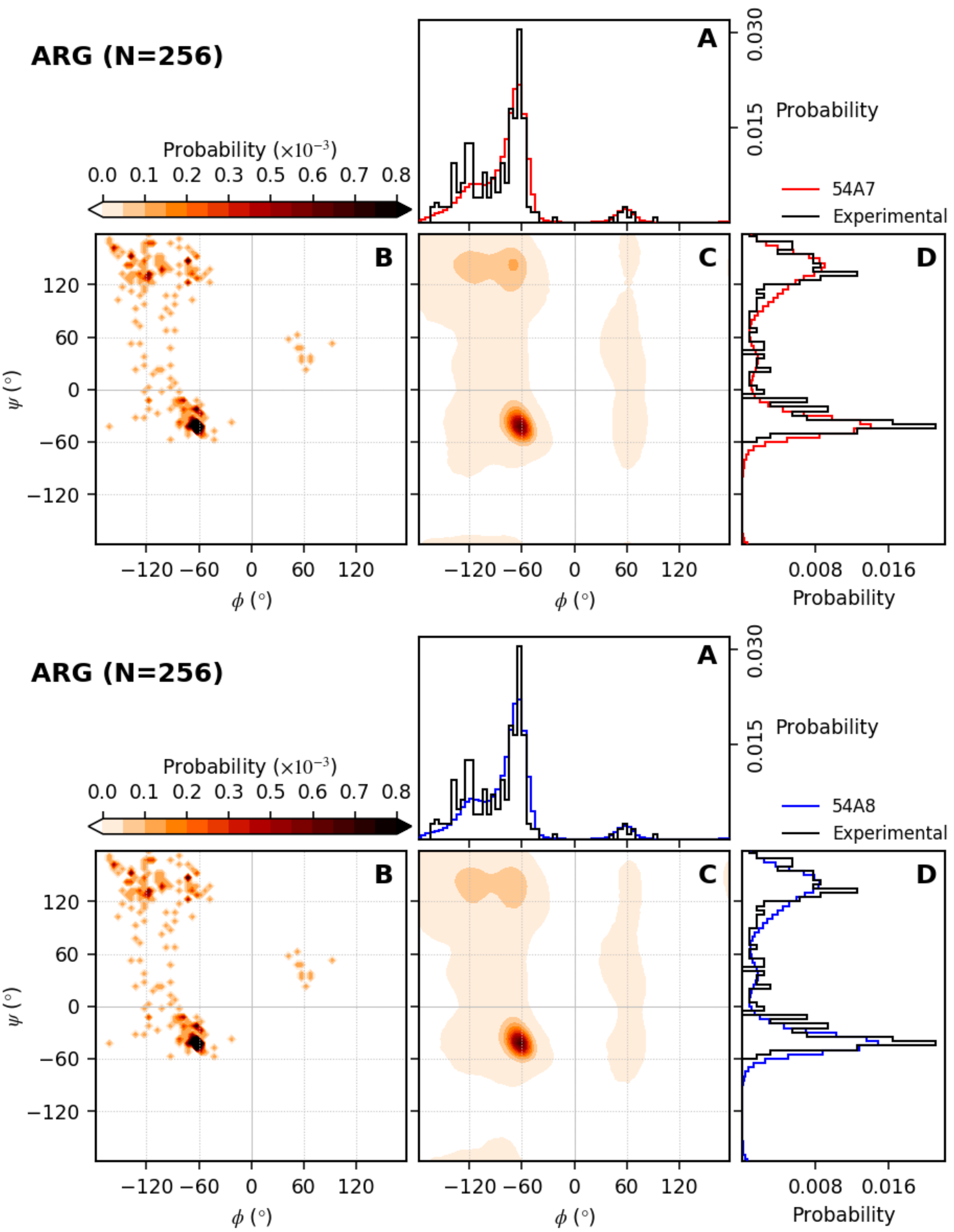

Figure S3. Distribution of arginine (ARG) backbone dihedral angles.

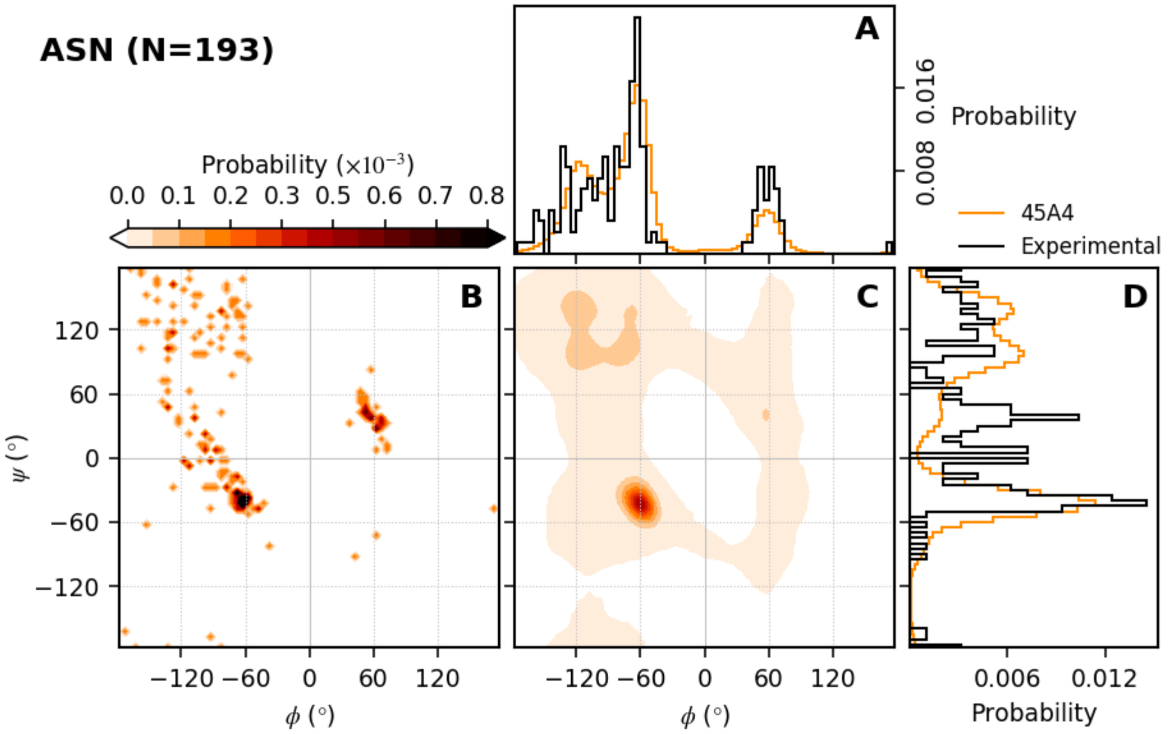
ASN (N=193)
Probability (×10⁻³)
0.0 0.1 0.2 0.3 0.5 0.6 0.7 0.8
A
B
C
D
Probability
45A4
Experimental
ψ (°)
φ (°)
Probability

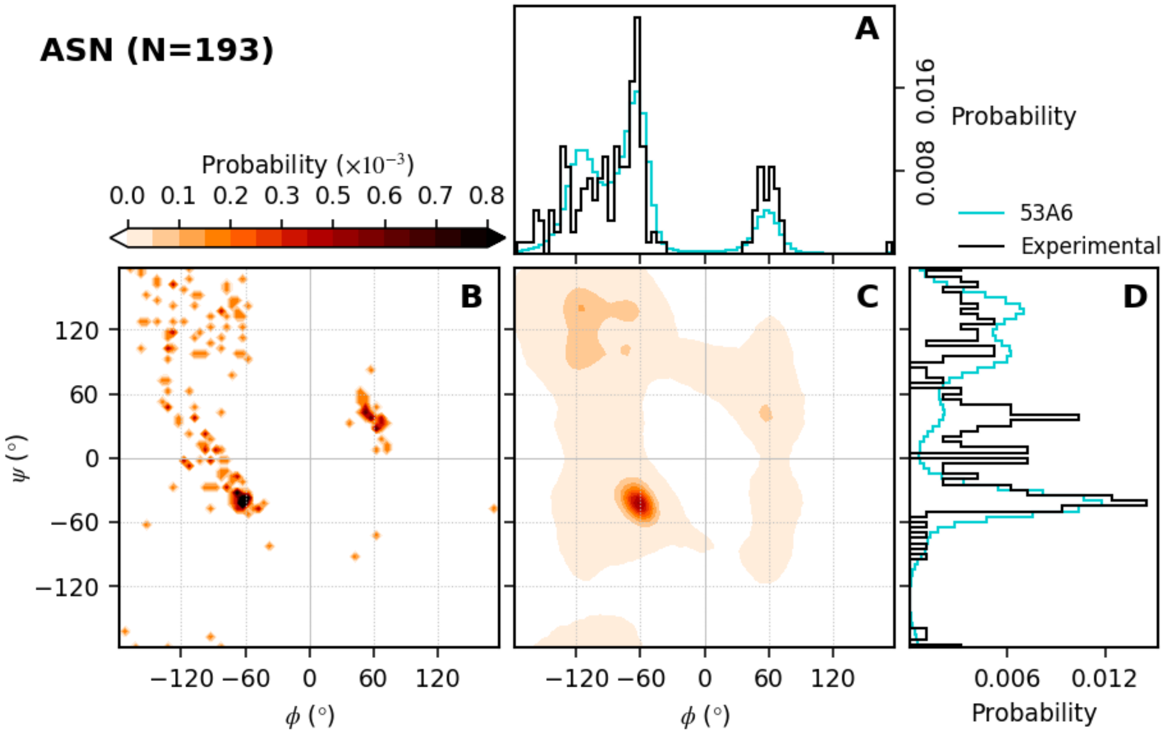
ASN (N=193)
Probability (×10⁻³)
0.0 0.1 0.2 0.3 0.5 0.6 0.7 0.8
A
B
C
D
Probability
53A6
Experimental
ψ (°)
φ (°)
Probability

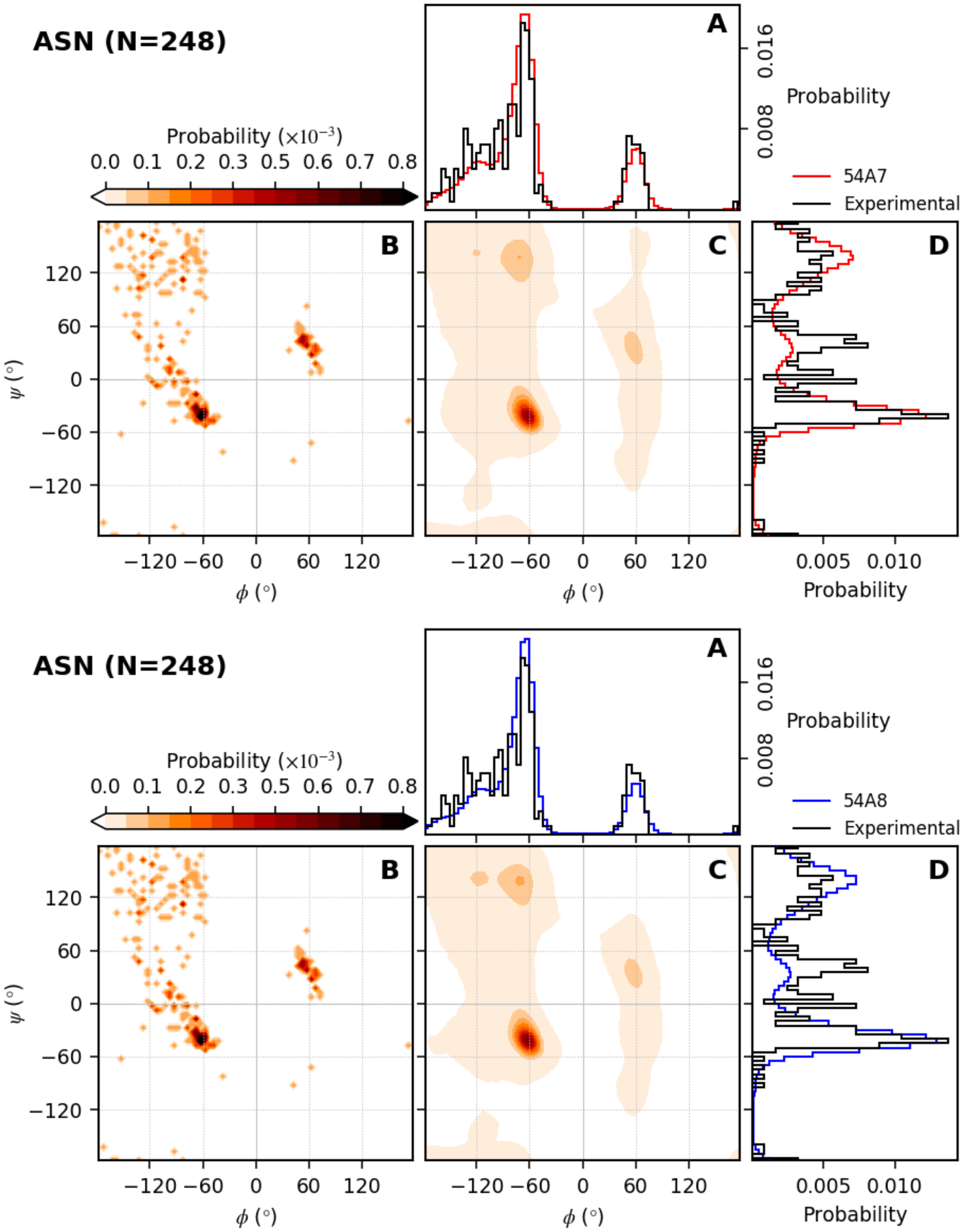

Figure S4. Distribution of asparagine (ASN) backbone dihedral angles.

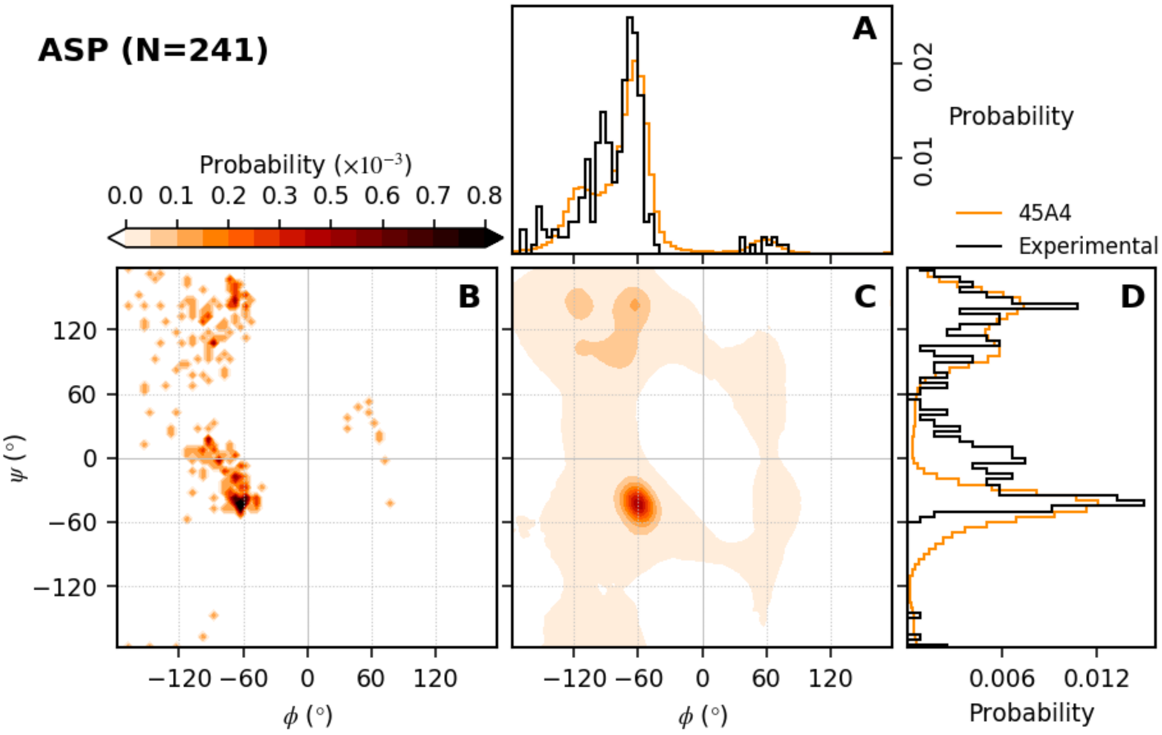

ASP (N=241)
A
B
C
D
Probability (×10⁻³)
0.0 0.1 0.2 0.3 0.5 0.6 0.7 0.8
Probability
0.01
0.02
45A4
Experimental
ψ (°)
φ (°)
−120 −60 0 60 120
0.006 0.012

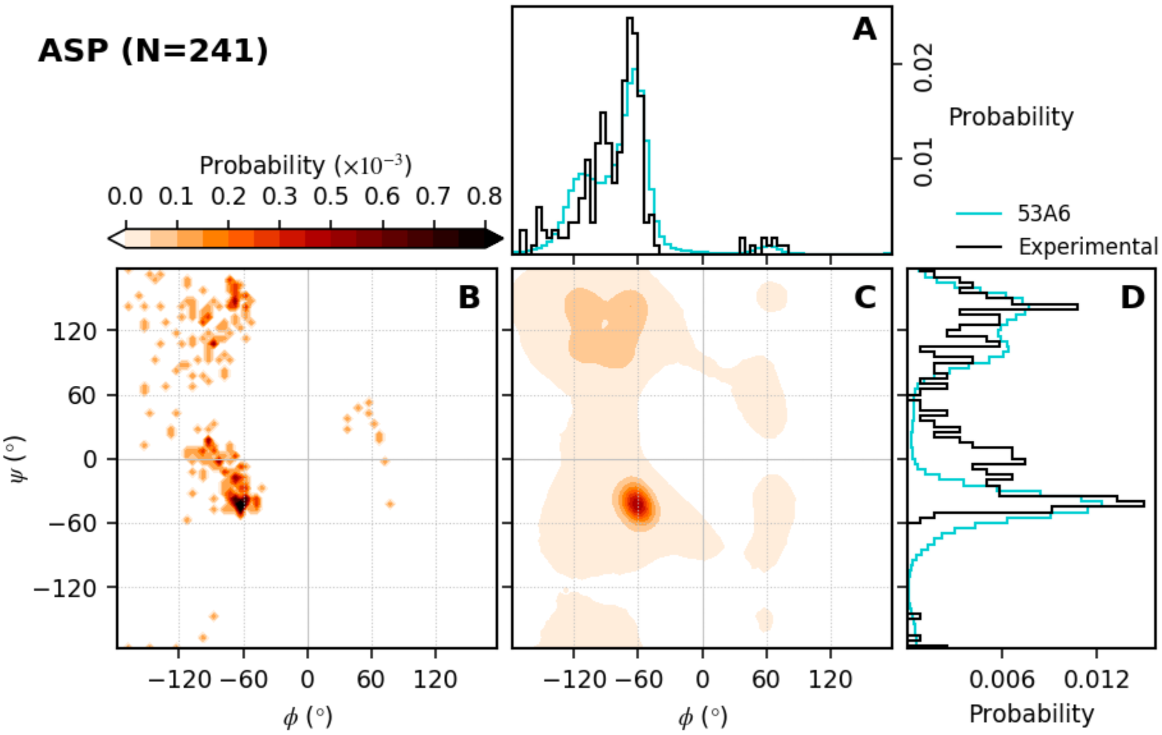

ASP (N=241)
A
B
C
D
Probability (×10⁻³)
0.0 0.1 0.2 0.3 0.5 0.6 0.7 0.8
Probability
0.01
0.02
53A6
Experimental
ψ (°)
φ (°)
−120 −60 0 60 120
0.006 0.012

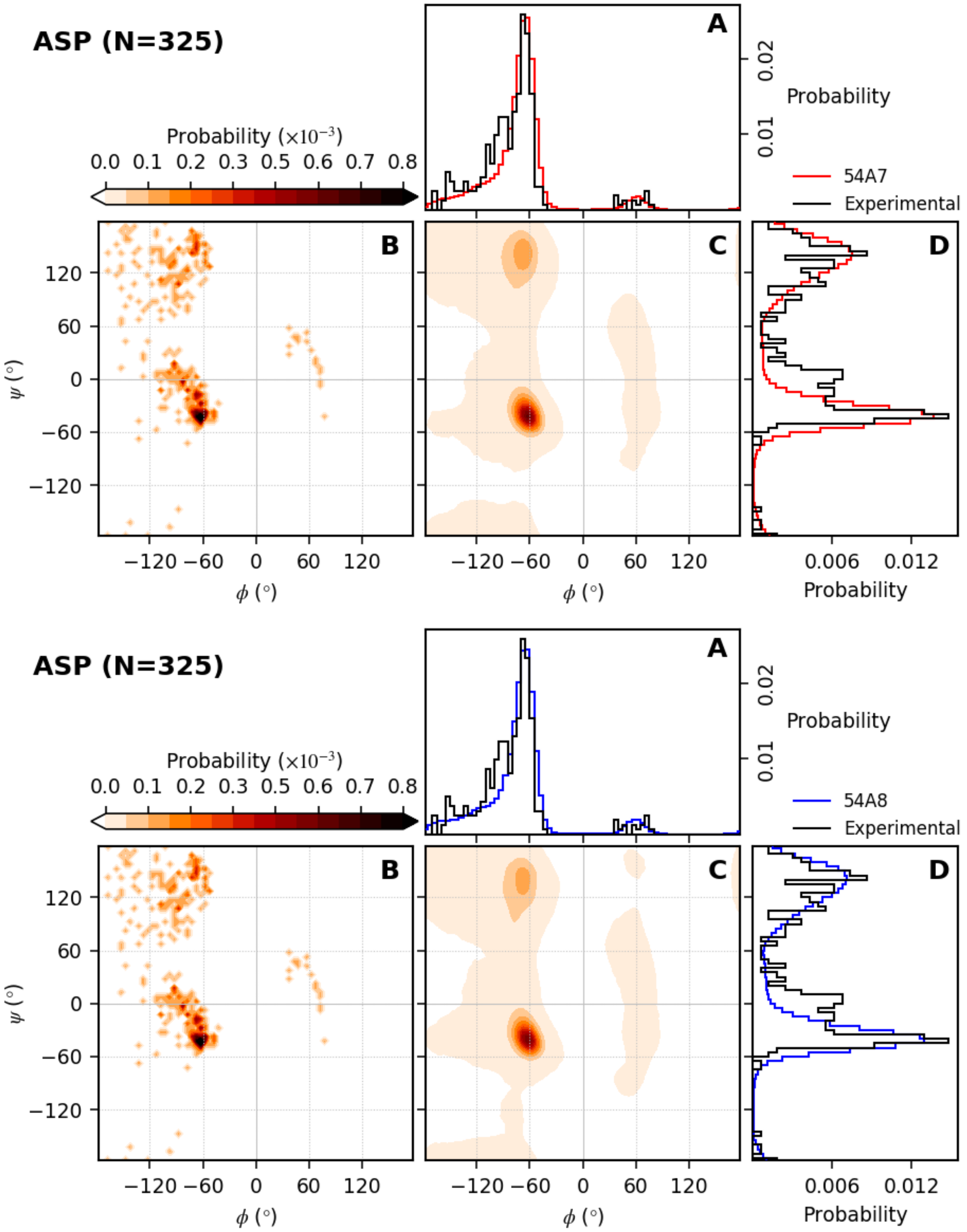

Figure S5. Distribution of aspartic acid (ASP) backbone dihedral angles.

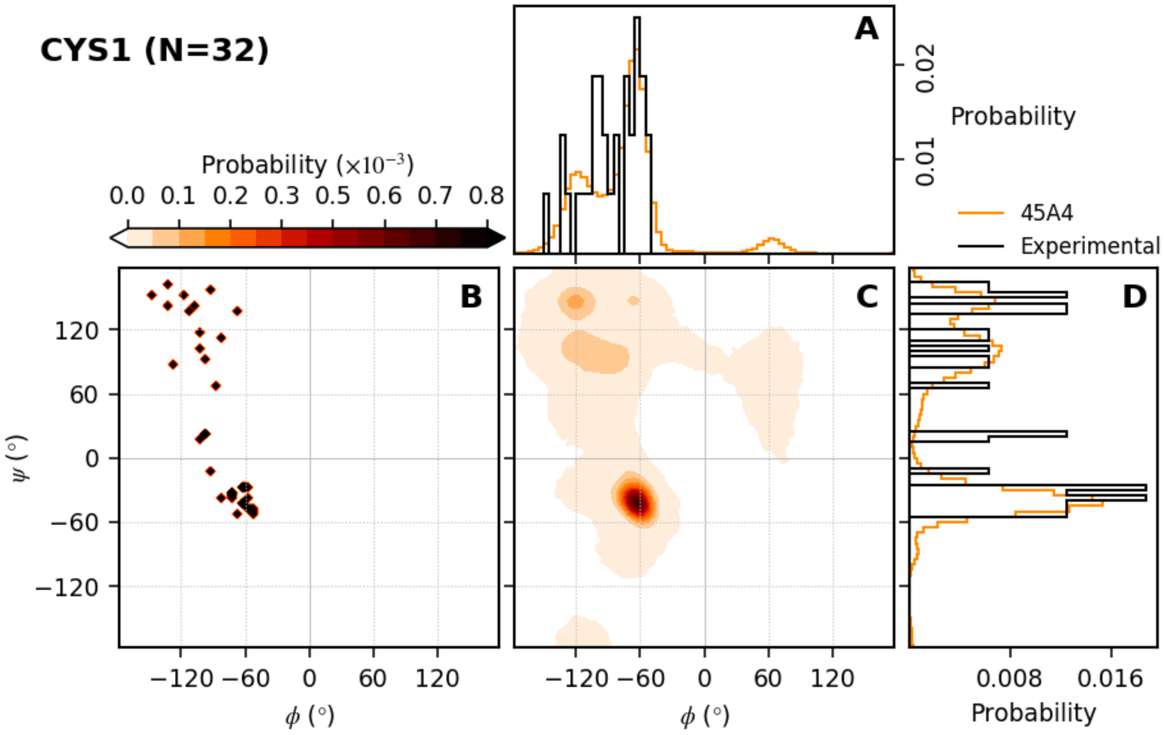
CYS1 (N=32)
A
Probability
0.01
0.02
45A4
Experimental
Probability (×10⁻³)
0.0 0.1 0.2 0.3 0.5 0.6 0.7 0.8
B
C
D
ψ (°)
ϕ (°)
−120 −60 0 60 120
0.008 0.016
Probability

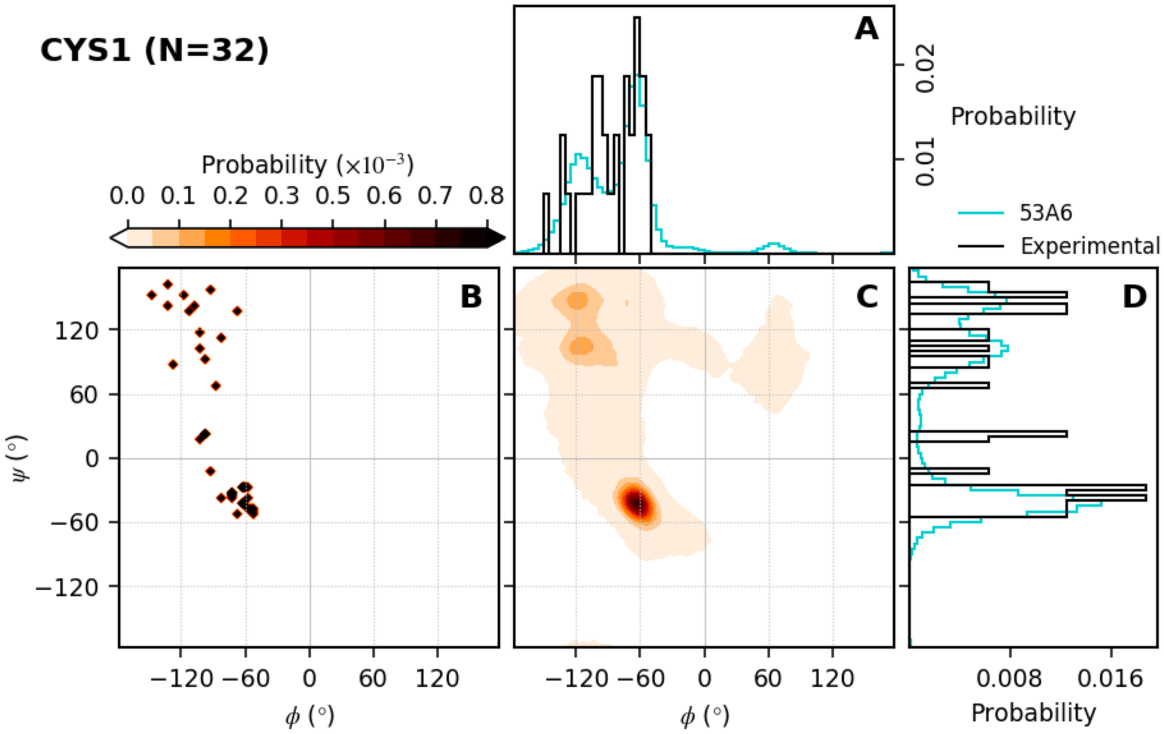
CYS1 (N=32)
A
Probability
0.01
0.02
53A6
Experimental
Probability (×10⁻³)
0.0 0.1 0.2 0.3 0.5 0.6 0.7 0.8
B
C
D
ψ (°)
ϕ (°)
−120 −60 0 60 120
0.008 0.016
Probability

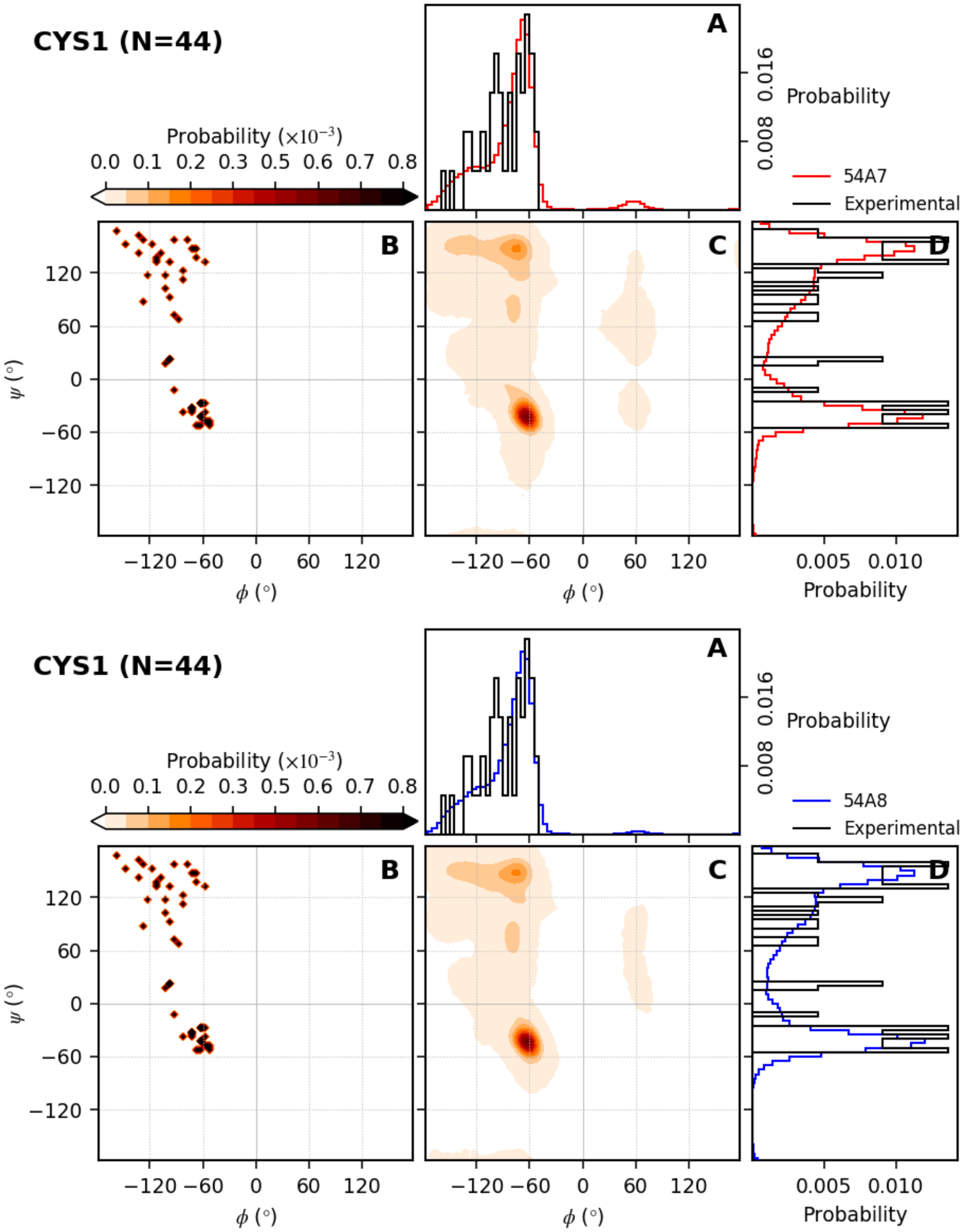

Figure S6. Distribution of first cysteine in S-S bridge (CYS1) backbone dihedral angles.

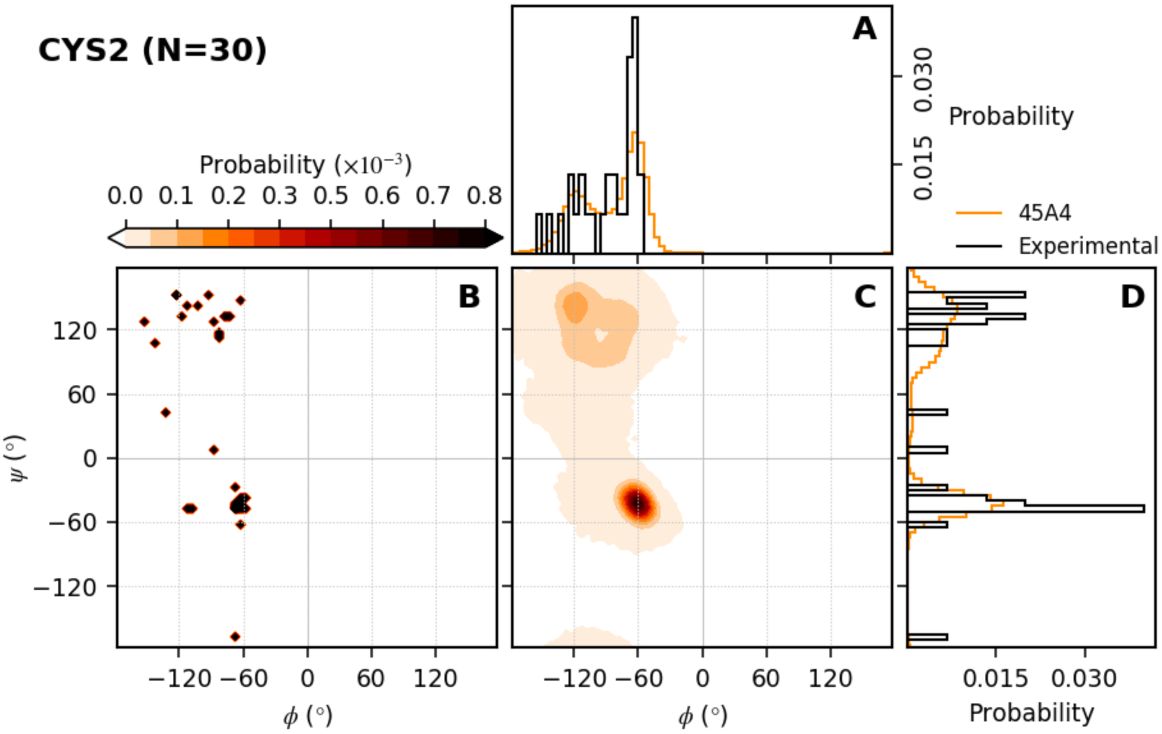
CYS2 (N=30)
Probability (×10⁻³)
0.0 0.1 0.2 0.3 0.5 0.6 0.7 0.8
A
B
C
D
Probability
45A4
Experimental
ψ (°)
φ (°)

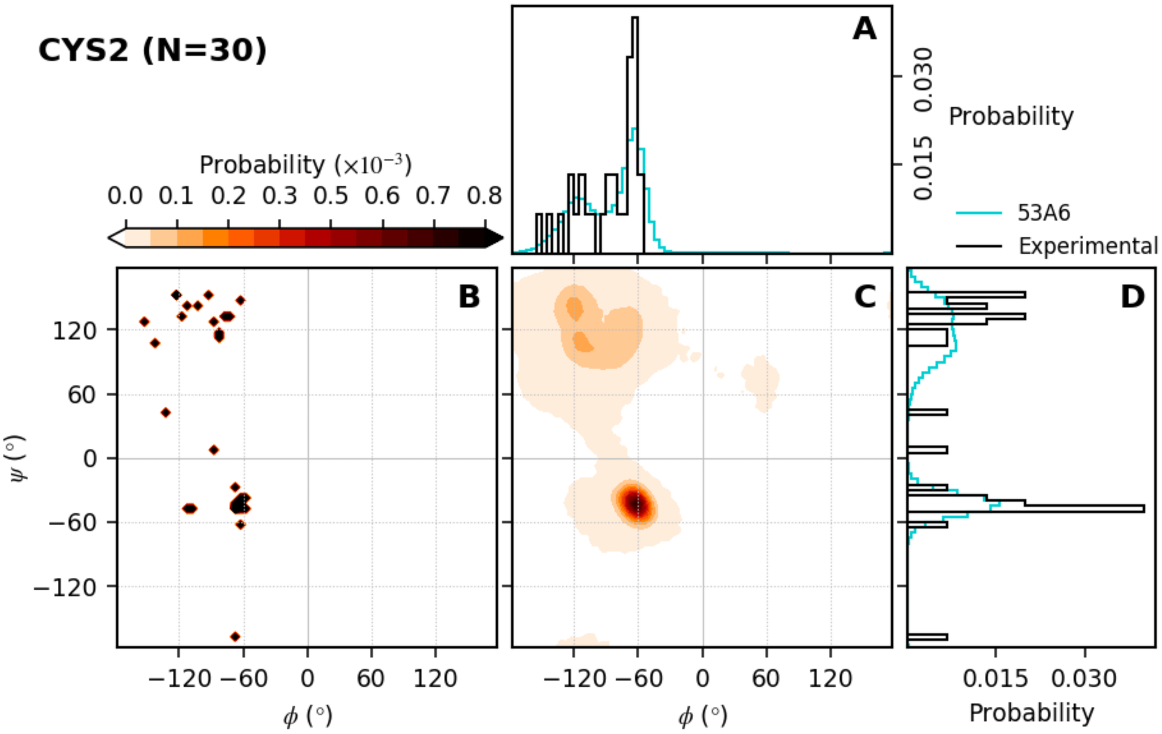
CYS2 (N=30)
Probability (×10⁻³)
0.0 0.1 0.2 0.3 0.5 0.6 0.7 0.8
A
B
C
D
Probability
53A6
Experimental
ψ (°)
φ (°)

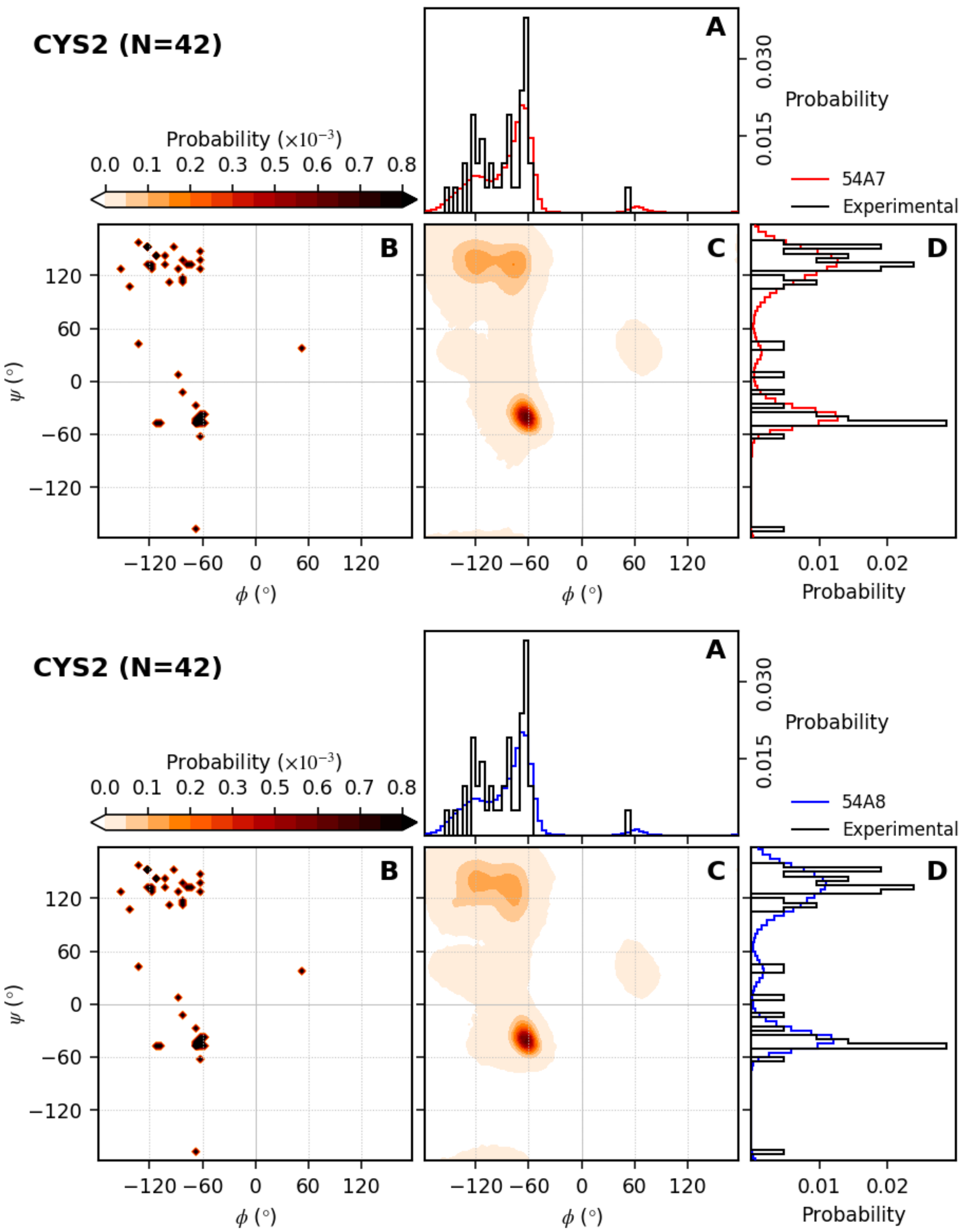

Figure S7. Distribution of second cysteine in S-S bridge (CYS2) backbone dihedral angles.

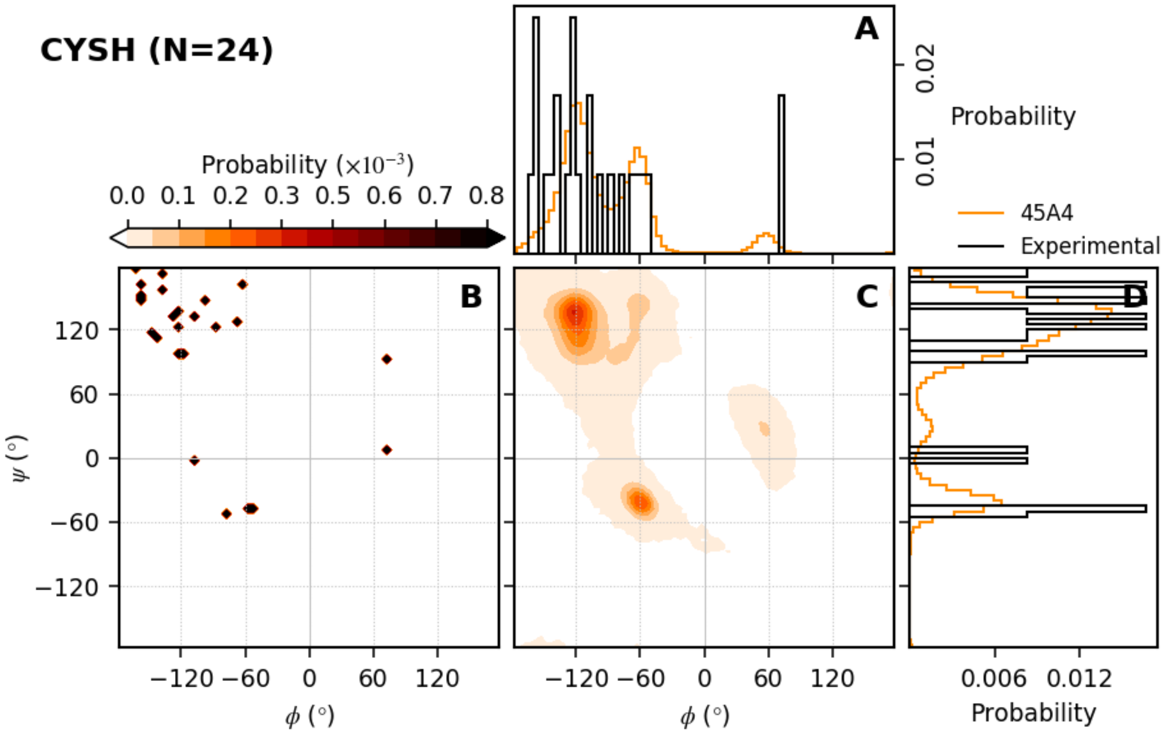
CYSH (N=24)
Probability (×10⁻³)
0.0 0.1 0.2 0.3 0.5 0.6 0.7 0.8
A
B
C
D
Probability
45A4
Experimental
ψ (°)
φ (°)

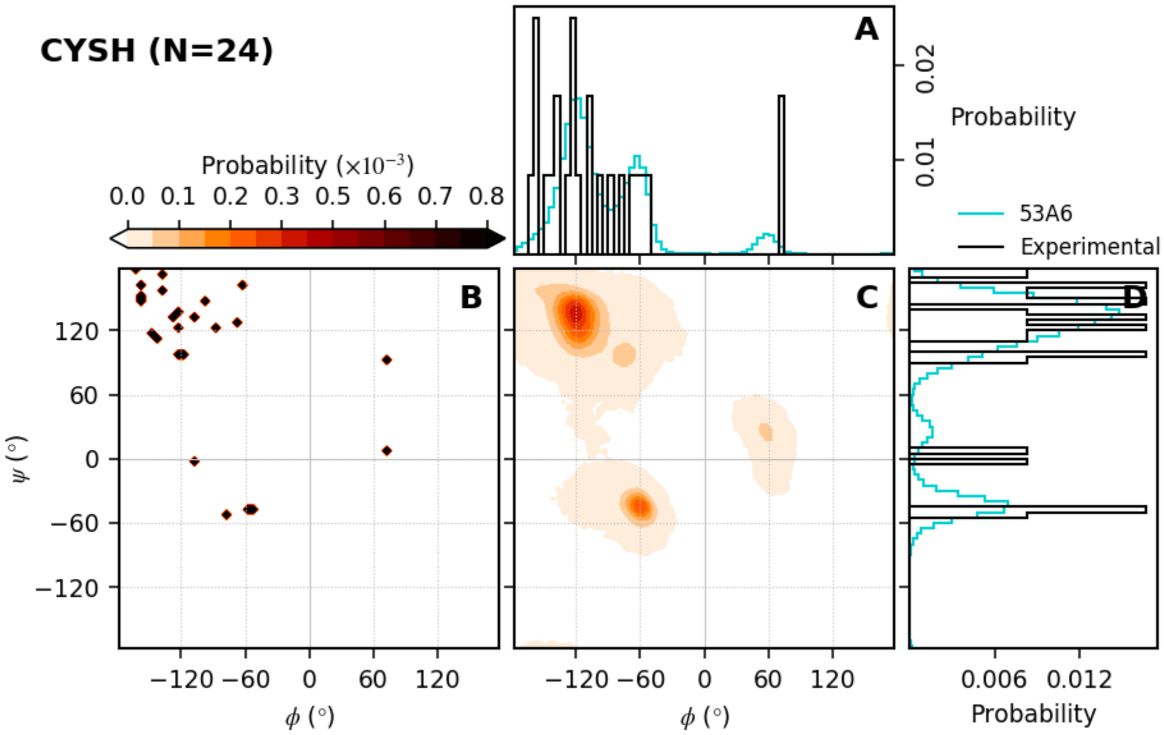
CYSH (N=24)
Probability (×10⁻³)
0.0 0.1 0.2 0.3 0.5 0.6 0.7 0.8
A
B
C
D
Probability
53A6
Experimental
ψ (°)
φ (°)

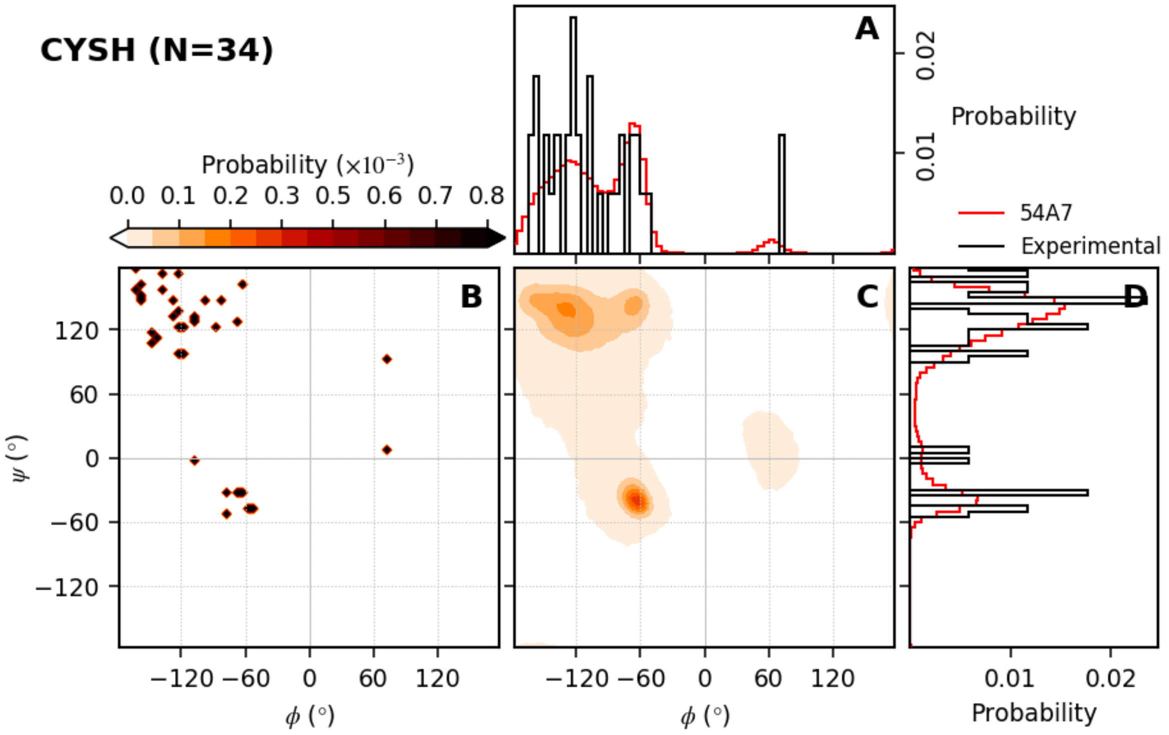

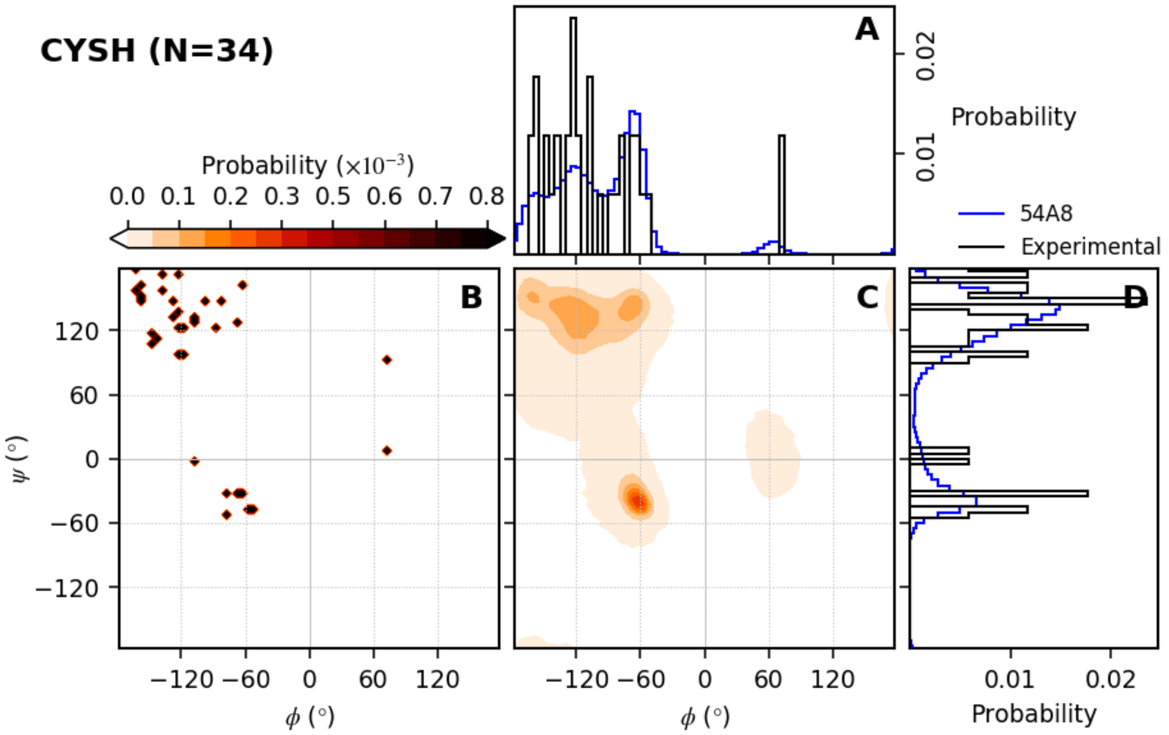

Figure S8. Distribution of neutral cysteine (CYSH) backbone dihedral angles.

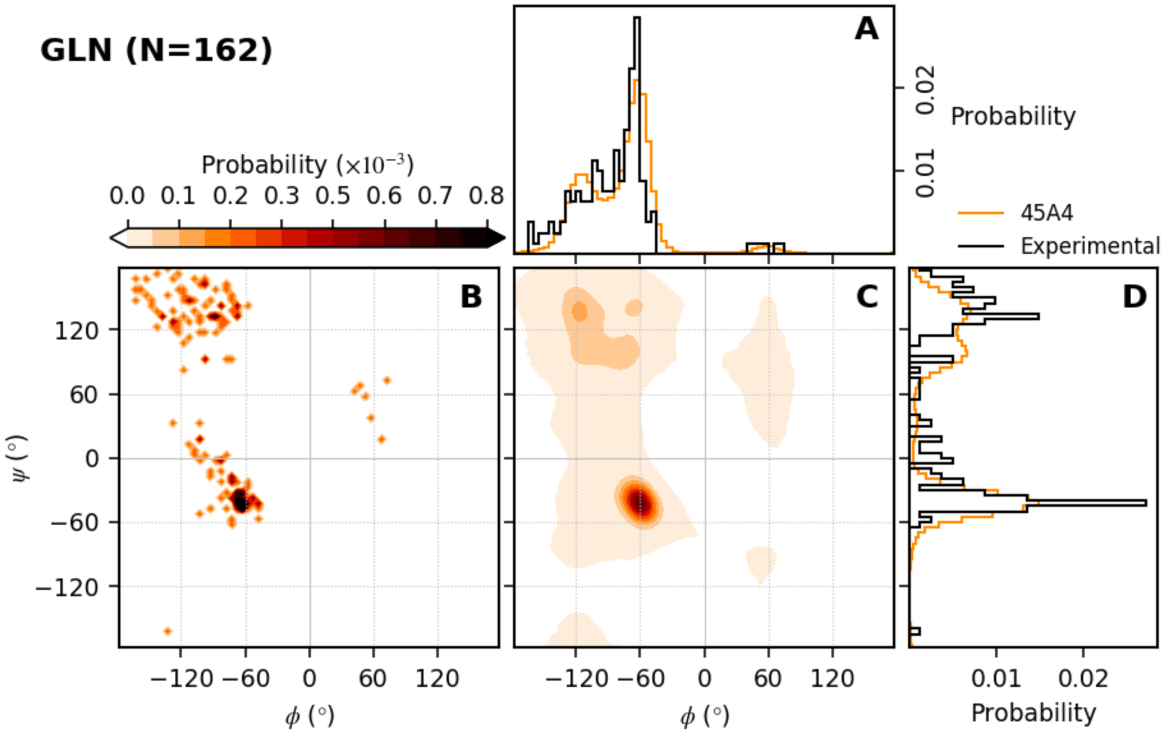
GLN (N=162)
Probability (×10⁻³)
0.0 0.1 0.2 0.3 0.5 0.6 0.7 0.8
A
B
C
D
Probability
45A4
Experimental
ψ (°)
ϕ (°)
Probability

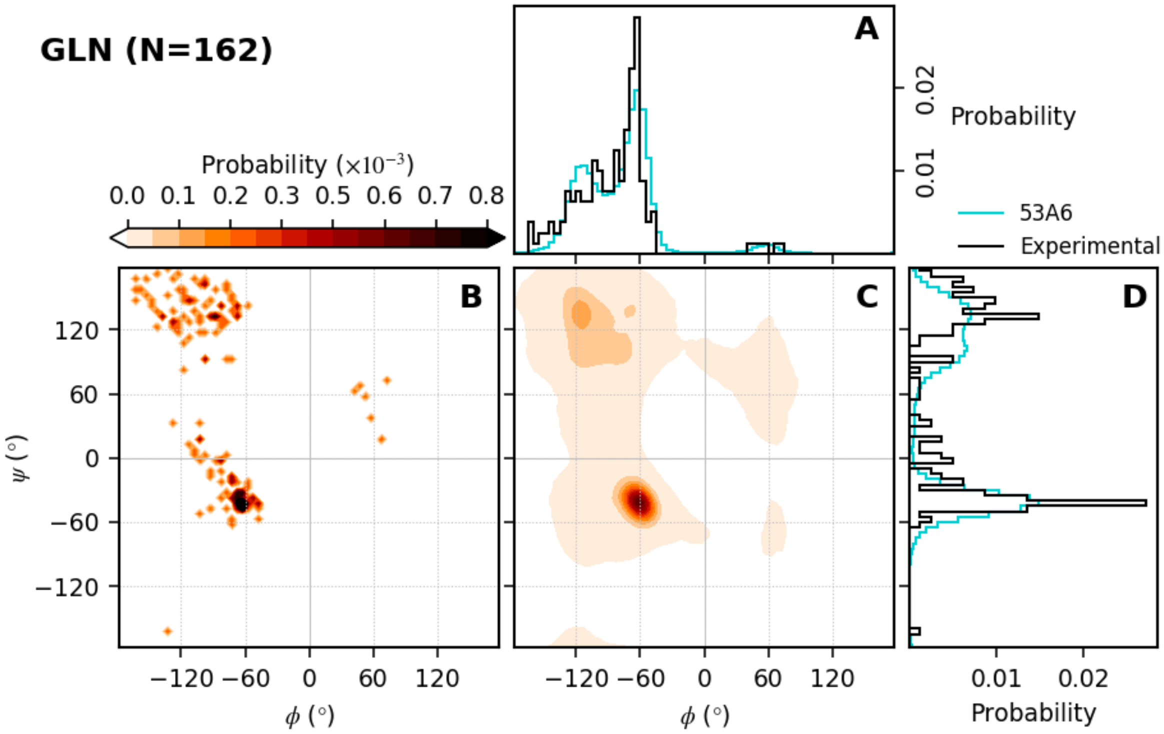
GLN (N=162)
Probability (×10⁻³)
0.0 0.1 0.2 0.3 0.5 0.6 0.7 0.8
A
B
C
D
Probability
53A6
Experimental
ψ (°)
ϕ (°)
Probability

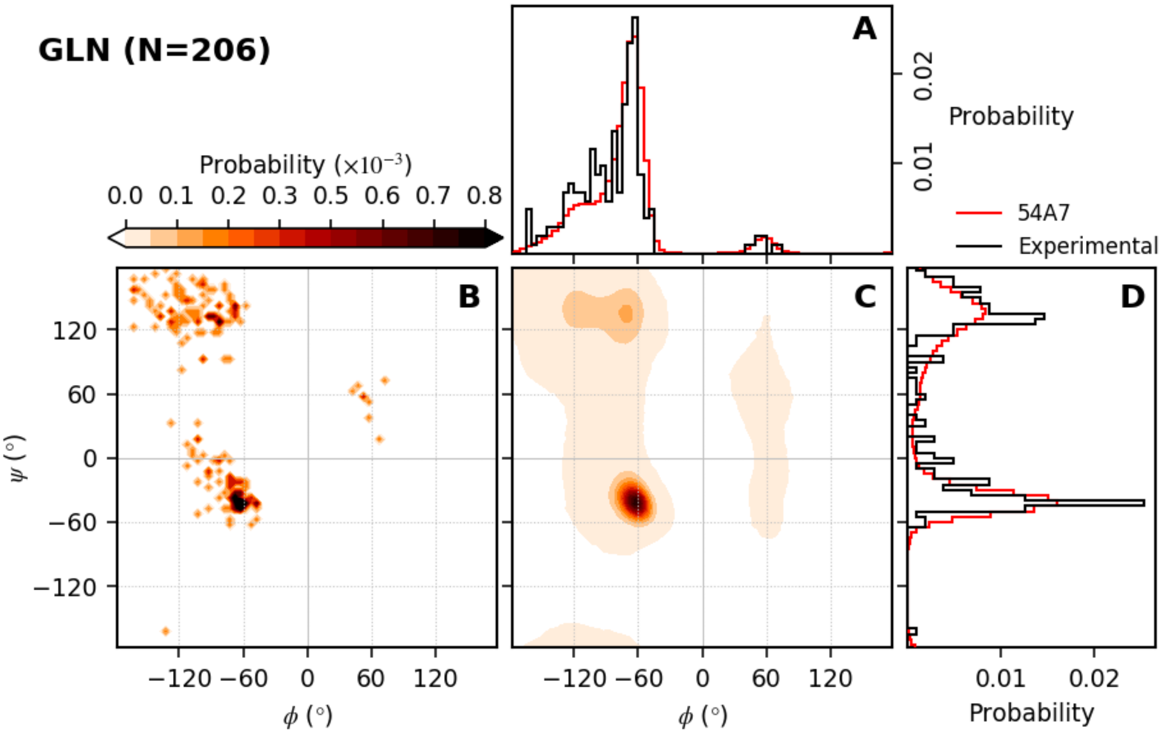

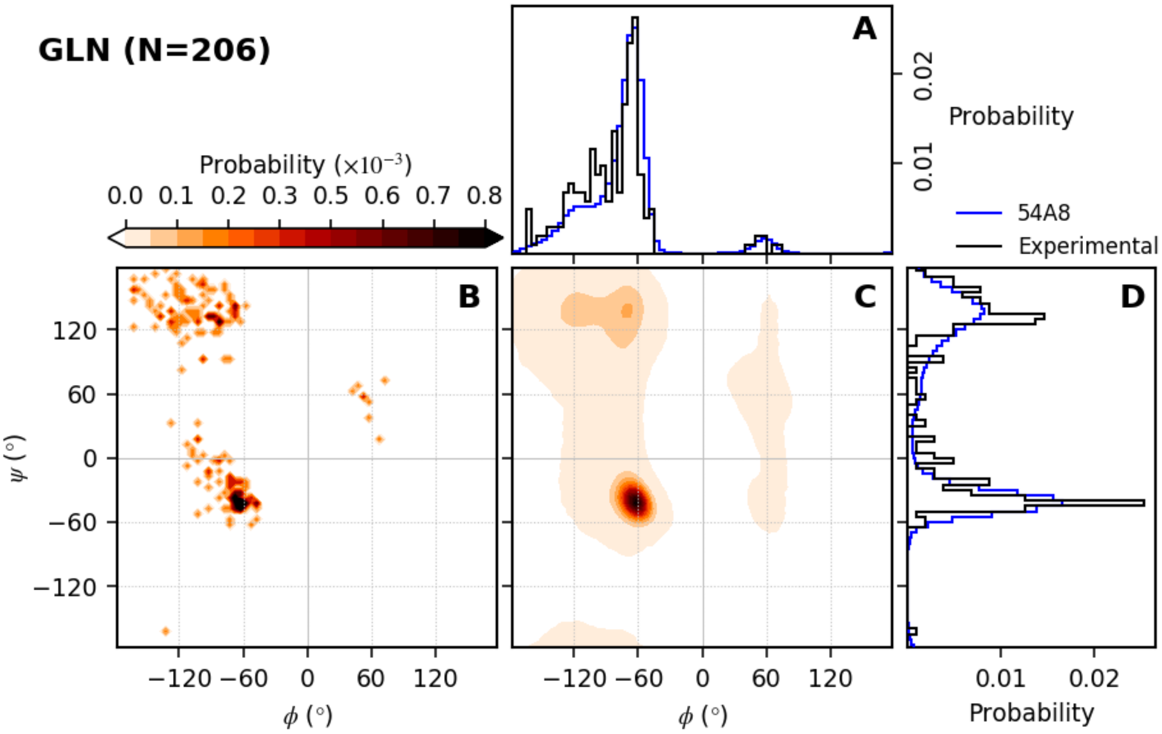

Figure S9. Distribution of glutamine (GLN) backbone dihedral angles.

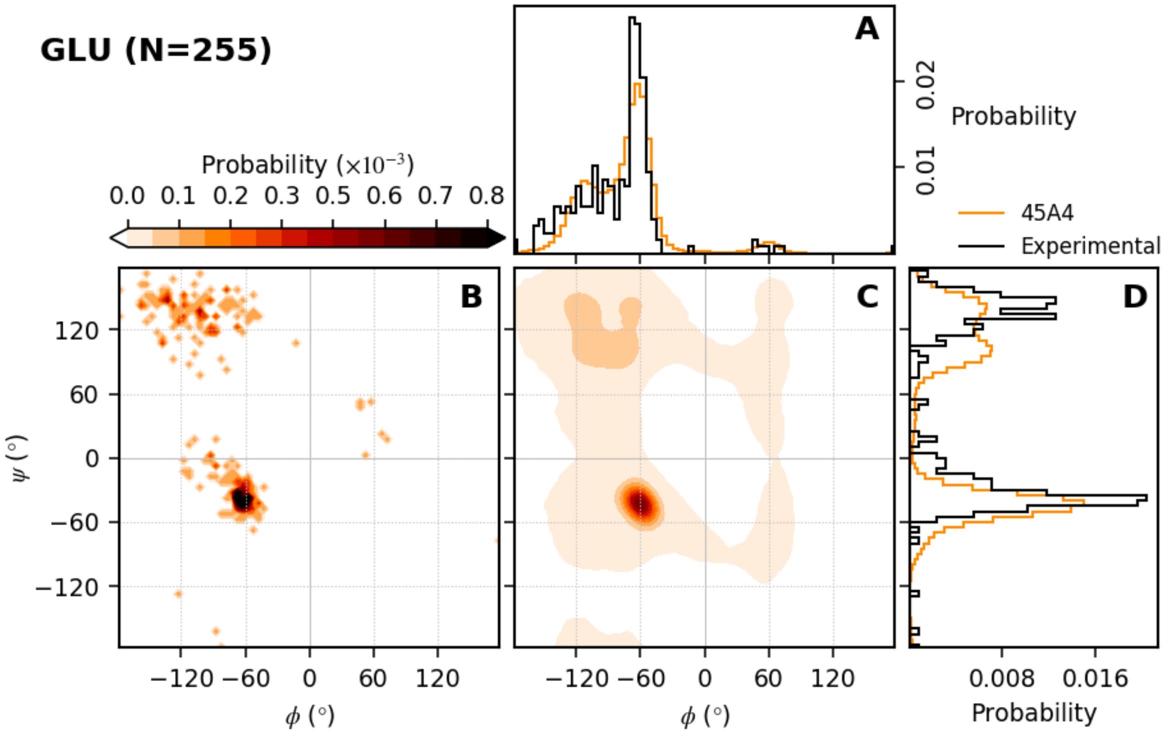
GLU (N=255)
Probability (×10⁻³)
0.0 0.1 0.2 0.3 0.5 0.6 0.7 0.8
A
B
C
D
Probability
45A4
Experimental
ψ (°)
ϕ (°)

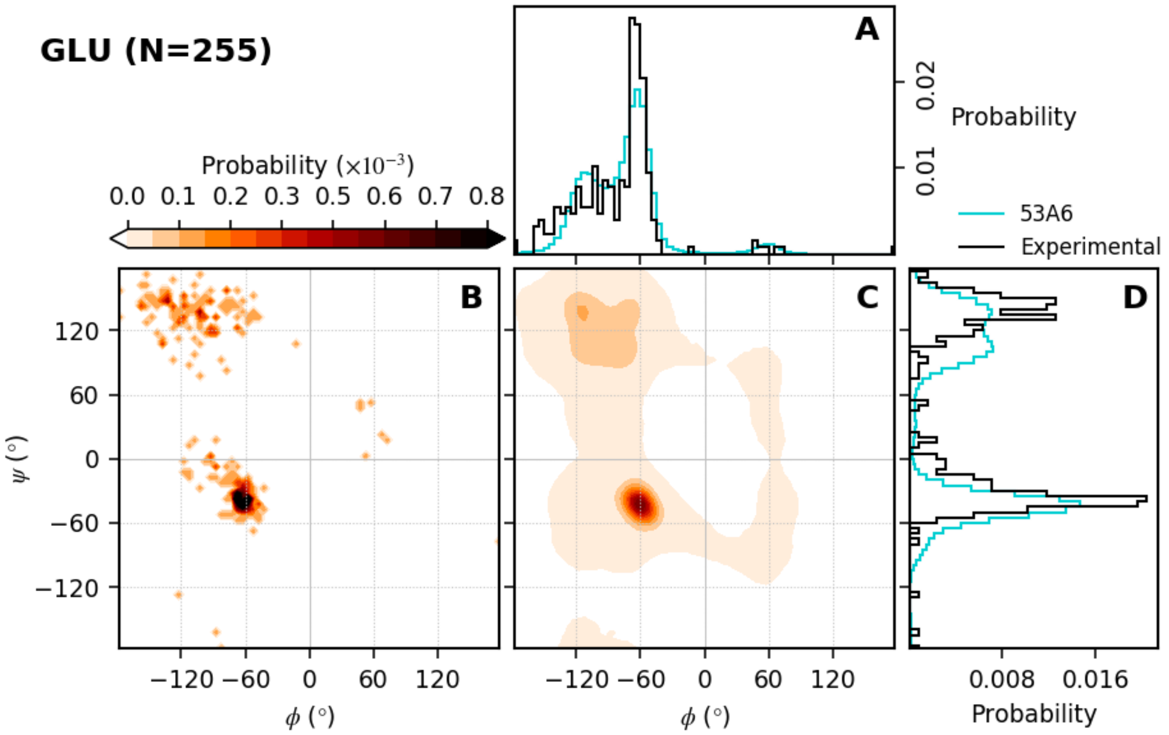
GLU (N=255)
Probability (×10⁻³)
0.0 0.1 0.2 0.3 0.5 0.6 0.7 0.8
A
B
C
D
Probability
53A6
Experimental
ψ (°)
ϕ (°)

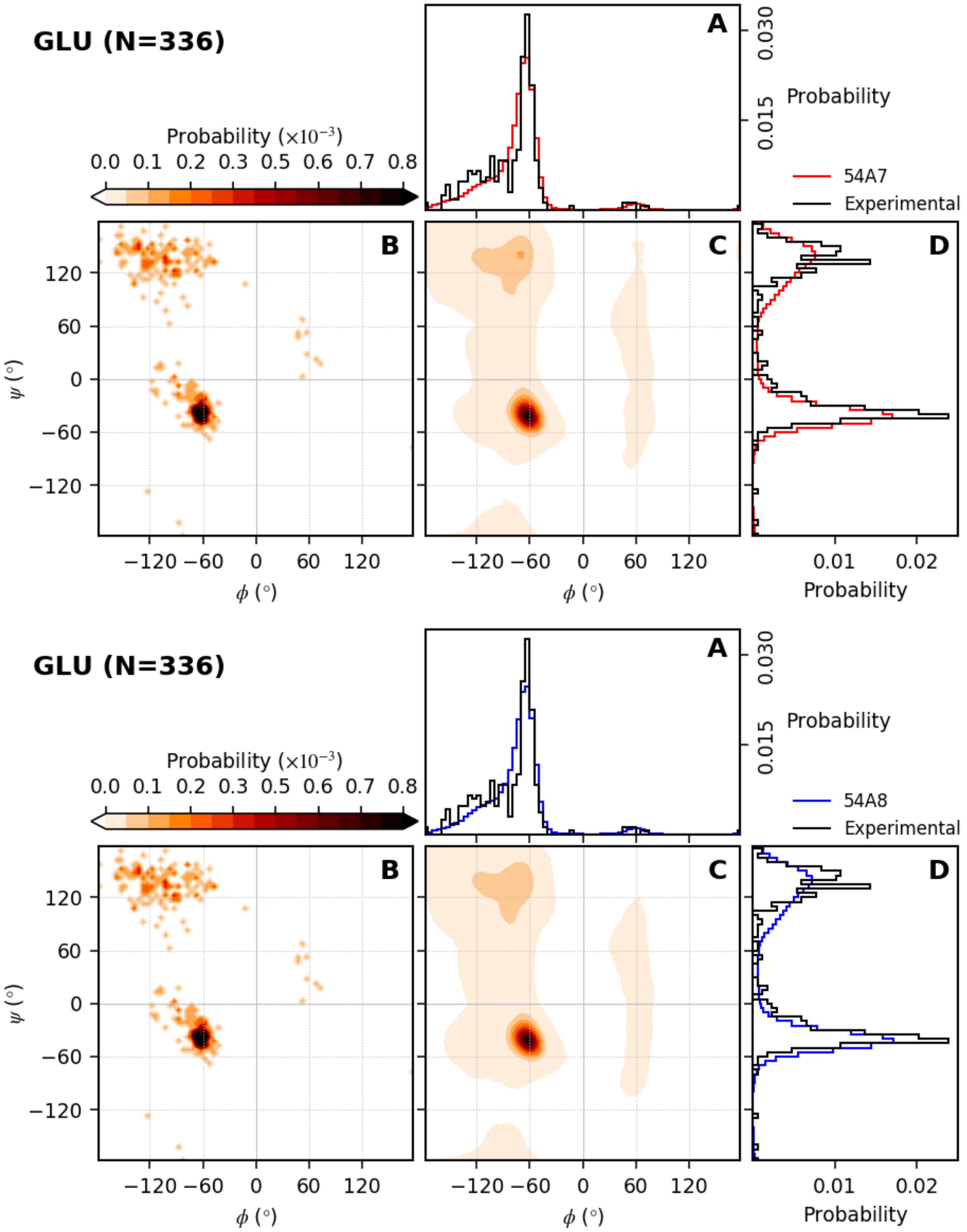

Figure S10. Distribution of glutamic acid (GLU) backbone dihedral angles.

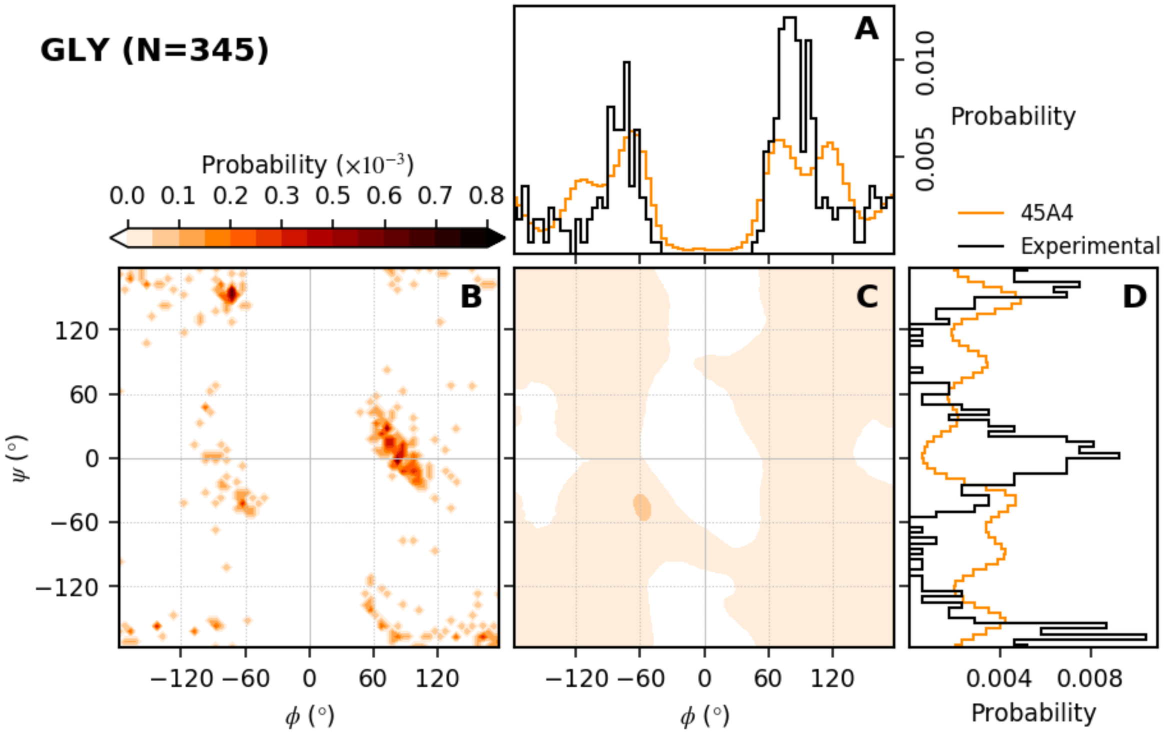
GLY (N=345)
Probability (×10⁻³)
0.0 0.1 0.2 0.3 0.5 0.6 0.7 0.8
A
B
C
D
Probability
0.005
0.010
45A4
Experimental
120
60
0
−60
−120
ψ (°)
−120 −60 0 60 120
ϕ (°)
0.004 0.008

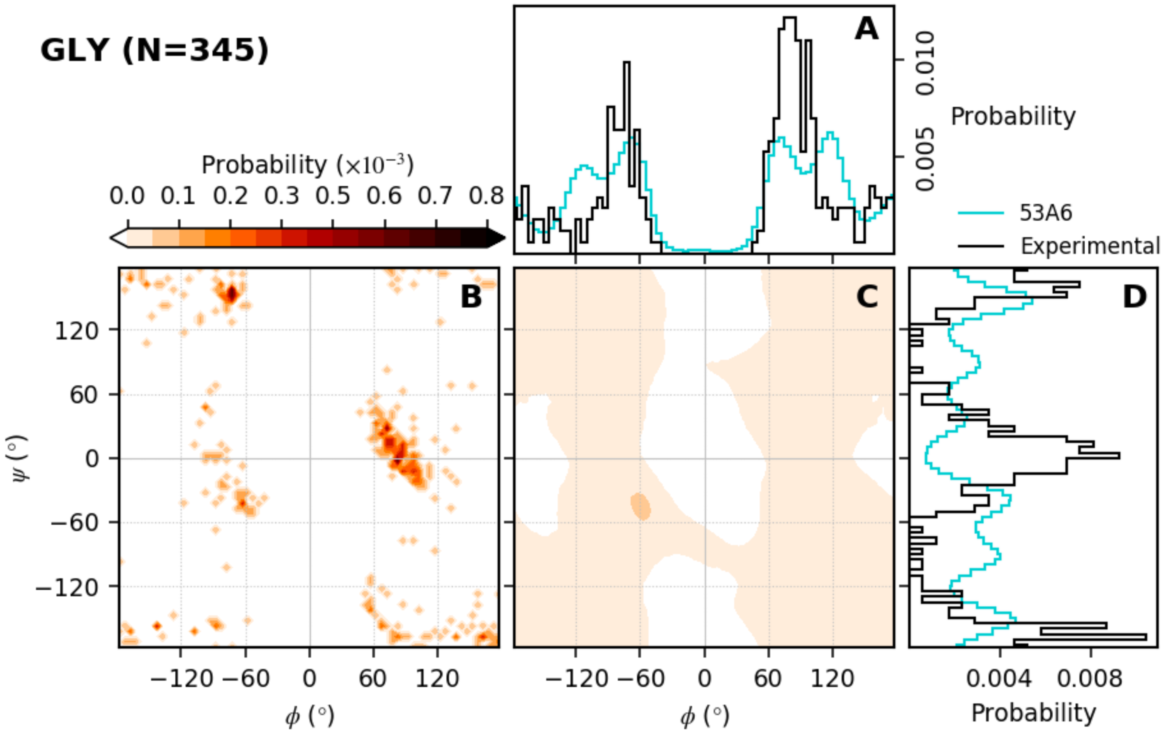
GLY (N=345)
Probability (×10⁻³)
0.0 0.1 0.2 0.3 0.5 0.6 0.7 0.8
A
B
C
D
Probability
0.005
0.010
53A6
Experimental
120
60
0
−60
−120
ψ (°)
−120 −60 0 60 120
ϕ (°)
0.004 0.008

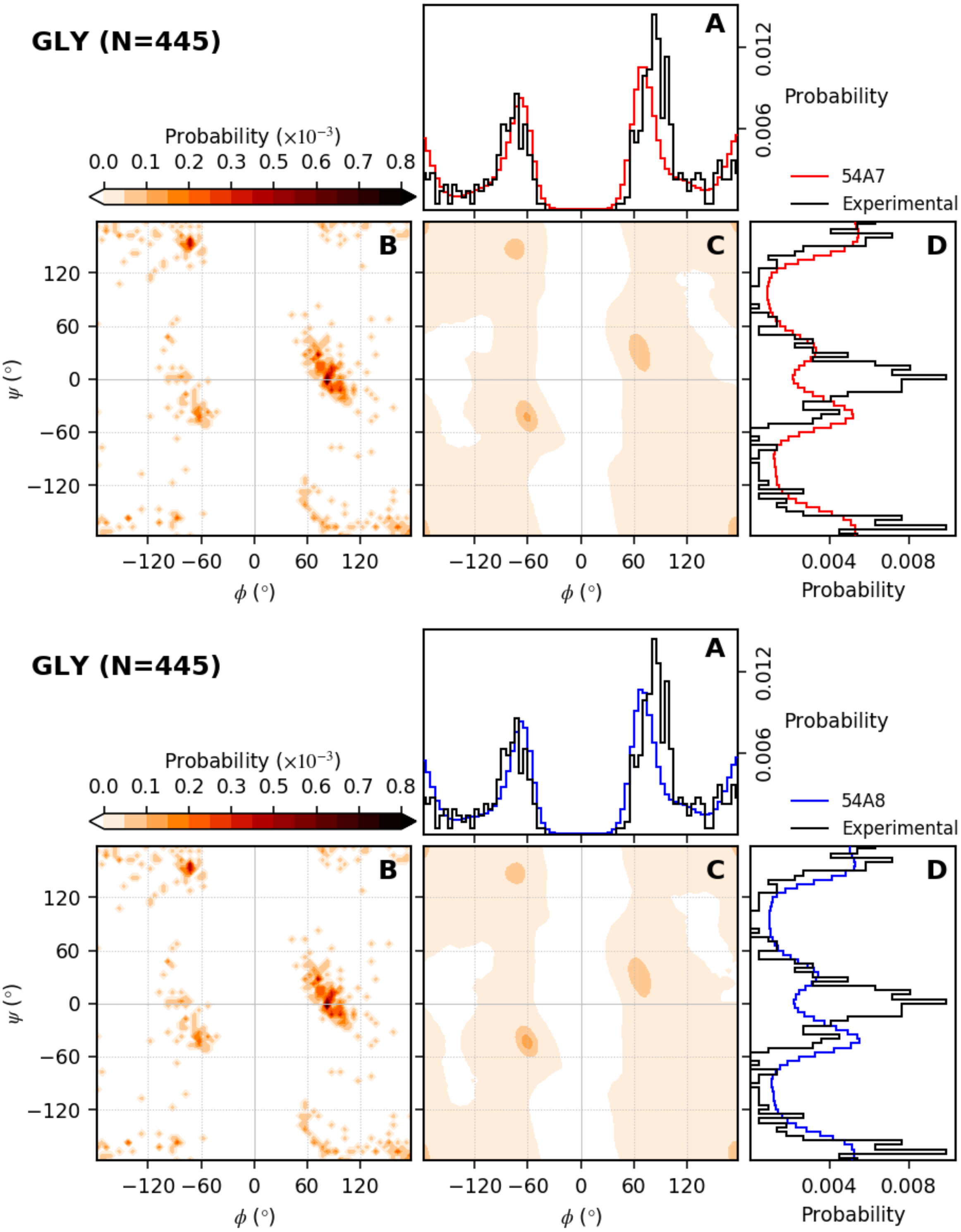

Figure S11. Distribution of glycine (GLY) backbone dihedral angles.

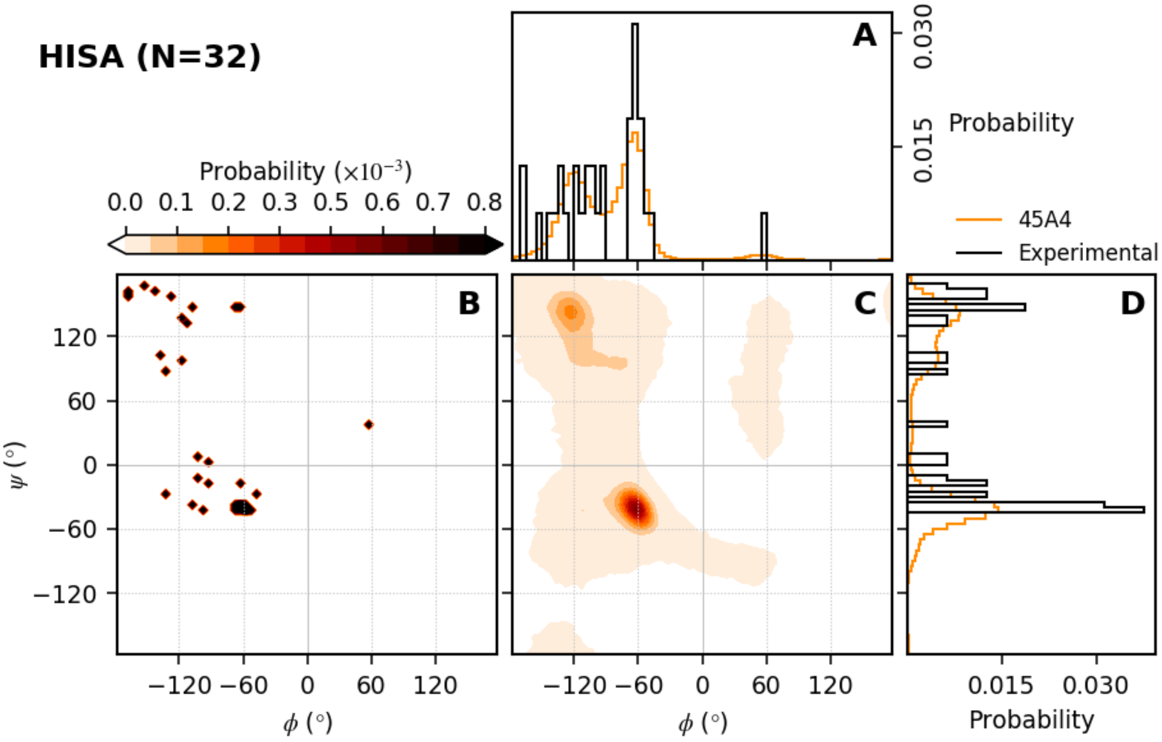
HISA (N=32)
A
B
C
D
Probability (×10⁻³)
0.0 0.1 0.2 0.3 0.5 0.6 0.7 0.8
Probability
0.015
0.030
45A4
Experimental
ψ (°)
φ (°)
120
60
0
−60
−120

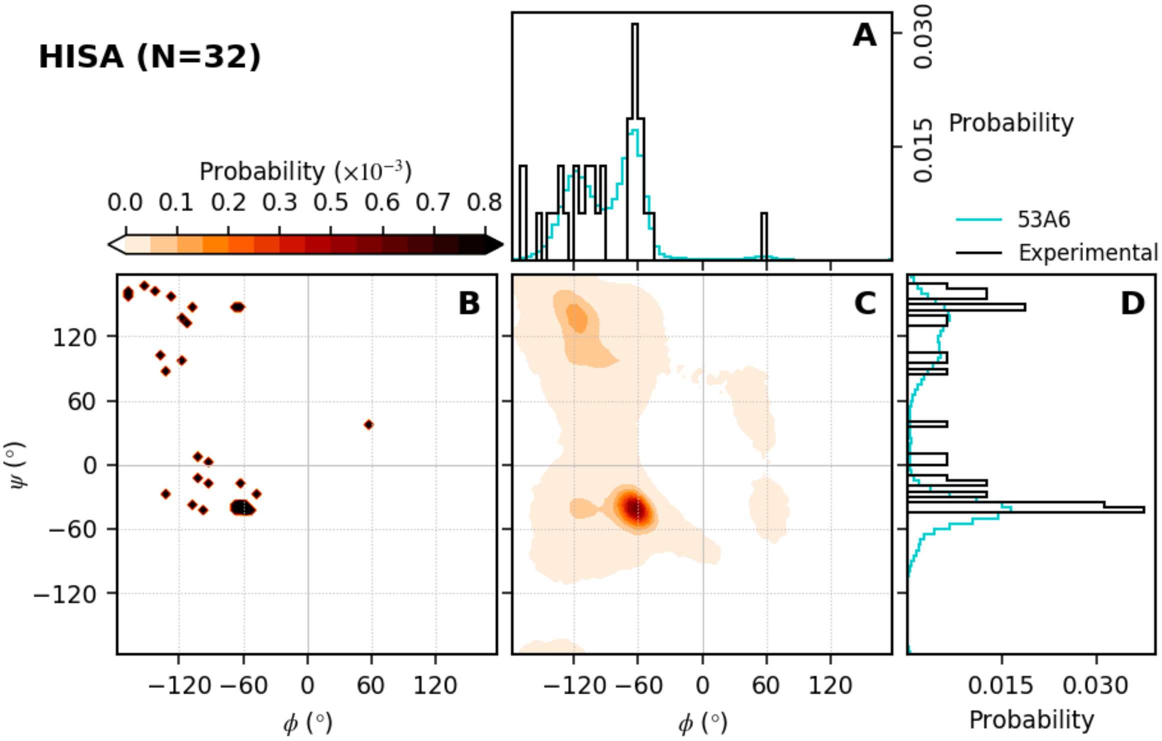
HISA (N=32)
A
B
C
D
Probability (×10⁻³)
0.0 0.1 0.2 0.3 0.5 0.6 0.7 0.8
Probability
0.015
0.030
53A6
Experimental
ψ (°)
φ (°)
120
60
0
−60
−120

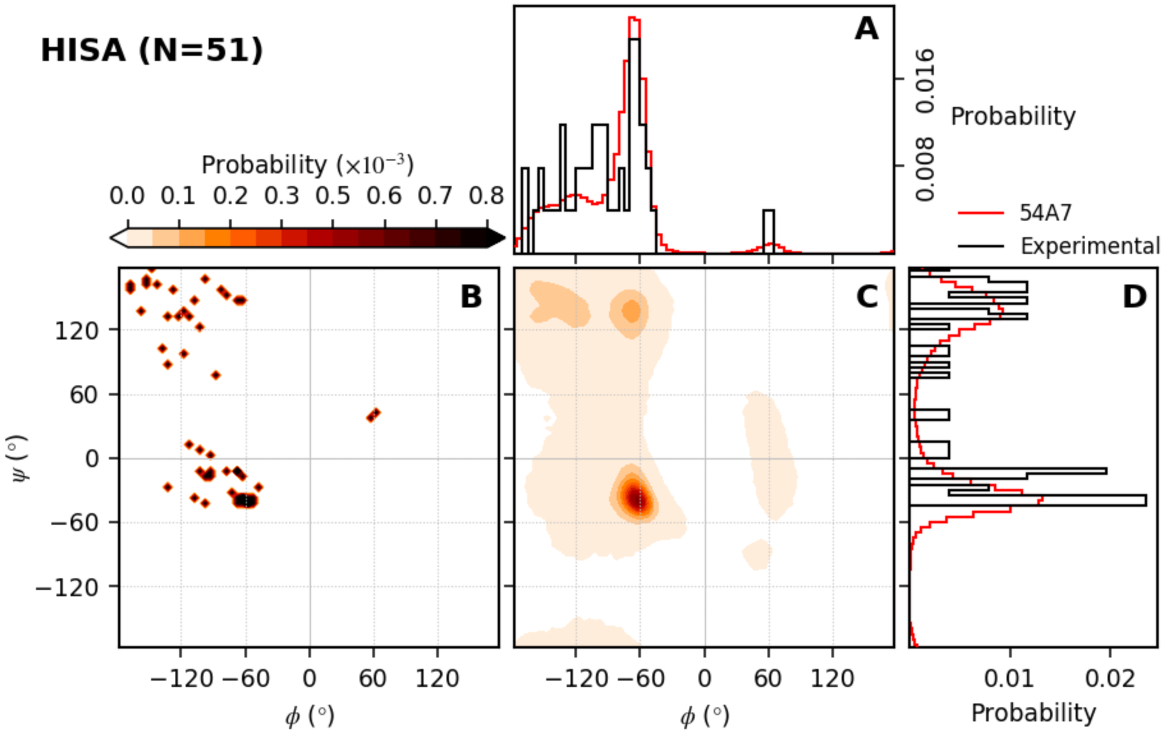

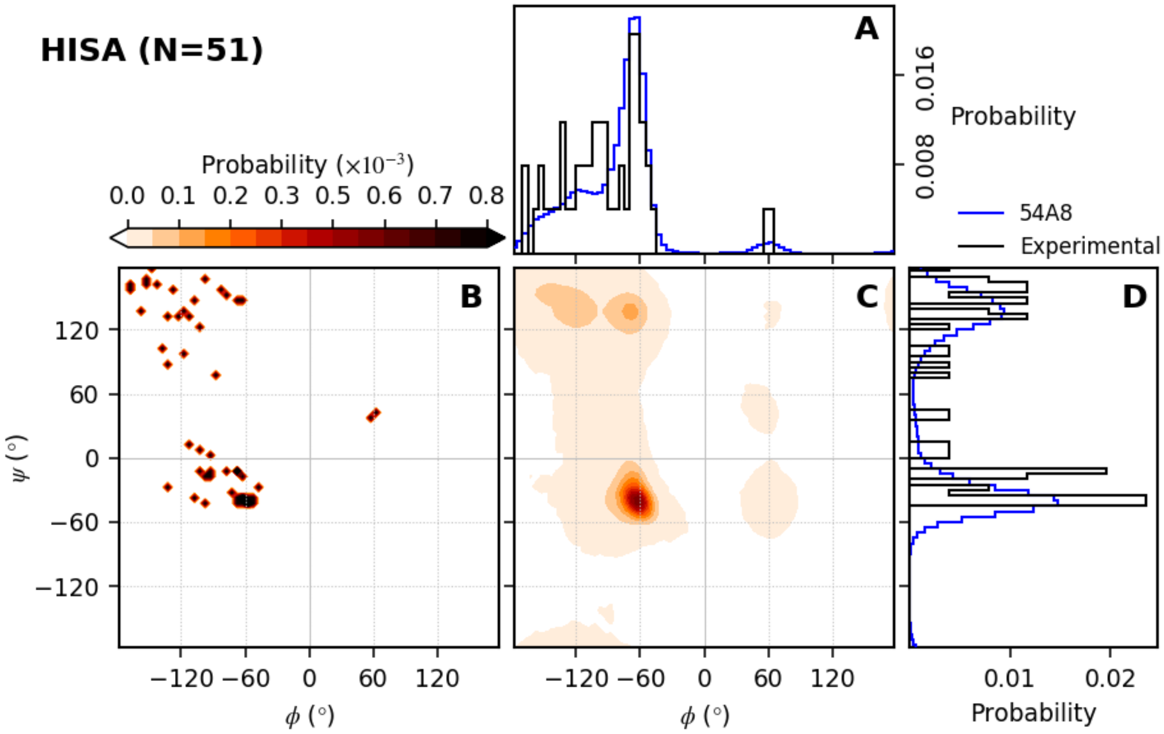

Figure S12. Distribution of histidine protonated at ND1 (HISA) backbone dihedral angles.

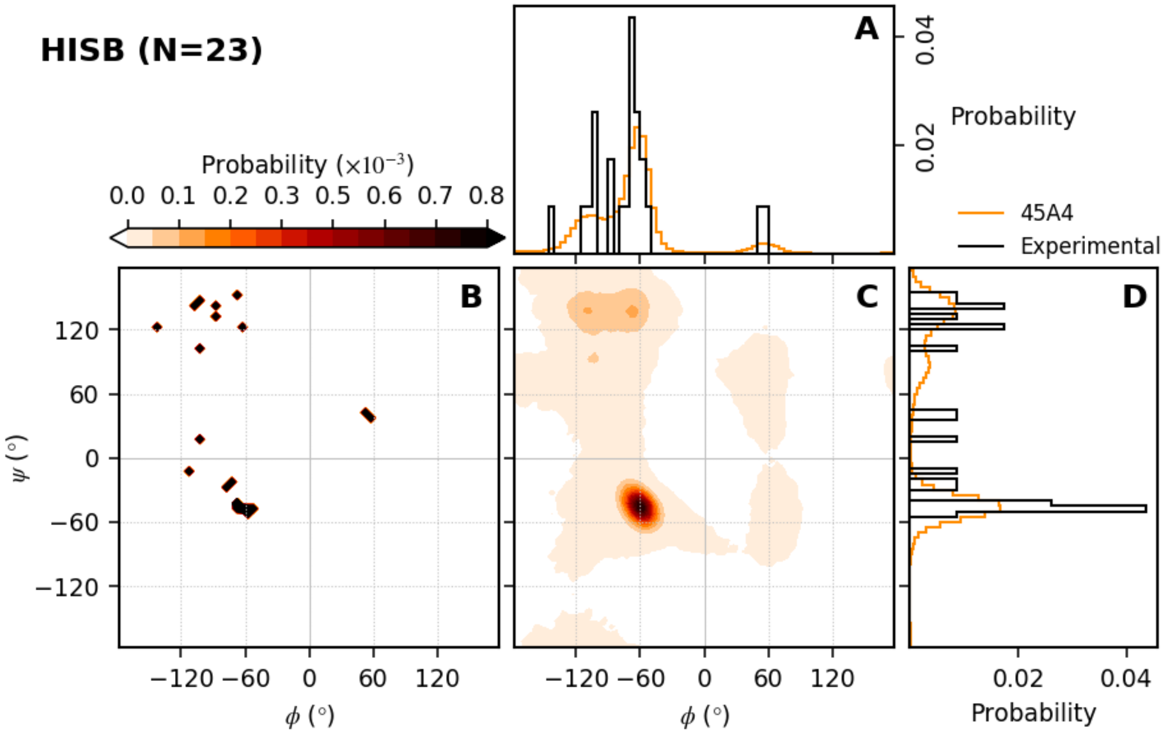
HISB (N=23)
Probability (×10⁻³)
0.0 0.1 0.2 0.3 0.5 0.6 0.7 0.8
A
B
C
D
Probability
45A4
Experimental
ψ (°)
φ (°)

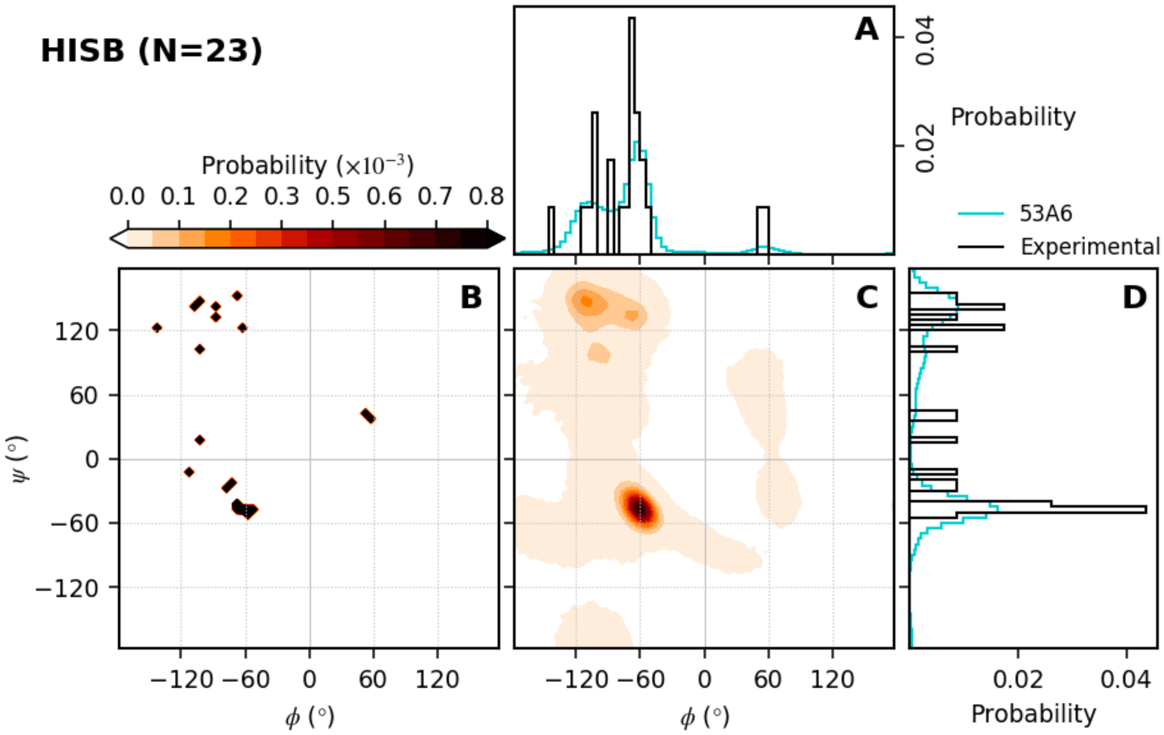
HISB (N=23)
Probability (×10⁻³)
0.0 0.1 0.2 0.3 0.5 0.6 0.7 0.8
A
B
C
D
Probability
53A6
Experimental
ψ (°)
φ (°)

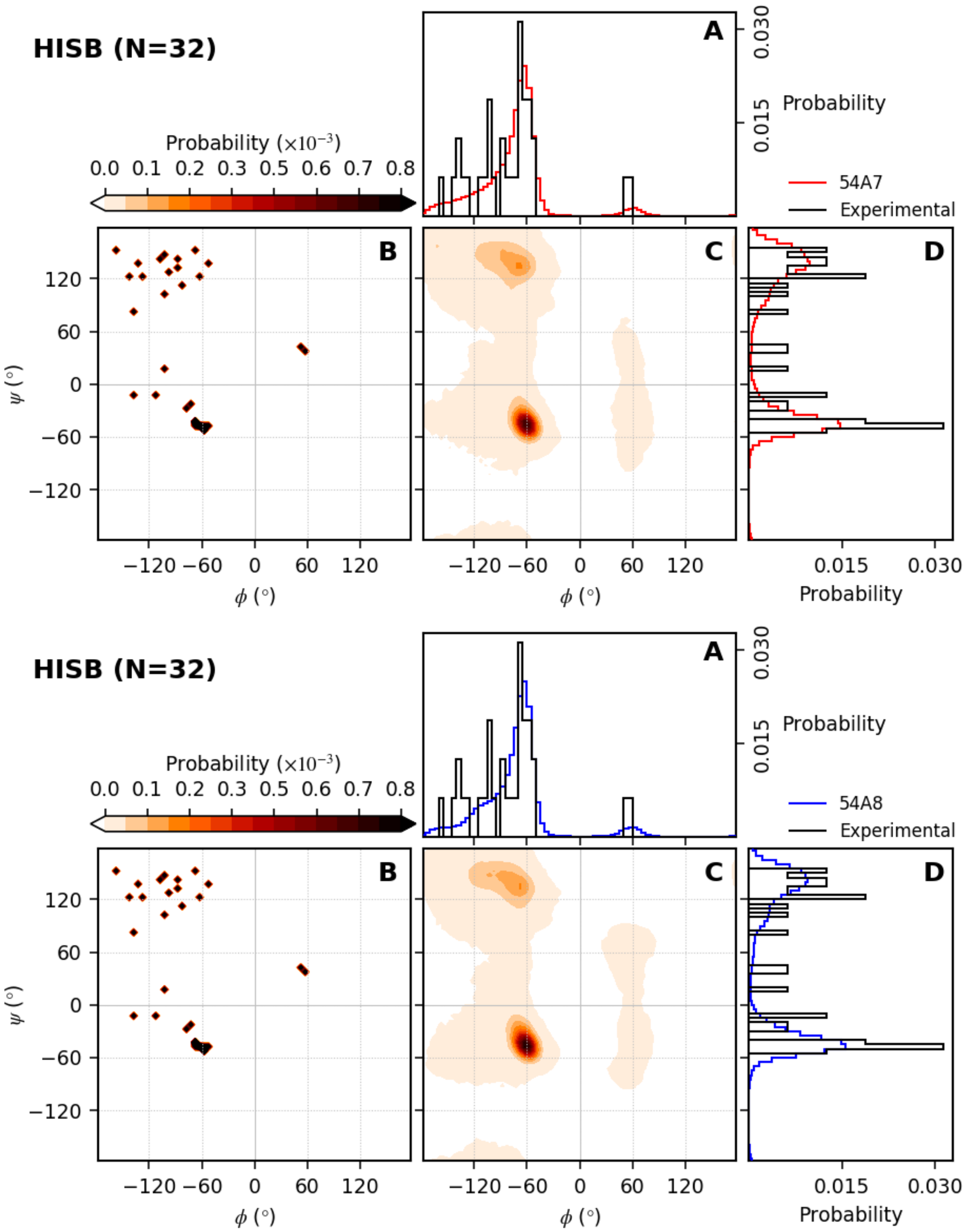

Figure S13. Distribution of histidine protonated at NE2 (HISB) backbone dihedral angles.

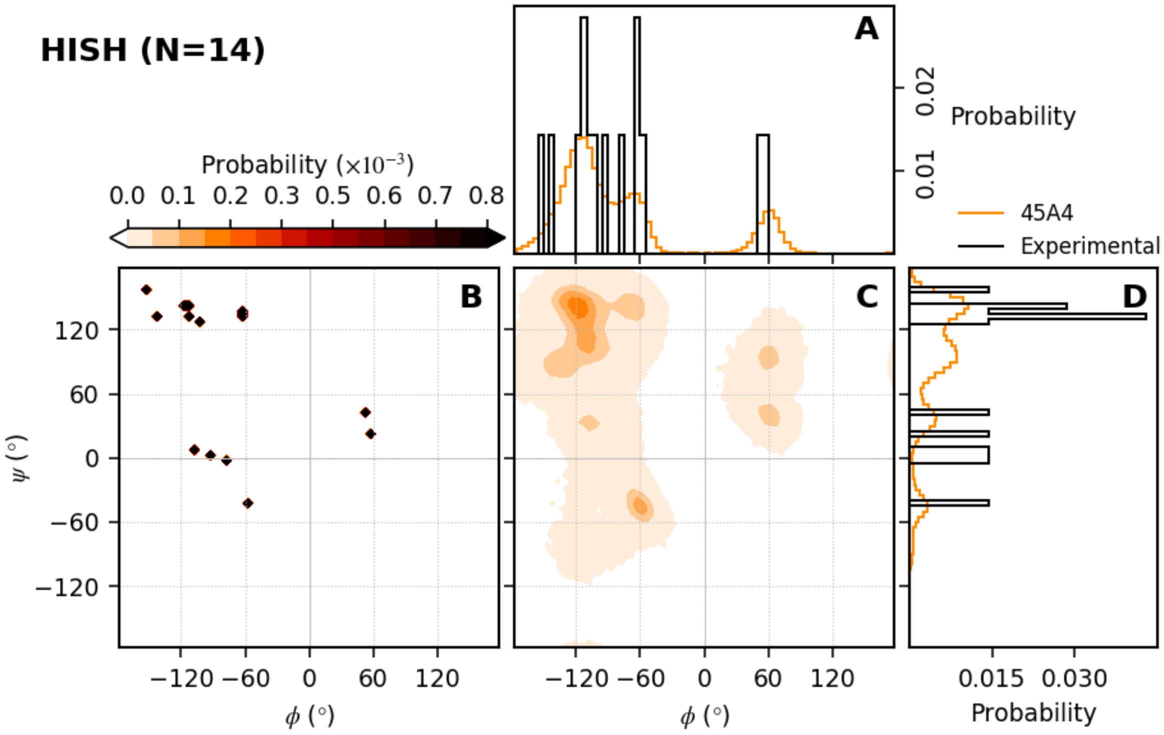
HISH (N=14)
A
B
C
D
Probability (×10⁻³)
0.0 0.1 0.2 0.3 0.5 0.6 0.7 0.8
Probability
45A4
Experimental
ψ (°)
ϕ (°)
Probability

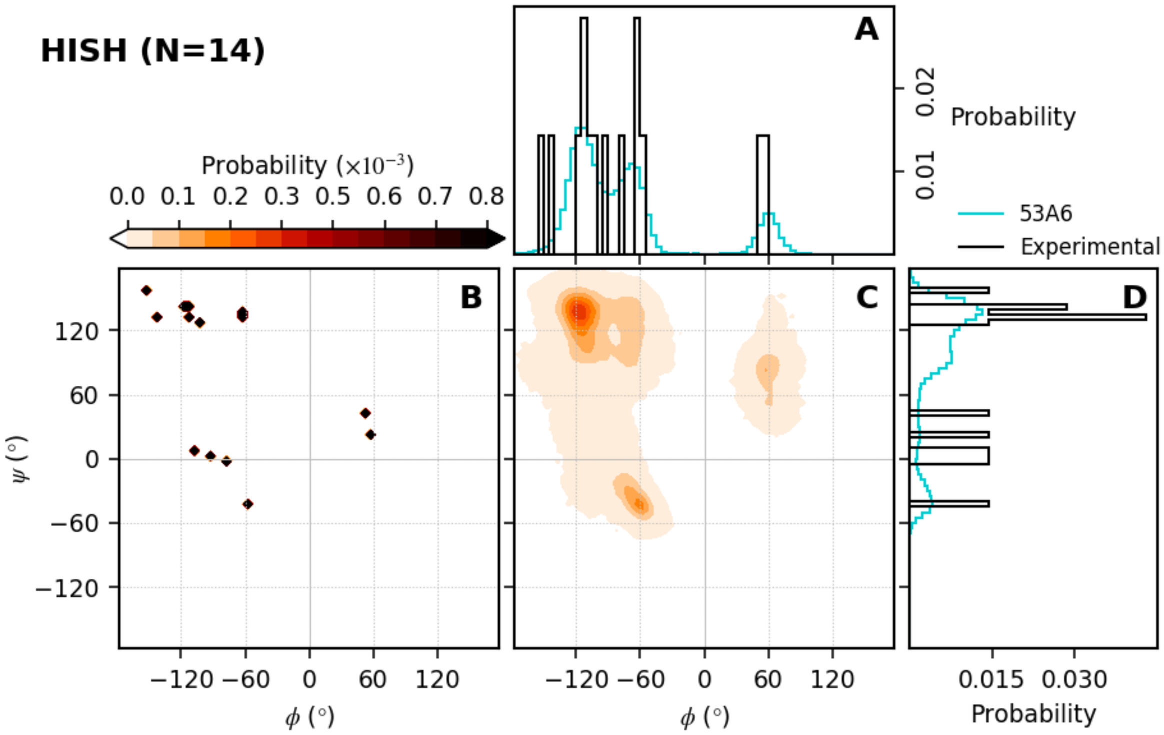
HISH (N=14)
A
B
C
D
Probability (×10⁻³)
0.0 0.1 0.2 0.3 0.5 0.6 0.7 0.8
Probability
53A6
Experimental
ψ (°)
ϕ (°)
Probability

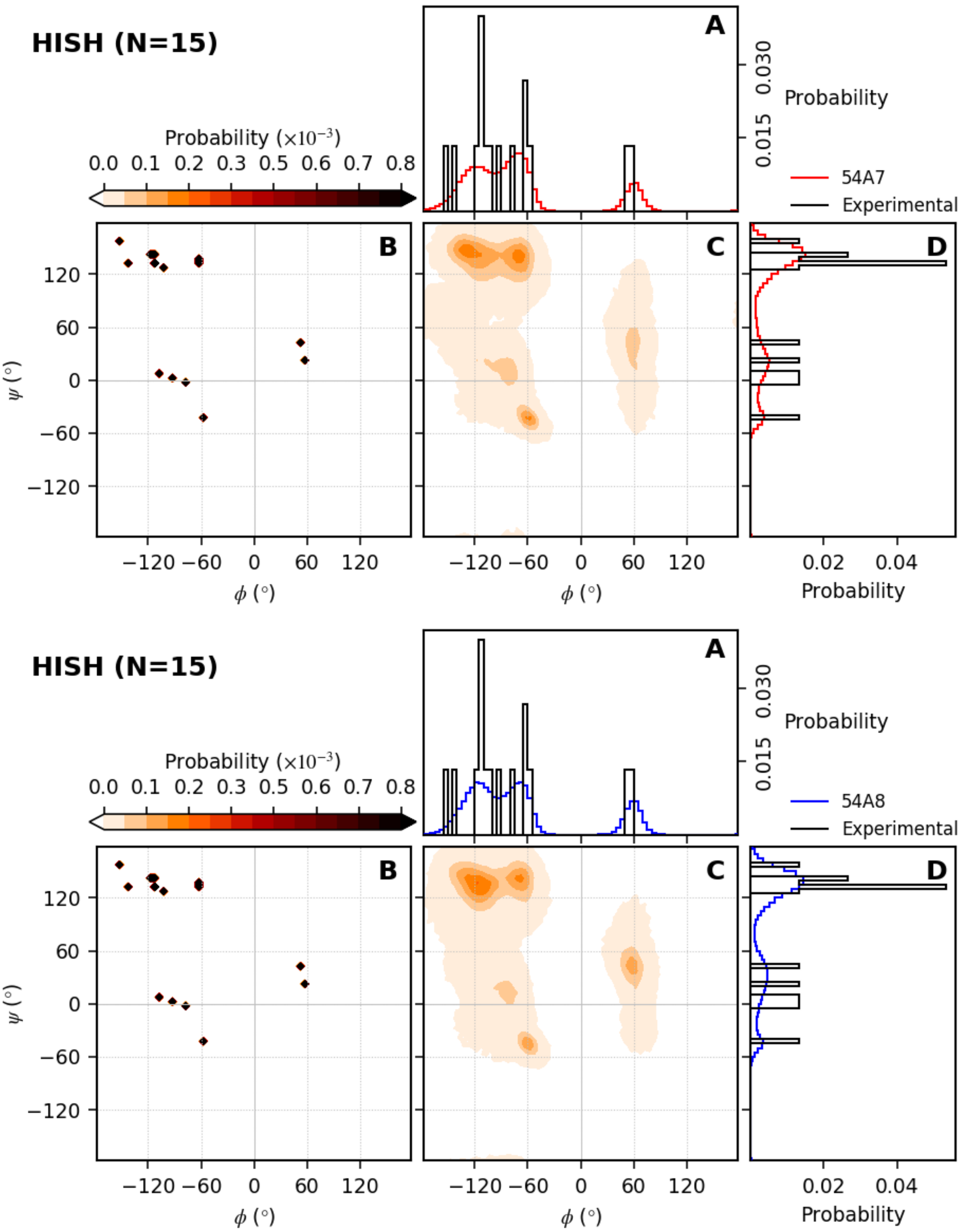

Figure S14. Distribution of histidine with +1 formal charge (HISH) backbone dihedral angles.

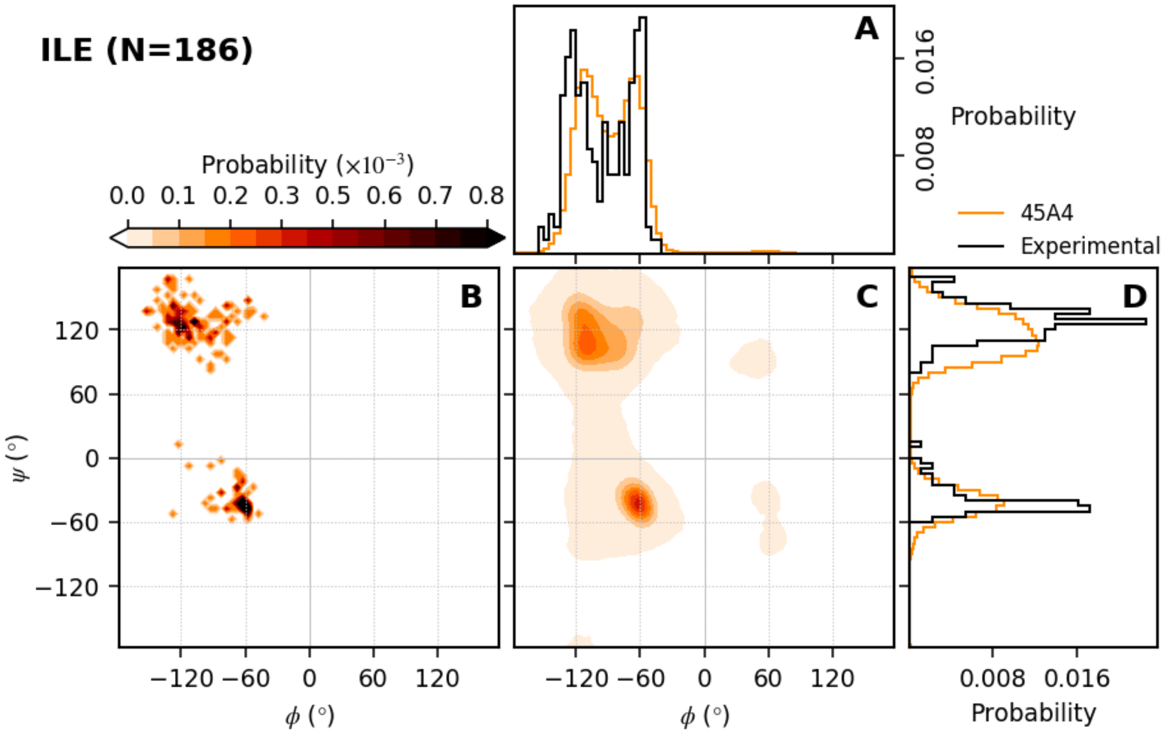
ILE (N=186)
Probability (×10⁻³)
0.0 0.1 0.2 0.3 0.5 0.6 0.7 0.8
A
B
C
D
Probability
45A4
Experimental
ψ (°)
ϕ (°)

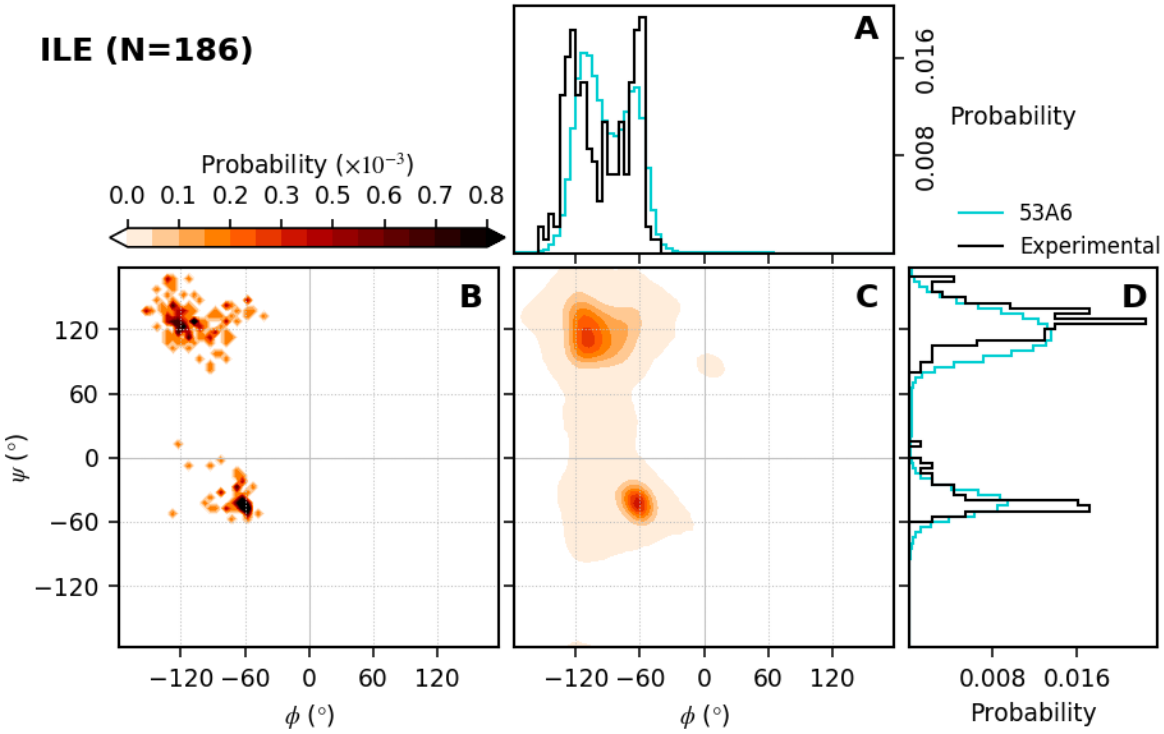
ILE (N=186)
Probability (×10⁻³)
0.0 0.1 0.2 0.3 0.5 0.6 0.7 0.8
A
B
C
D
Probability
53A6
Experimental
ψ (°)
ϕ (°)

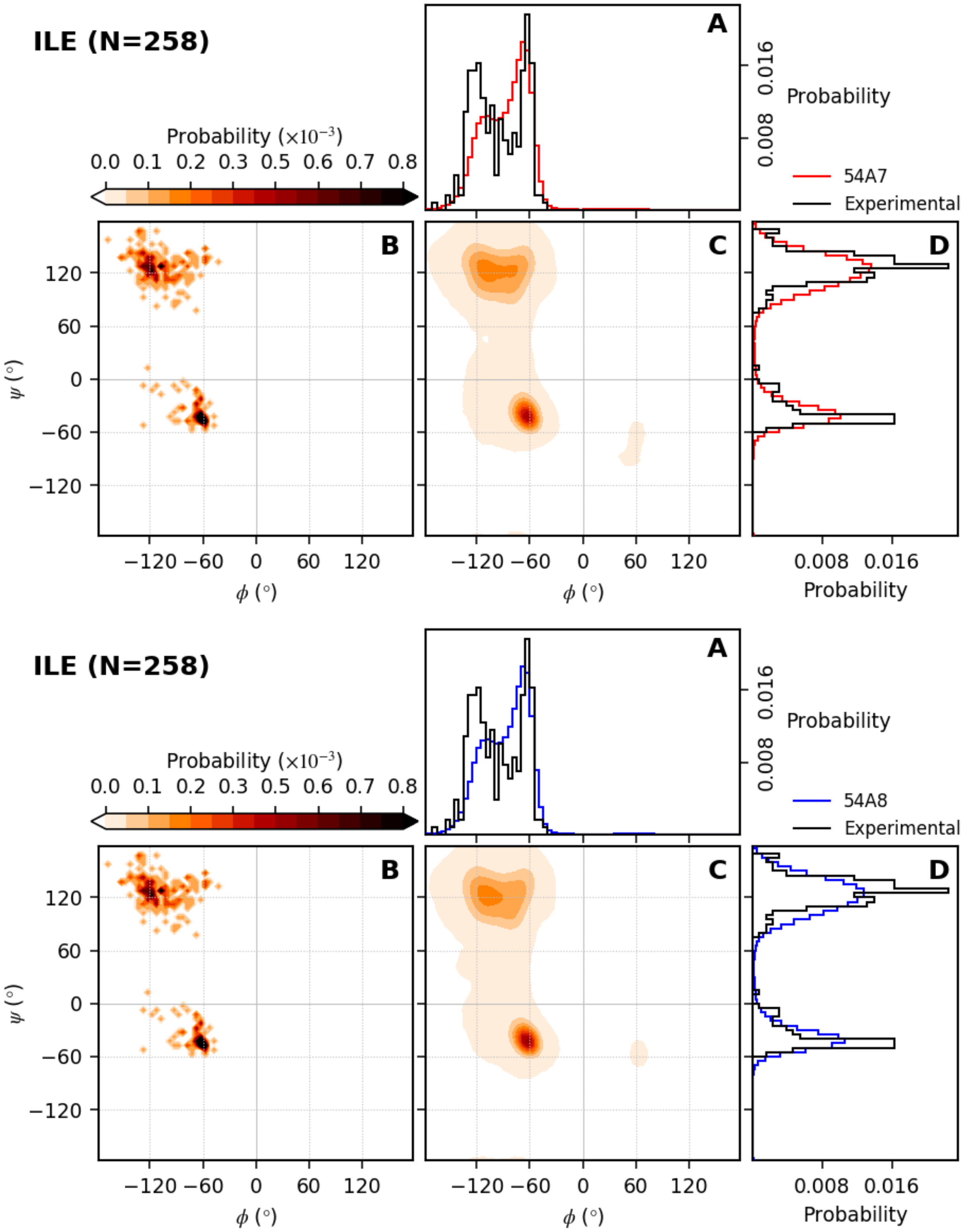

Figure S15. Distribution of isoleucine (ILE) backbone dihedral angles.

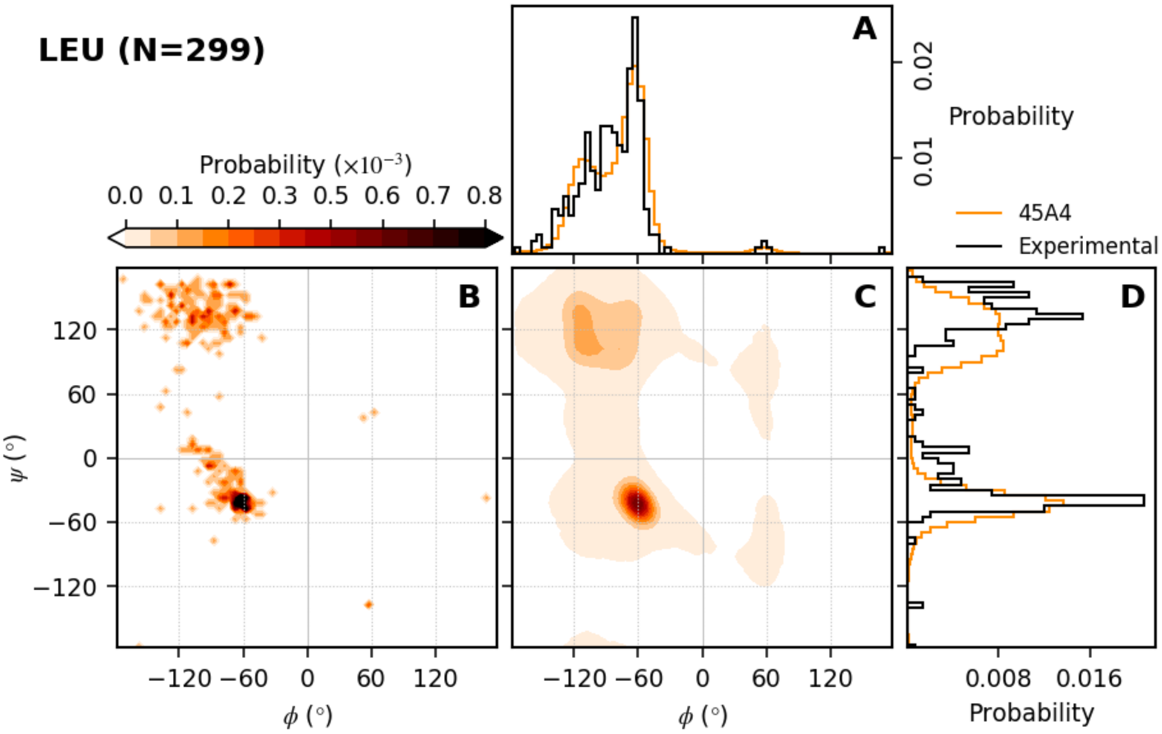
LEU (N=299)
Probability (×10⁻³)
0.0 0.1 0.2 0.3 0.5 0.6 0.7 0.8
A
B
C
D
Probability
45A4
Experimental
ψ (°)
ϕ (°)
Probability

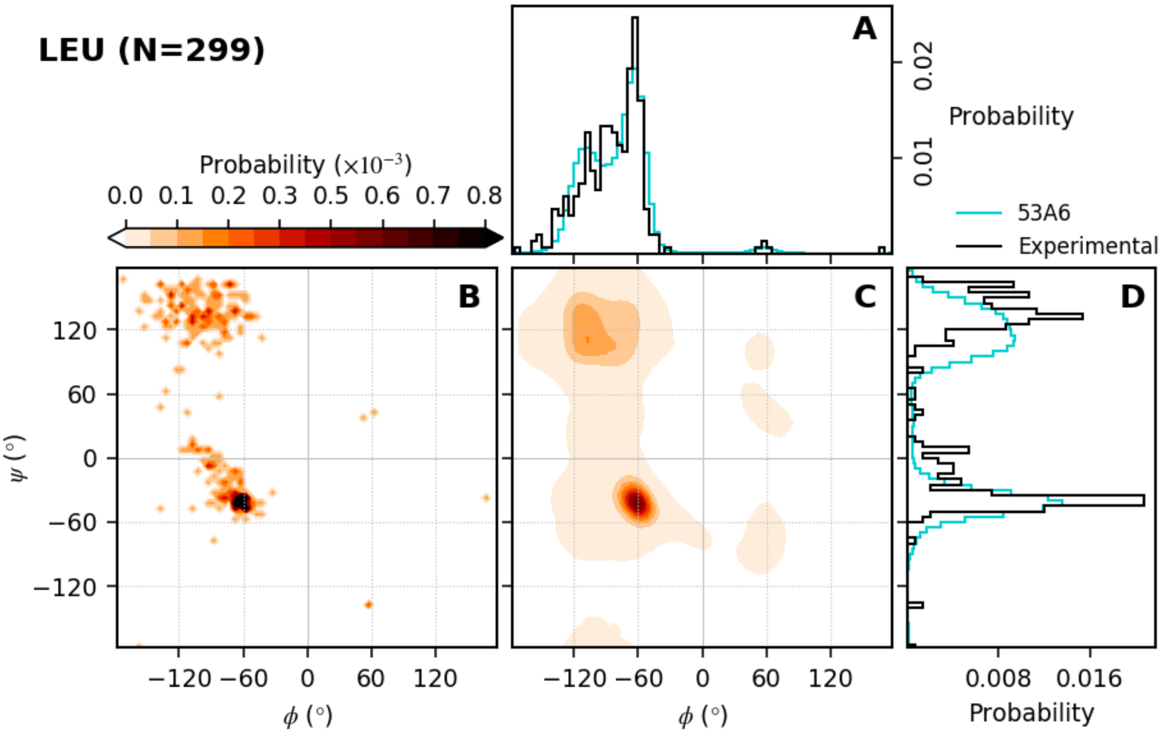
LEU (N=299)
Probability (×10⁻³)
0.0 0.1 0.2 0.3 0.5 0.6 0.7 0.8
A
B
C
D
Probability
53A6
Experimental
ψ (°)
ϕ (°)
Probability

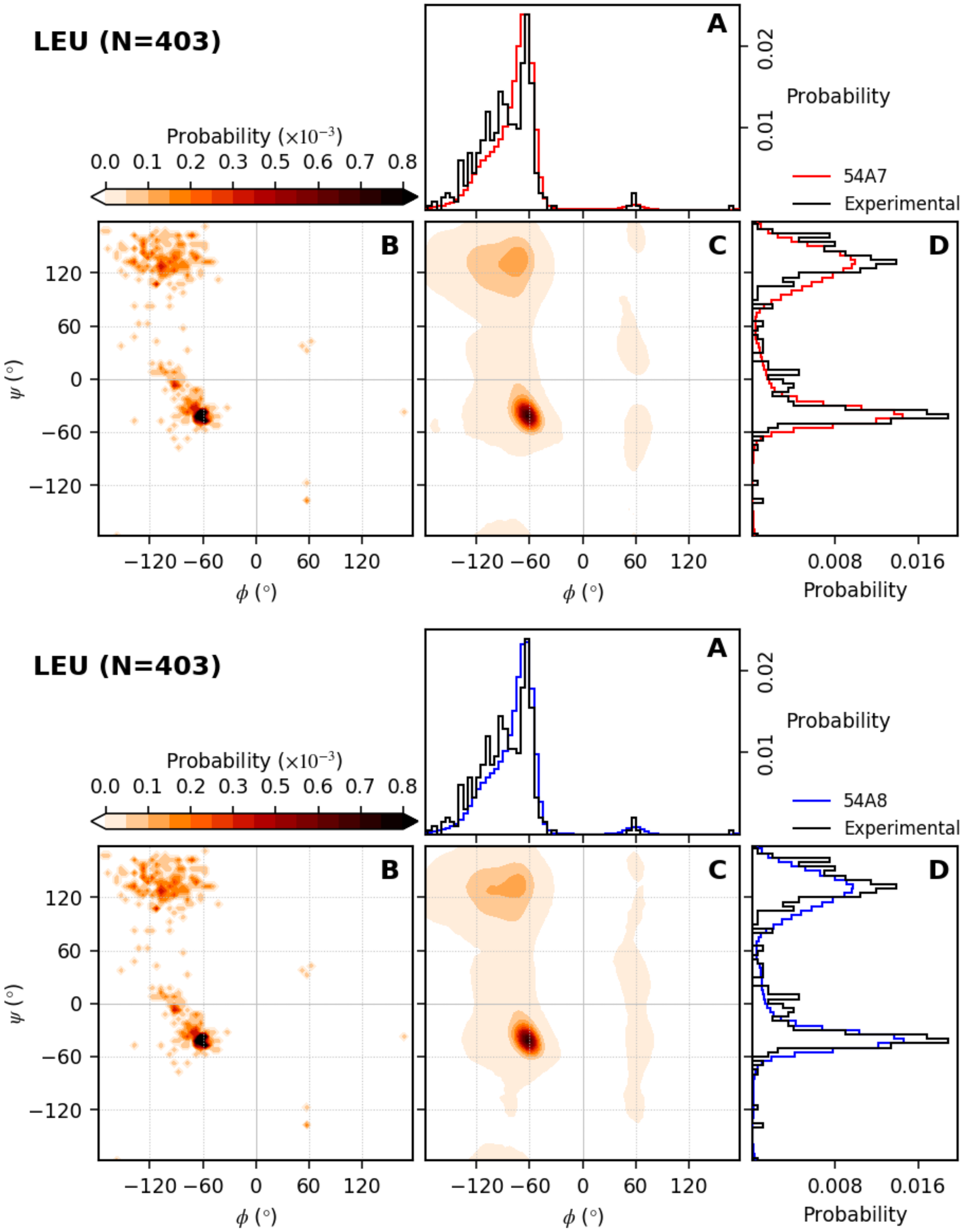

Figure S16. Distribution of leucine (LEU) backbone dihedral angles.

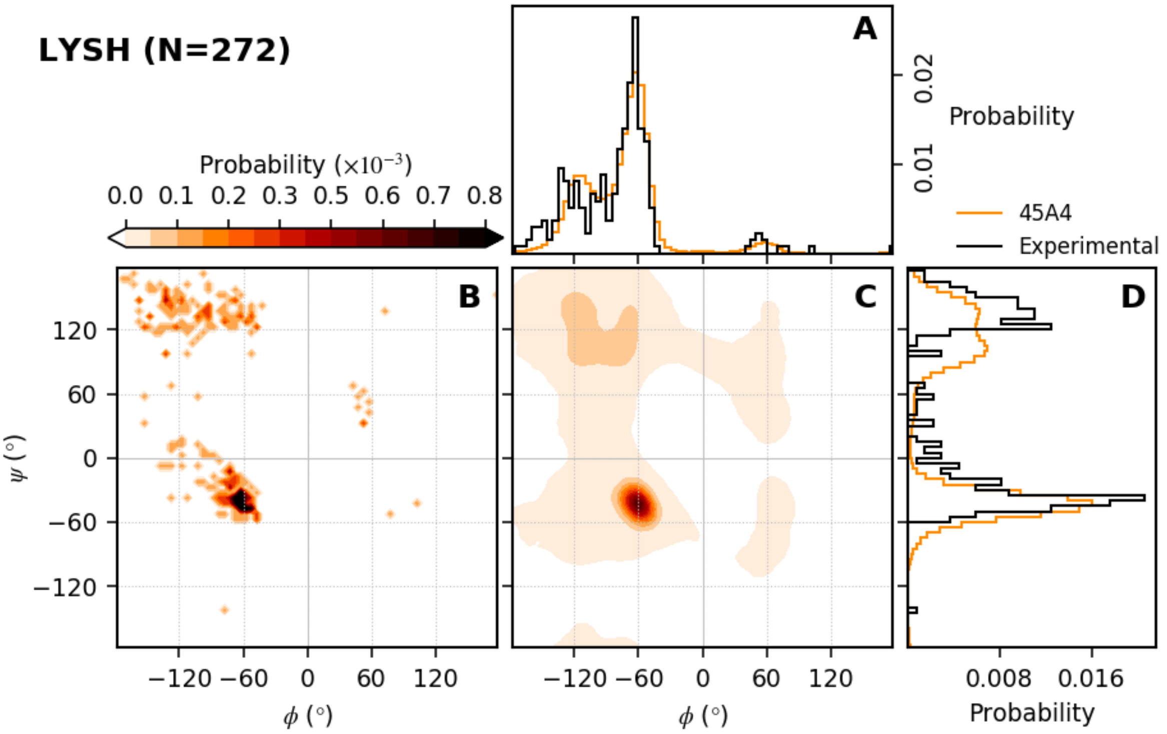
LYSH (N=272)
Probability (×10⁻³)
0.0 0.1 0.2 0.3 0.5 0.6 0.7 0.8
A
B
C
D
Probability
45A4
Experimental
ψ (°)
ϕ (°)
ϕ (°)
Probability

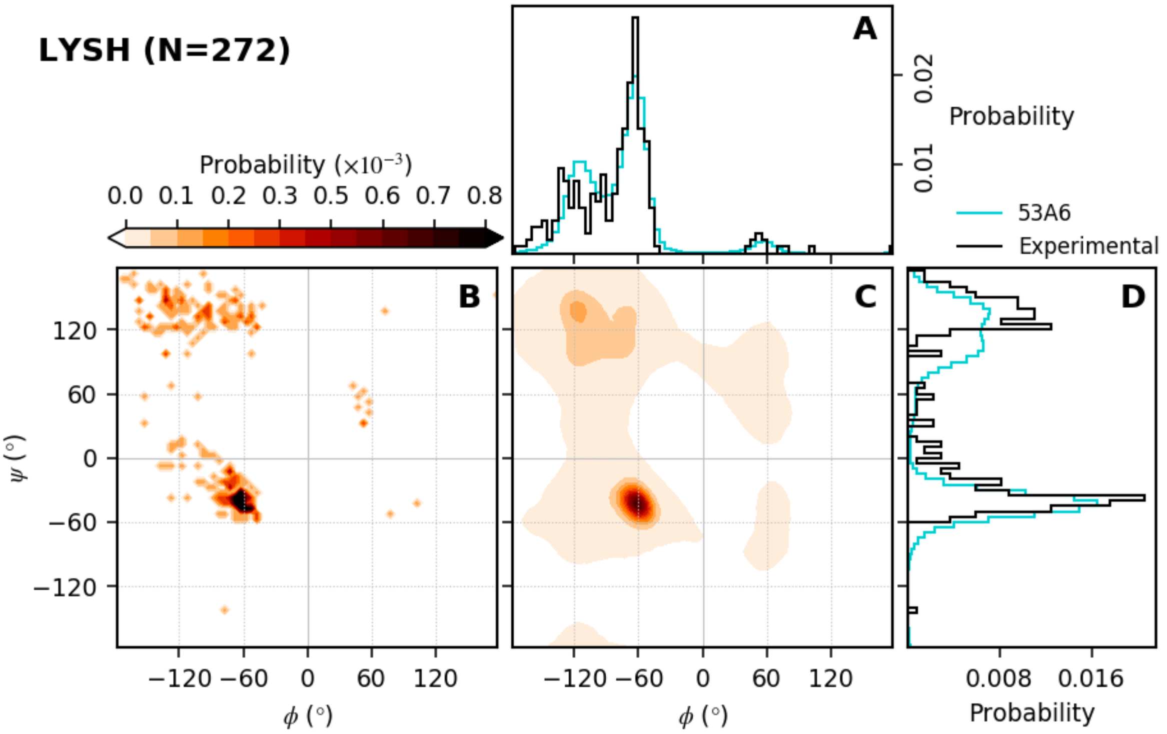
LYSH (N=272)
Probability (×10⁻³)
0.0 0.1 0.2 0.3 0.5 0.6 0.7 0.8
A
B
C
D
Probability
53A6
Experimental
ψ (°)
ϕ (°)
ϕ (°)
Probability

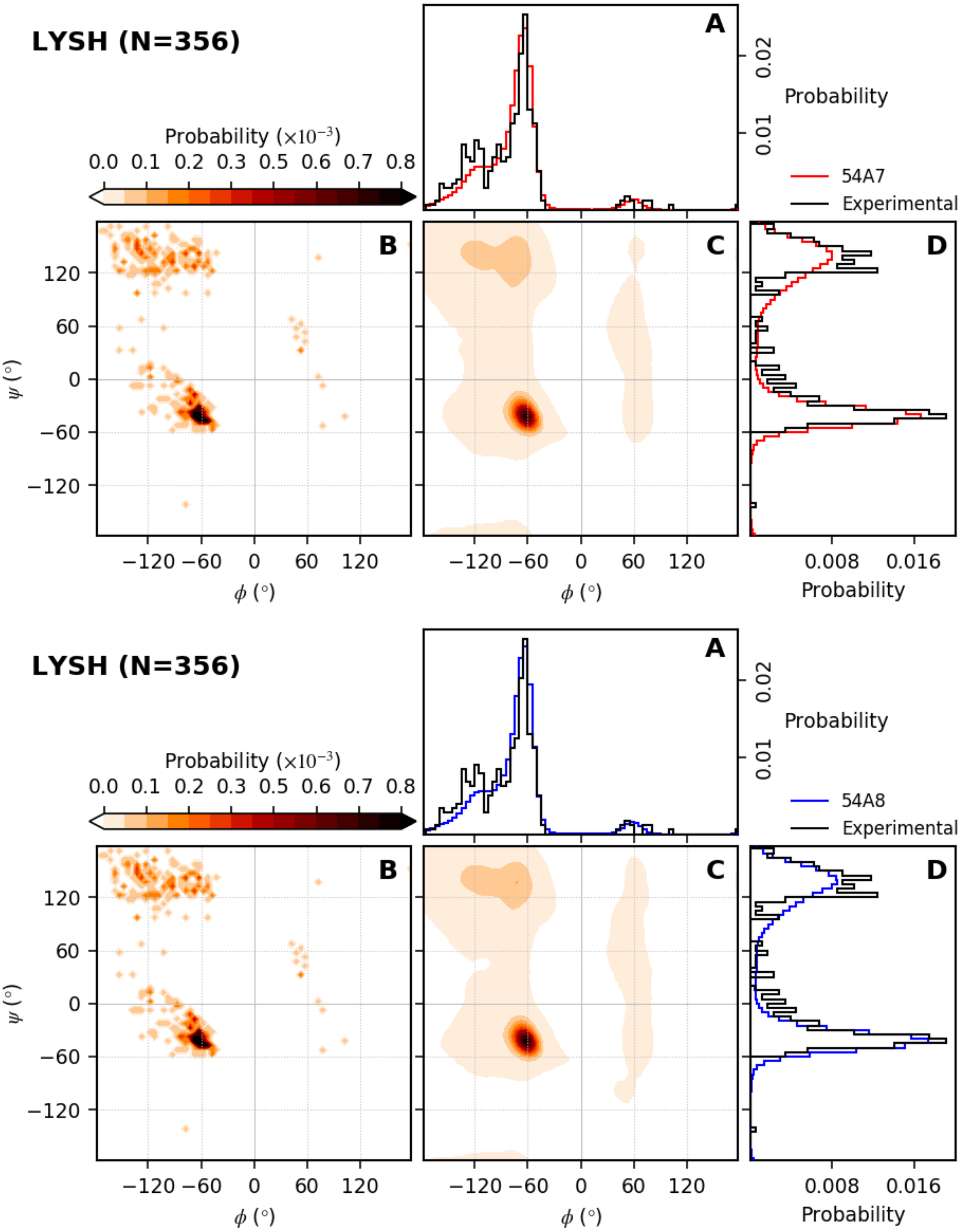

Figure S17. Distribution of lysine with +1 formal charge (LYSH) backbone dihedral angles.

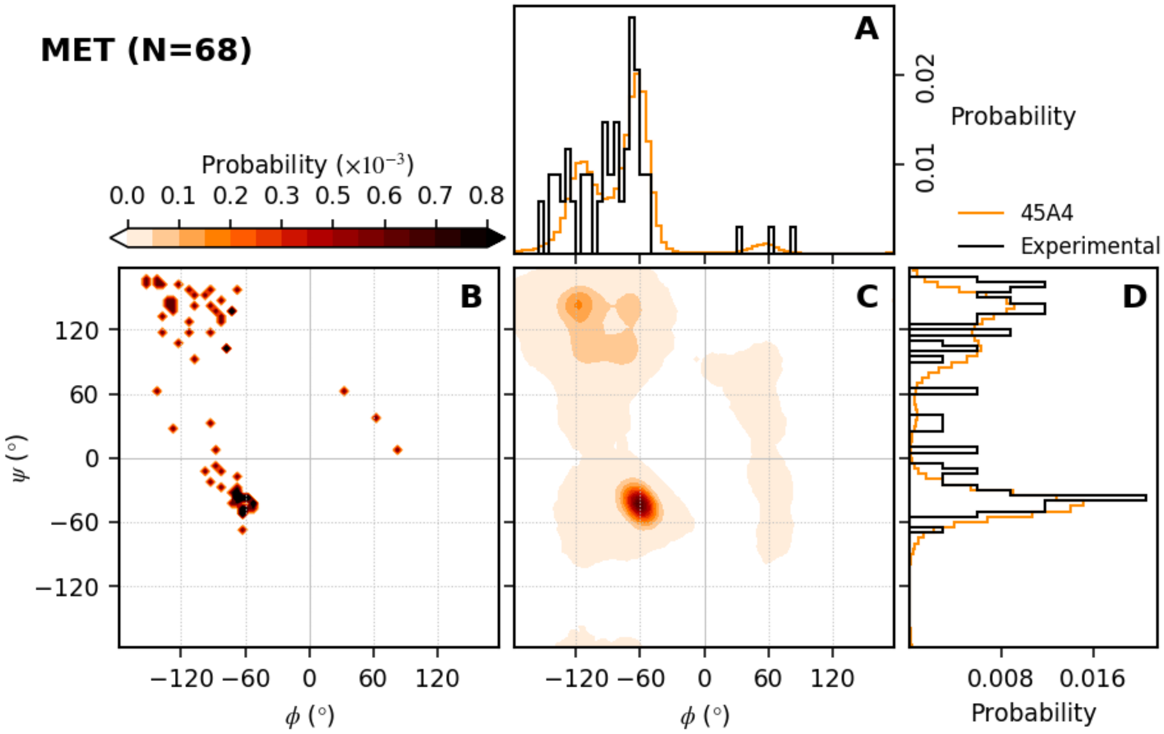
MET (N=68)
Probability (×10⁻³)
0.0 0.1 0.2 0.3 0.5 0.6 0.7 0.8
A
B
C
D
Probability
45A4
Experimental
ψ (°)
ϕ (°)
Probability

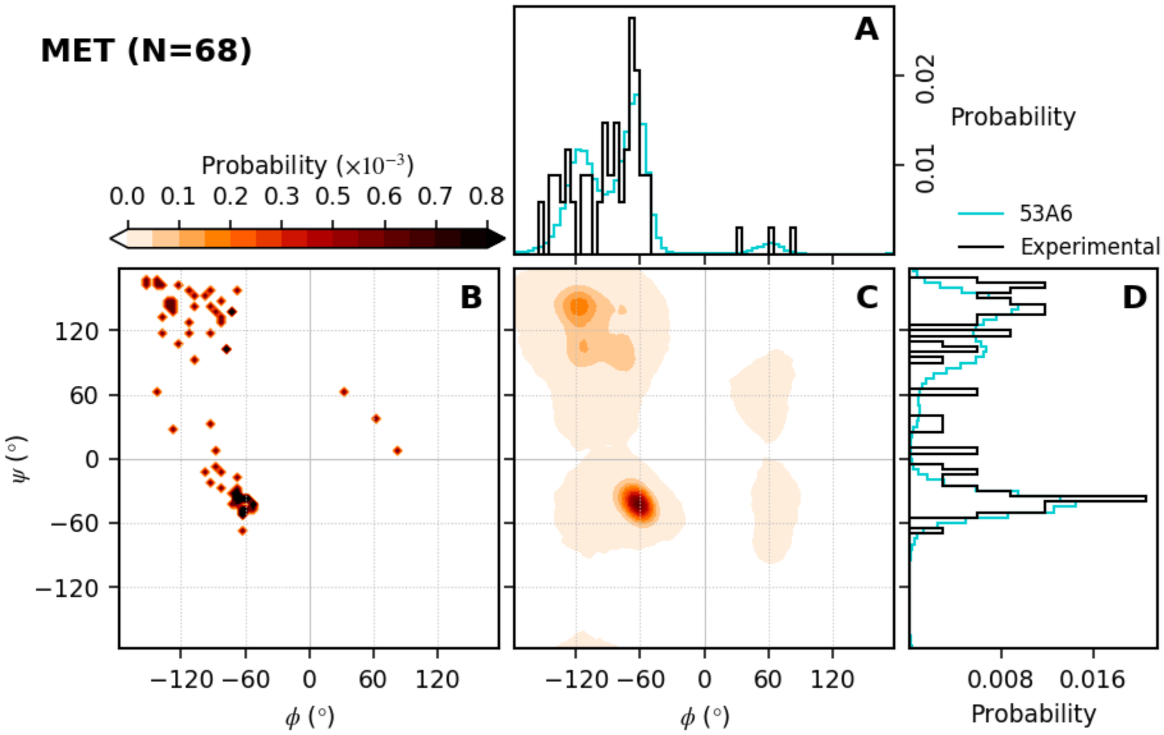
MET (N=68)
Probability (×10⁻³)
0.0 0.1 0.2 0.3 0.5 0.6 0.7 0.8
A
B
C
D
Probability
53A6
Experimental
ψ (°)
ϕ (°)
Probability

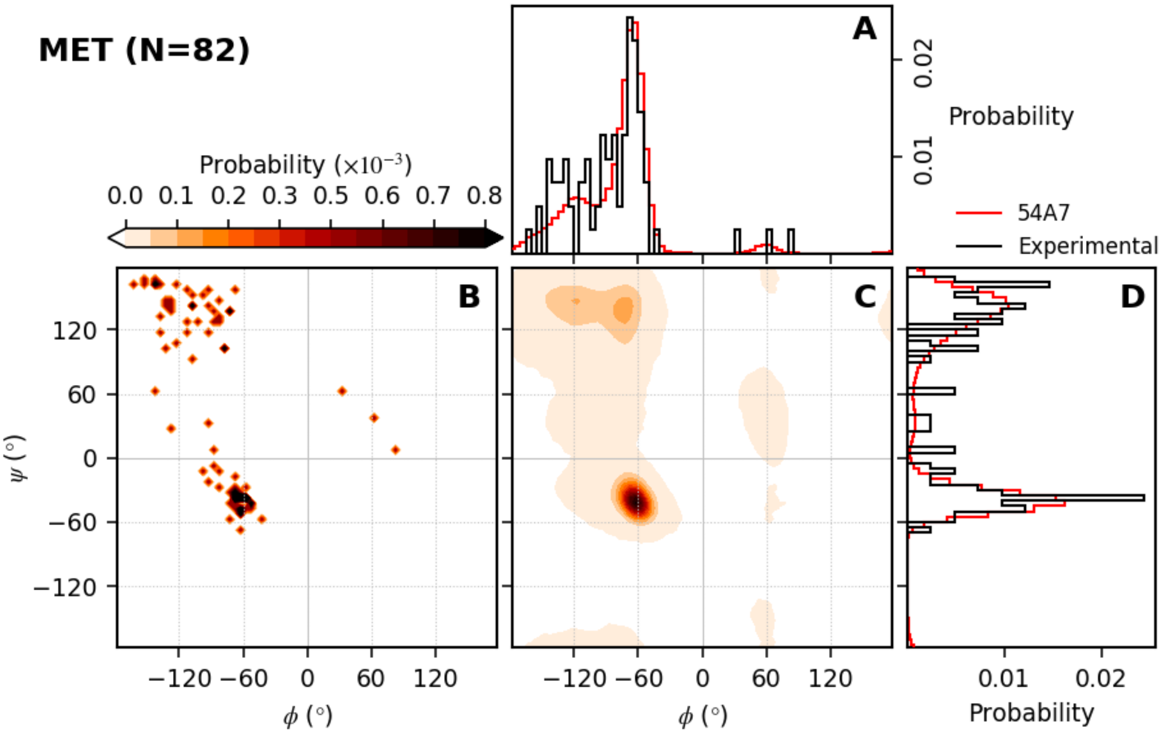

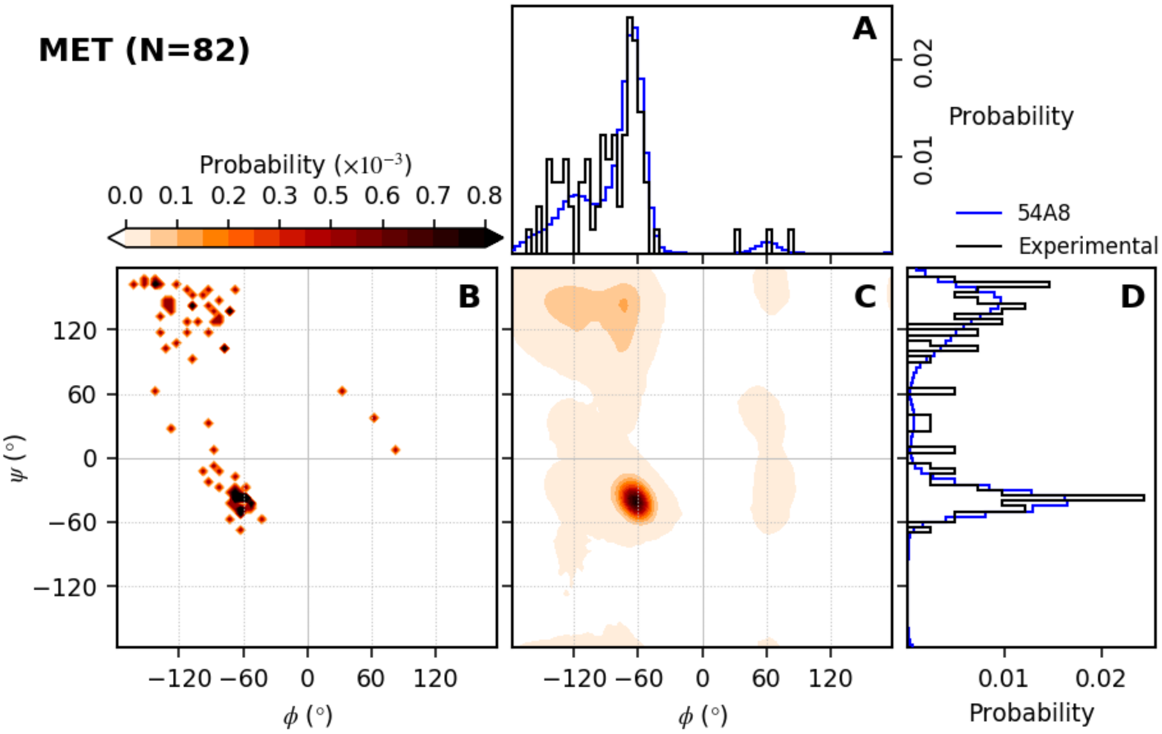

Figure S18. Distribution of methionine (MET) backbone dihedral angles.

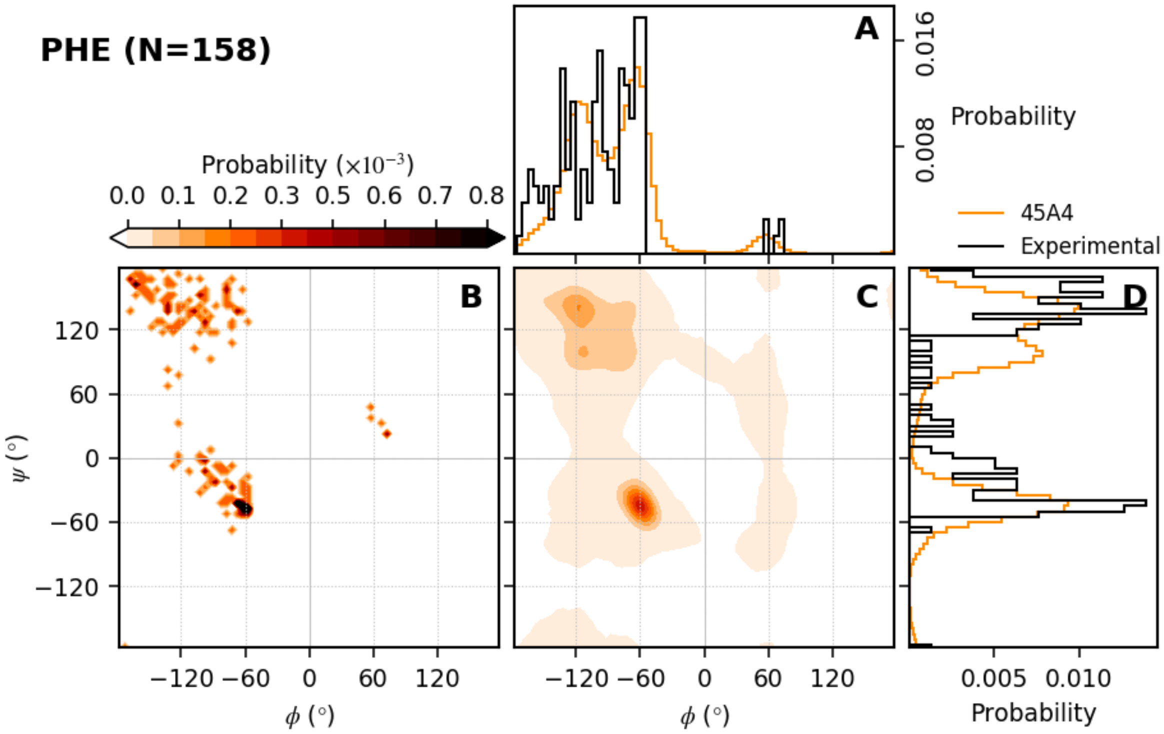
PHE (N=158)
Probability (×10⁻³)
0.0 0.1 0.2 0.3 0.5 0.6 0.7 0.8
A
B
C
D
Probability
0.008
0.016
45A4
Experimental
120
60
0
−60
−120
ψ (°)
−120 −60 0 60 120
ϕ (°)
0.005 0.010
Probability

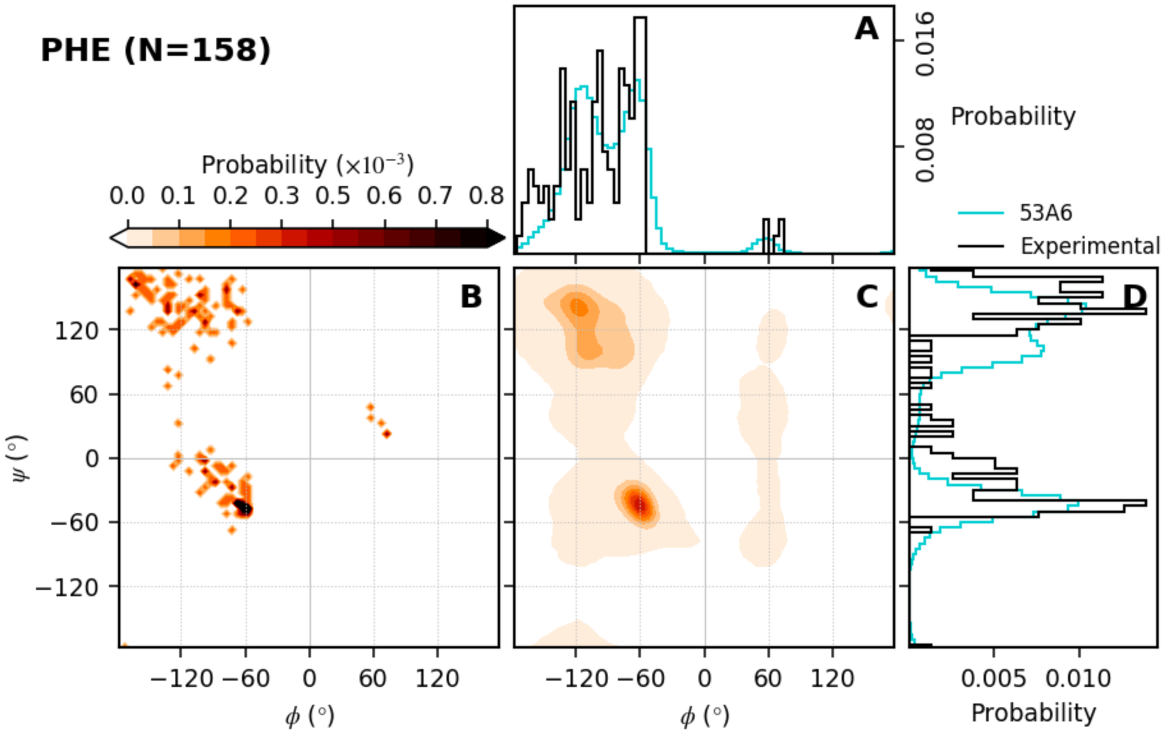
PHE (N=158)
Probability (×10⁻³)
0.0 0.1 0.2 0.3 0.5 0.6 0.7 0.8
A
B
C
D
Probability
0.008
0.016
53A6
Experimental
120
60
0
−60
−120
ψ (°)
−120 −60 0 60 120
ϕ (°)
0.005 0.010
Probability

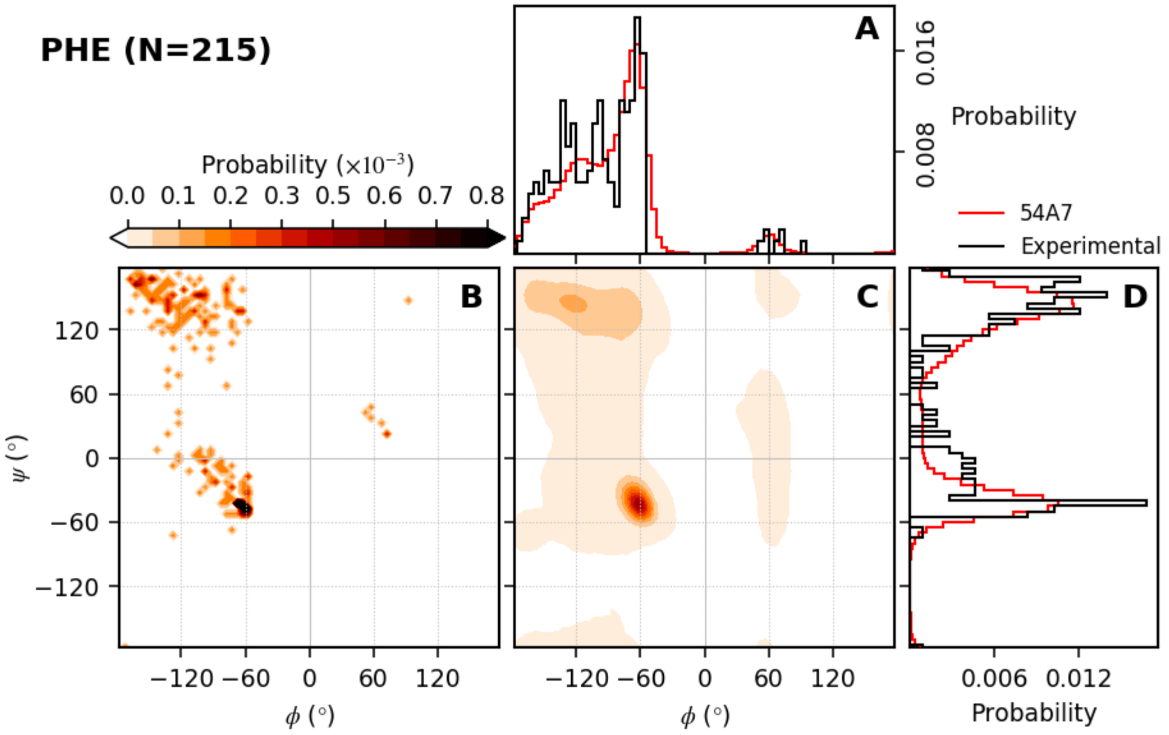

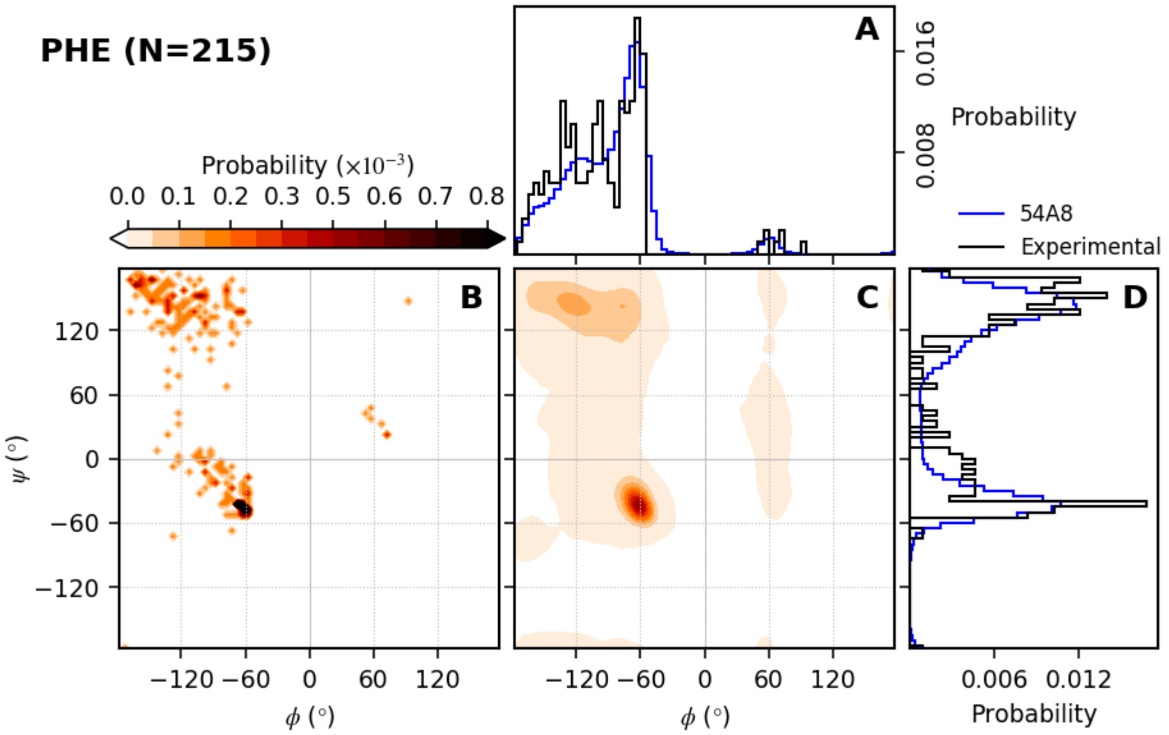

Figure S19. Distribution of phenylalanine (PHE) backbone dihedral angles.

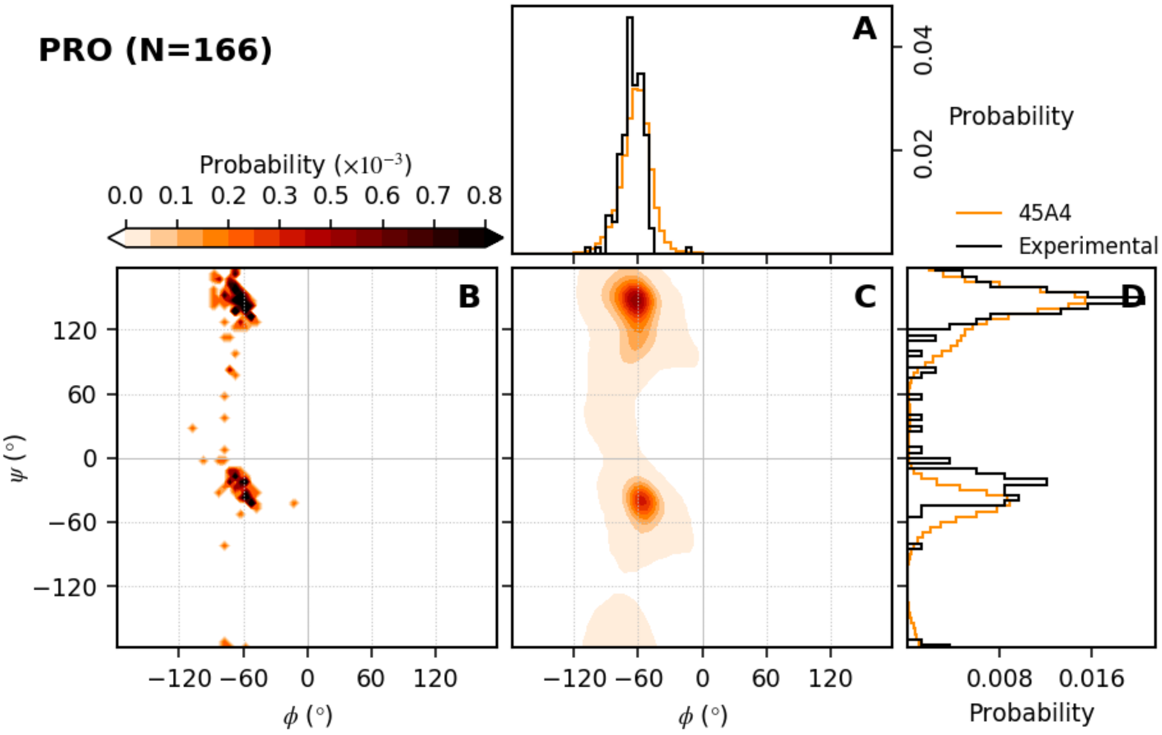
PRO (N=166)
Probability (×10⁻³)
0.0 0.1 0.2 0.3 0.5 0.6 0.7 0.8
A
B
C
D
Probability
45A4
Experimental
ψ (°)
ϕ (°)

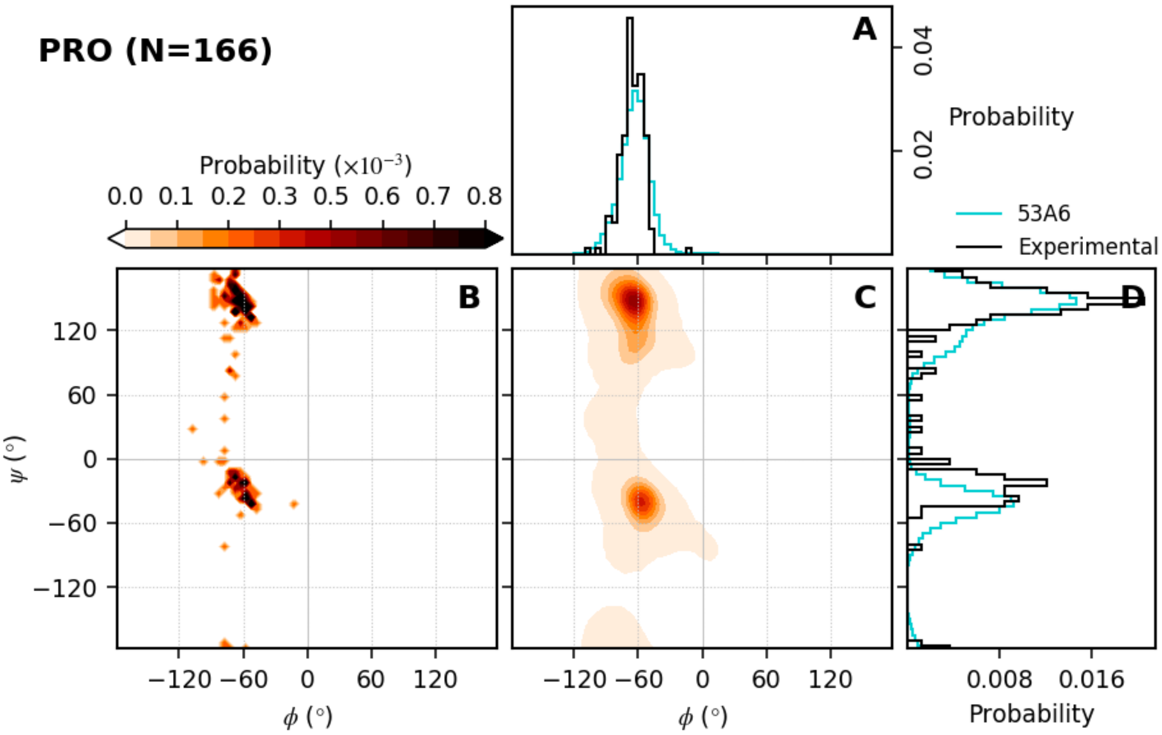
PRO (N=166)
Probability (×10⁻³)
0.0 0.1 0.2 0.3 0.5 0.6 0.7 0.8
A
B
C
D
Probability
53A6
Experimental
ψ (°)
ϕ (°)

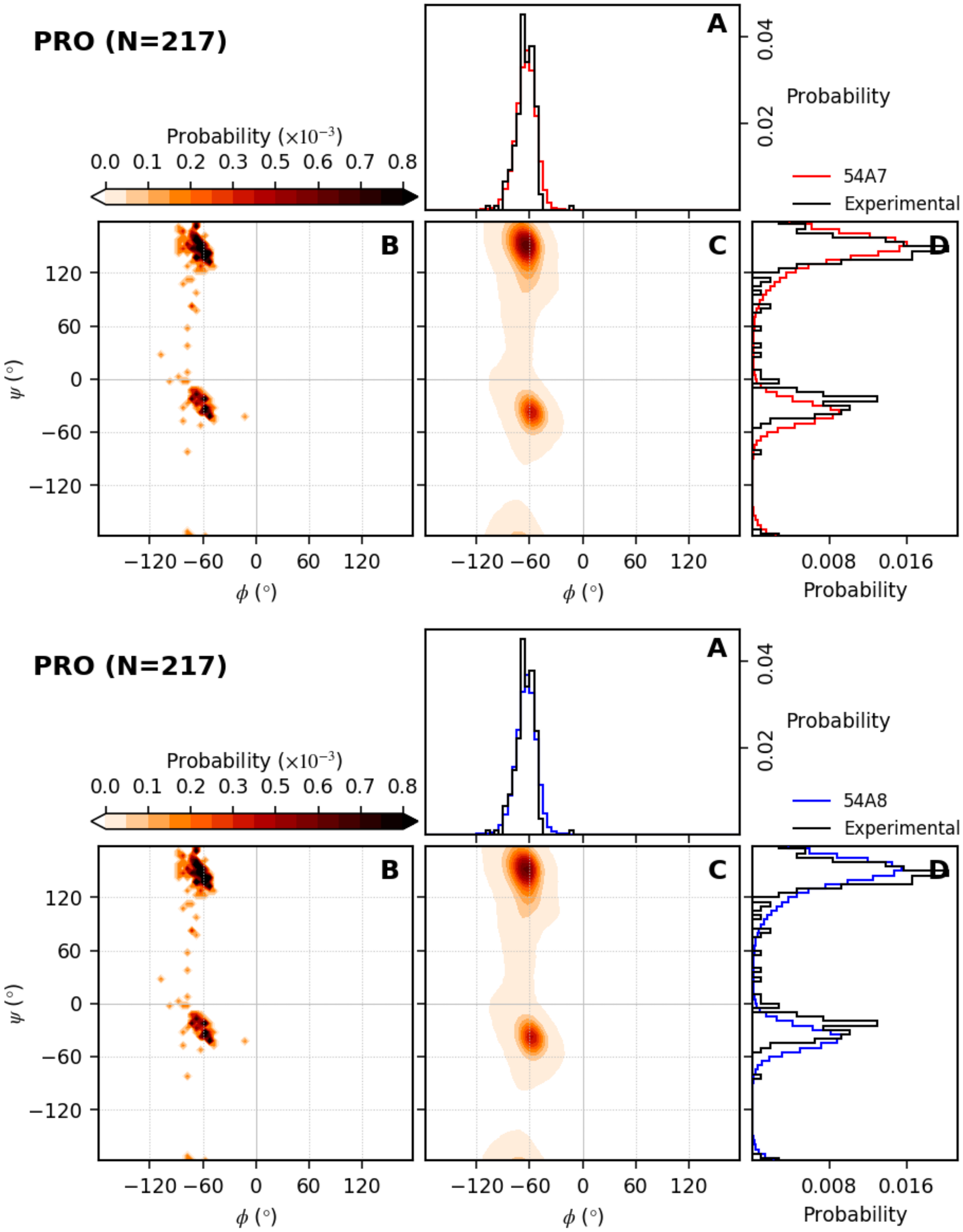

Figure S20. Distribution of proline (PRO) backbone dihedral angles.

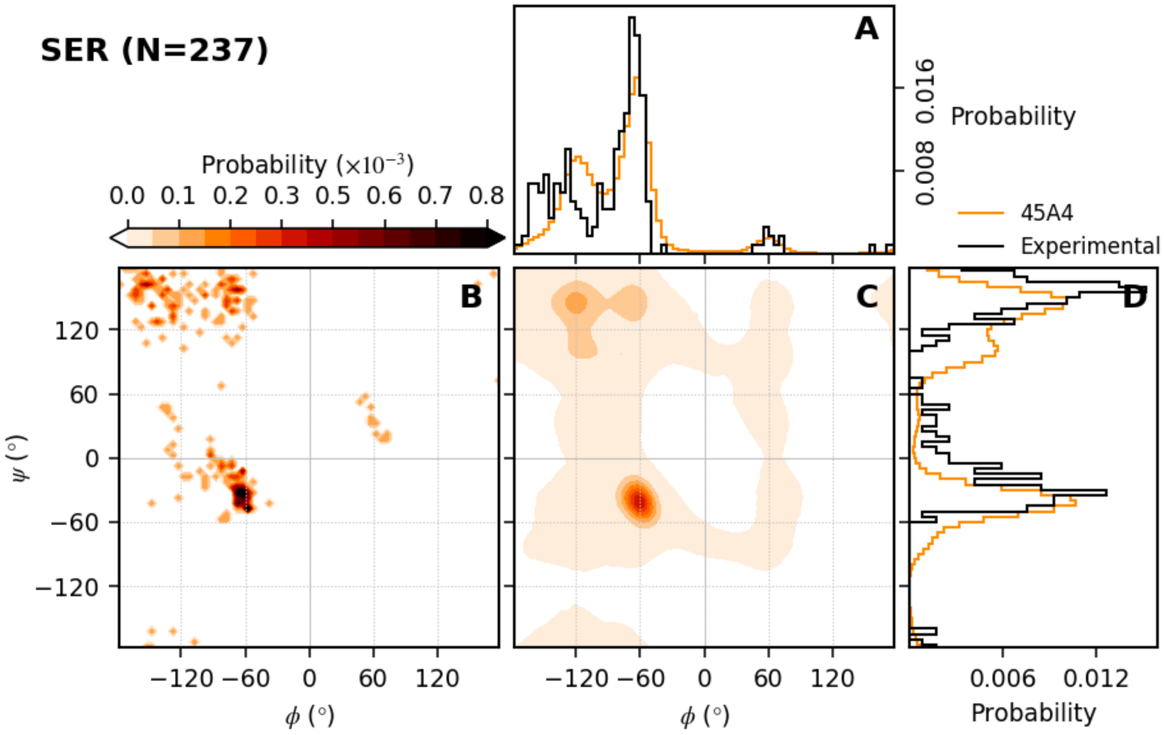
SER (N=237)
Probability (×10⁻³)
0.0 0.1 0.2 0.3 0.5 0.6 0.7 0.8
A
B
C
D
Probability
0.008 0.016
45A4
Experimental
120
60
0
−60
−120
ψ (°)
−120 −60 0 60 120
ϕ (°)
0.006 0.012
Probability

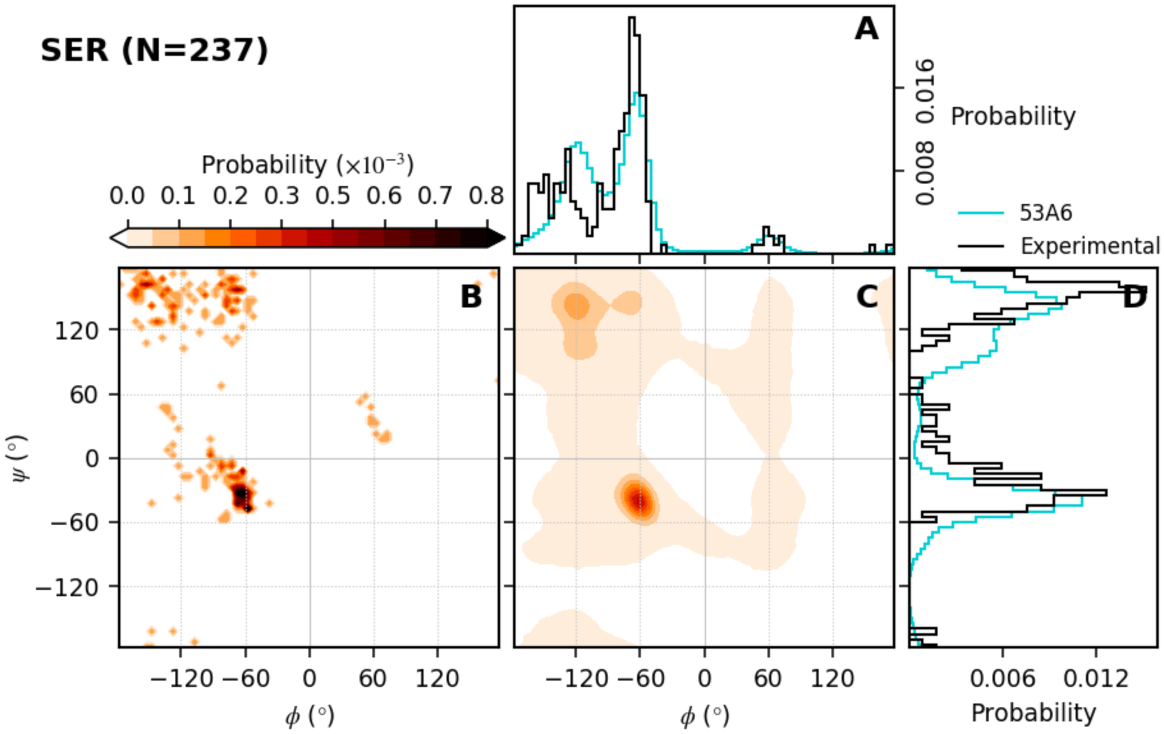
SER (N=237)
Probability (×10⁻³)
0.0 0.1 0.2 0.3 0.5 0.6 0.7 0.8
A
B
C
D
Probability
0.008 0.016
53A6
Experimental
120
60
0
−60
−120
ψ (°)
−120 −60 0 60 120
ϕ (°)
0.006 0.012
Probability

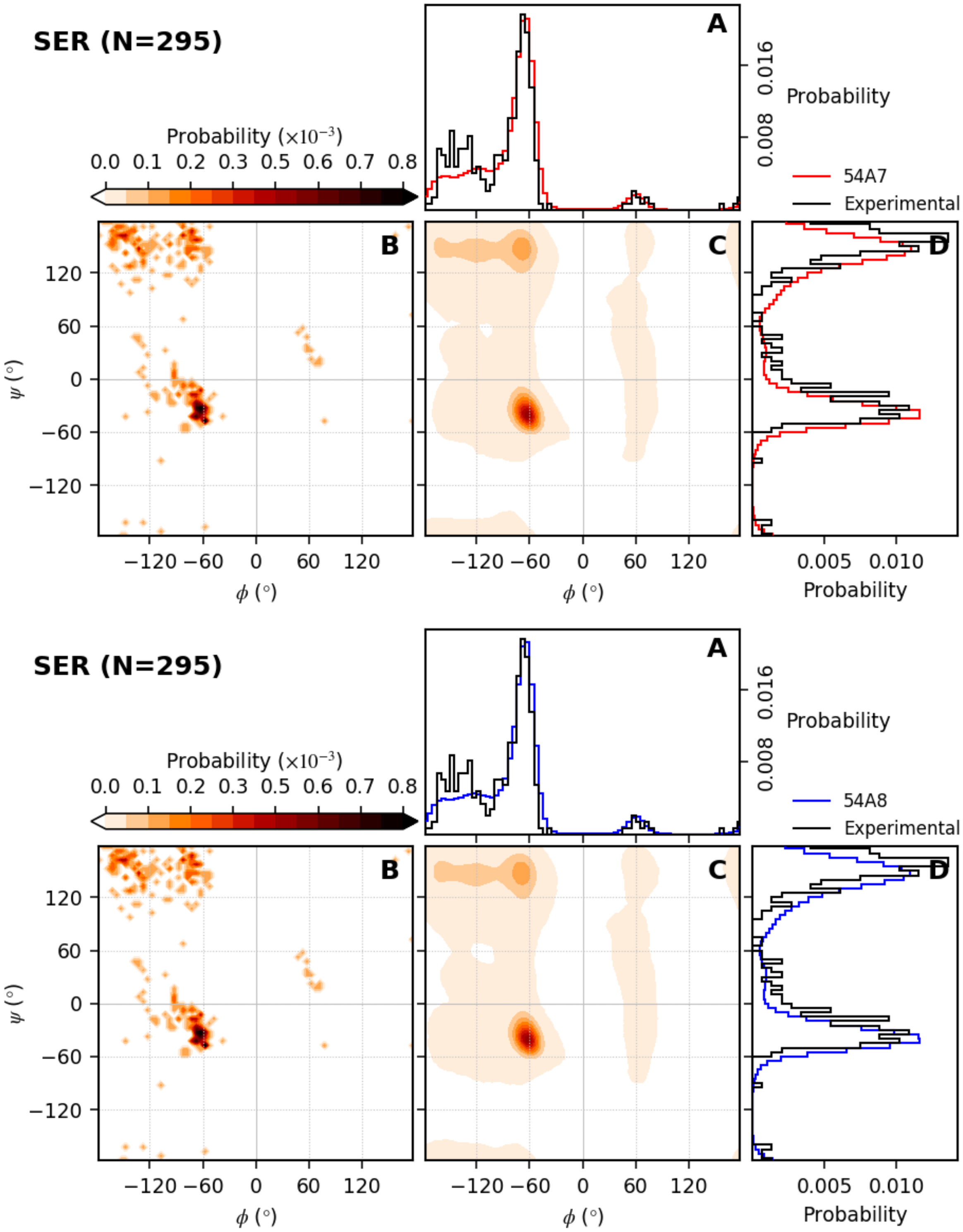

Figure S21. Distribution of serine (SER) backbone dihedral angles.

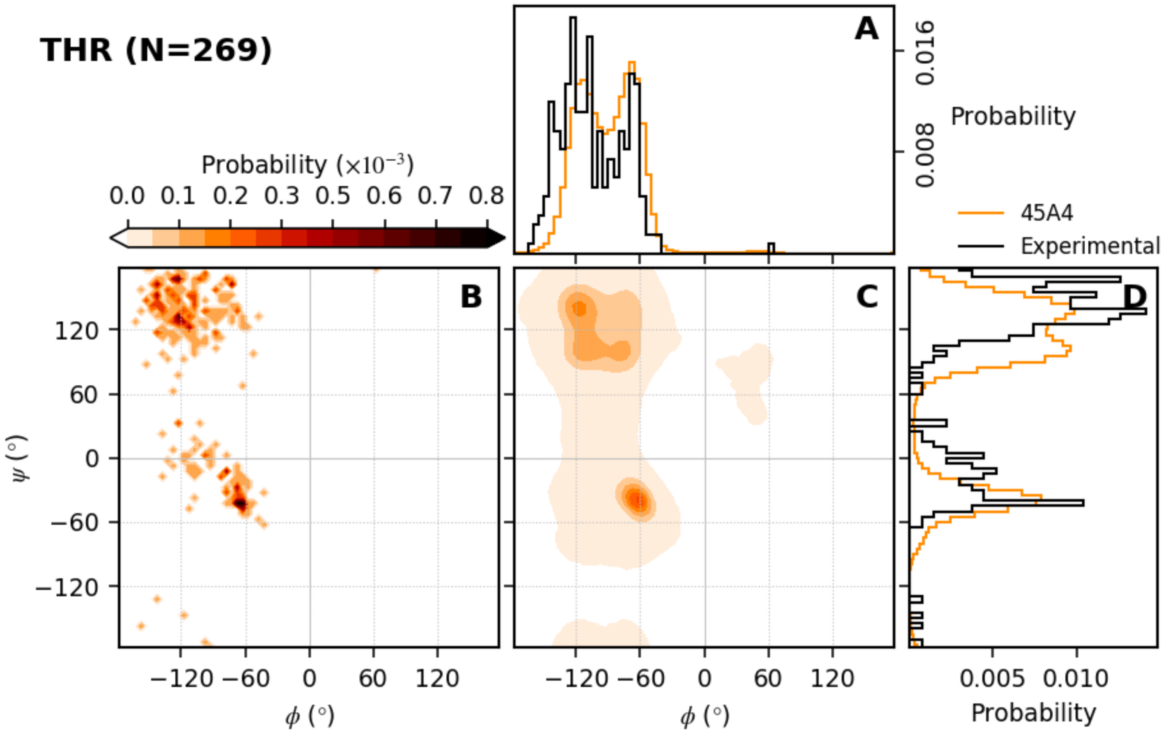
THR (N=269)
Probability (×10⁻³)
0.0 0.1 0.2 0.3 0.5 0.6 0.7 0.8
A
B
C
D
Probability
45A4
Experimental
ψ (°)
ϕ (°)
0.005 0.010
0.016
0.008

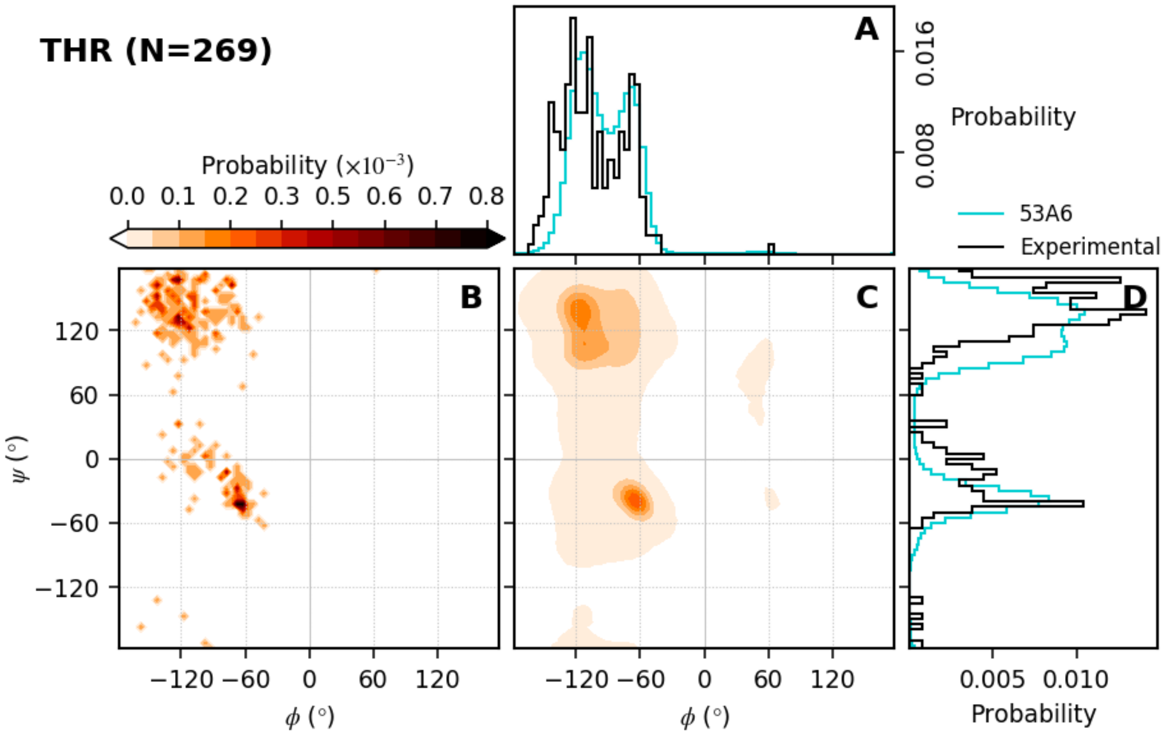
THR (N=269)
Probability (×10⁻³)
0.0 0.1 0.2 0.3 0.5 0.6 0.7 0.8
A
B
C
D
Probability
53A6
Experimental
ψ (°)
ϕ (°)
0.005 0.010
0.016
0.008

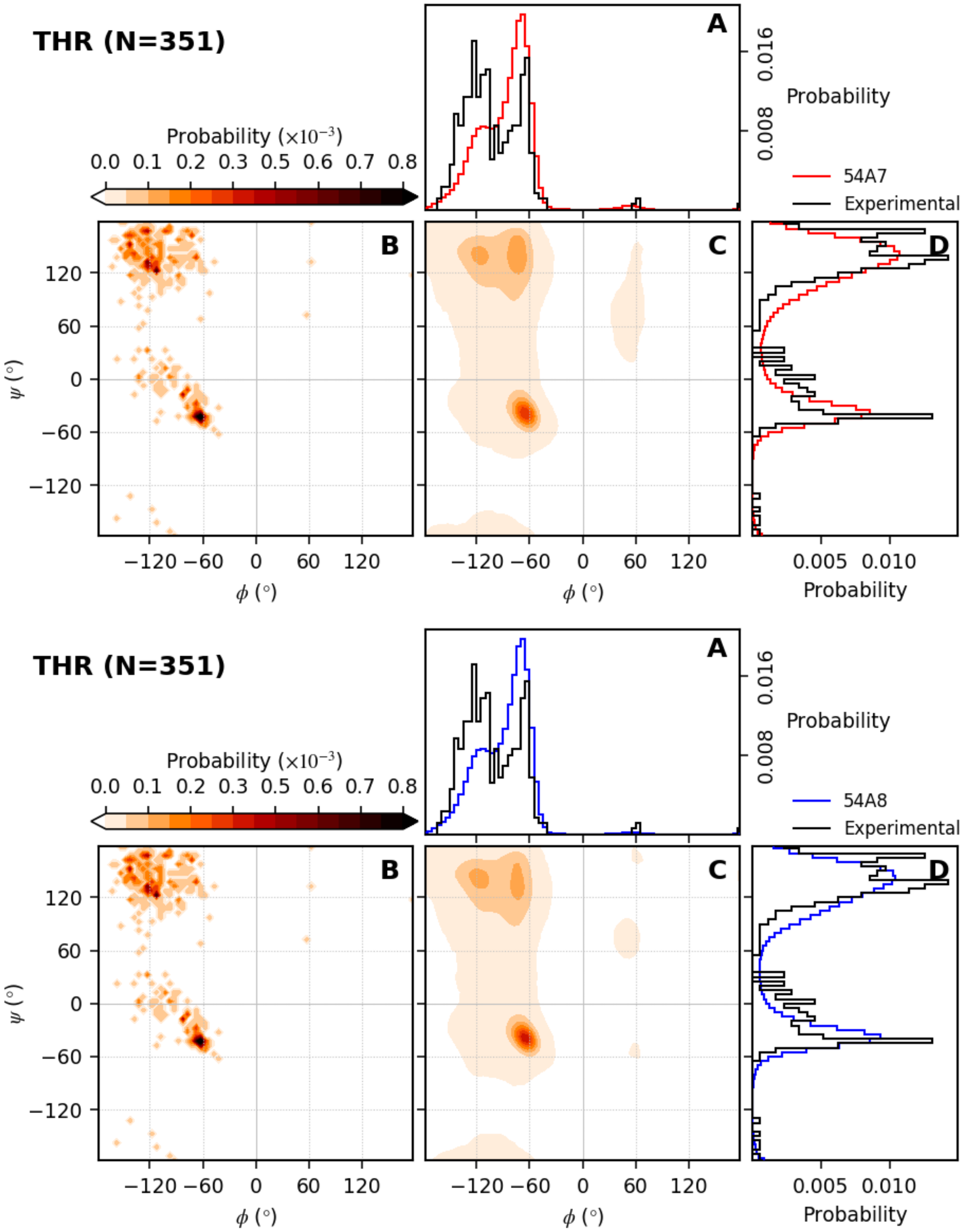

Figure S22. Distribution of threonine (THR) backbone dihedral angles.

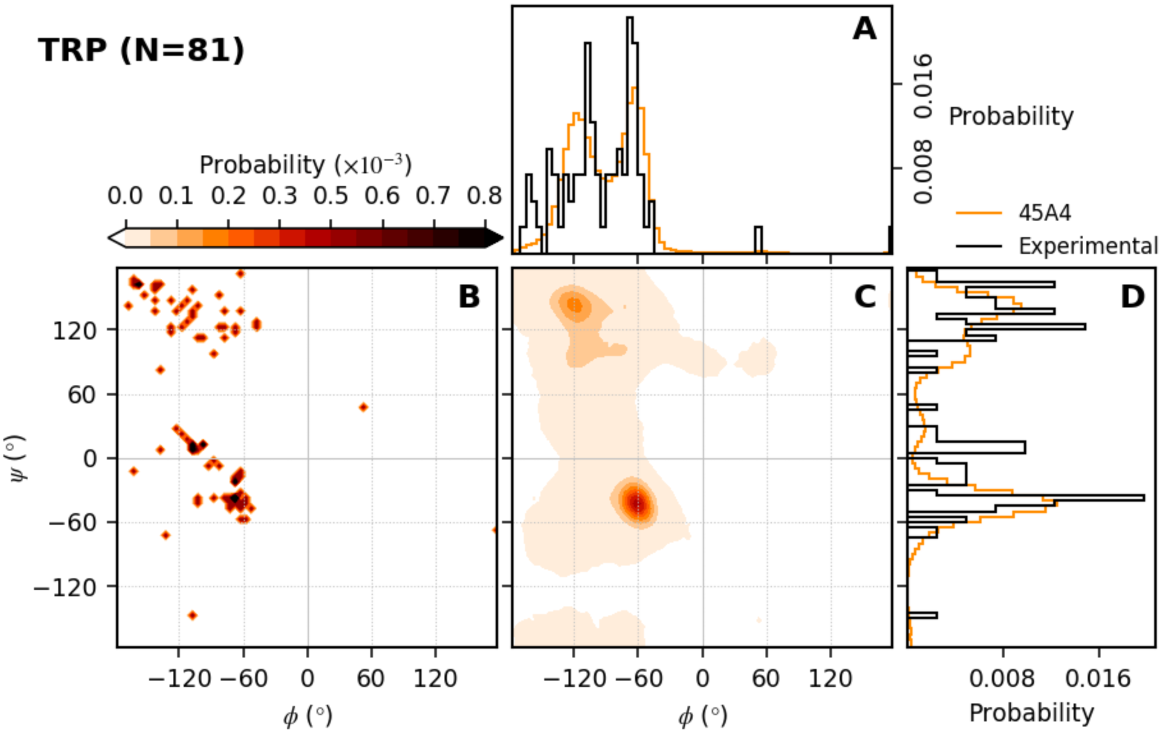

TRP (N=81)
Probability (×10⁻³)
0.0 0.1 0.2 0.3 0.5 0.6 0.7 0.8
A
B
C
D
Probability
0.008 0.016
45A4
Experimental
ψ (°)
ϕ (°)
−120 −60 0 60 120

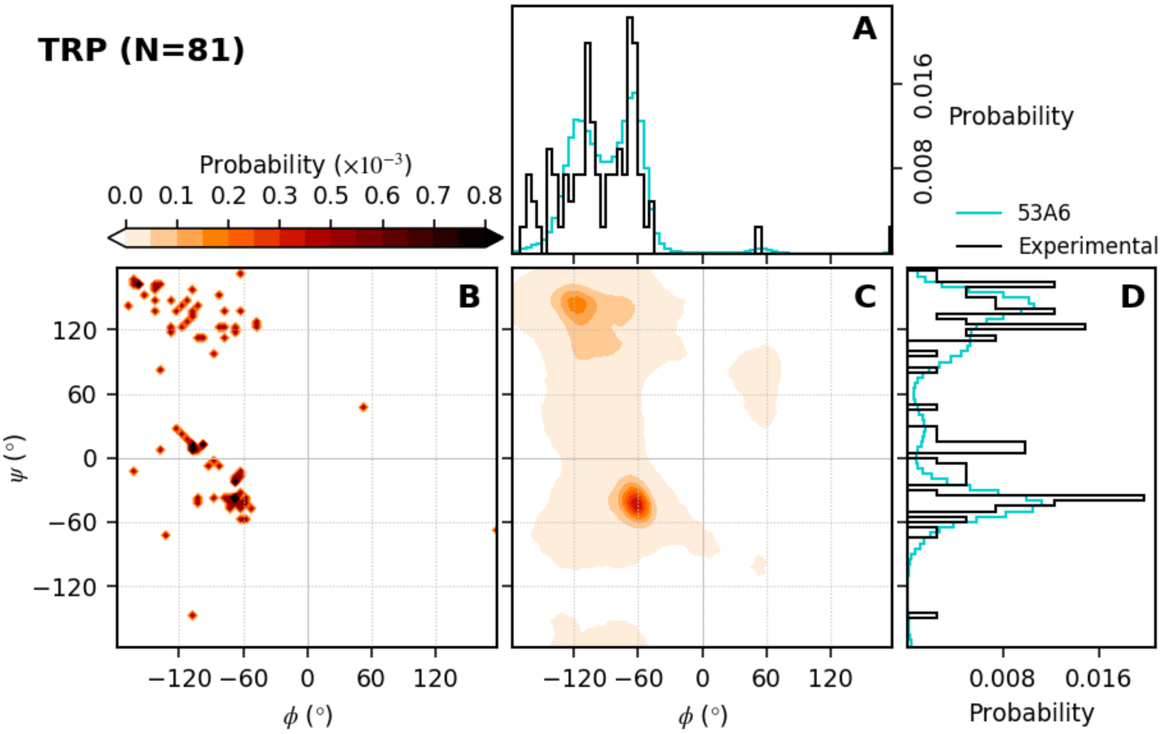

TRP (N=81)
Probability (×10⁻³)
0.0 0.1 0.2 0.3 0.5 0.6 0.7 0.8
A
B
C
D
Probability
0.008 0.016
53A6
Experimental
ψ (°)
ϕ (°)
−120 −60 0 60 120

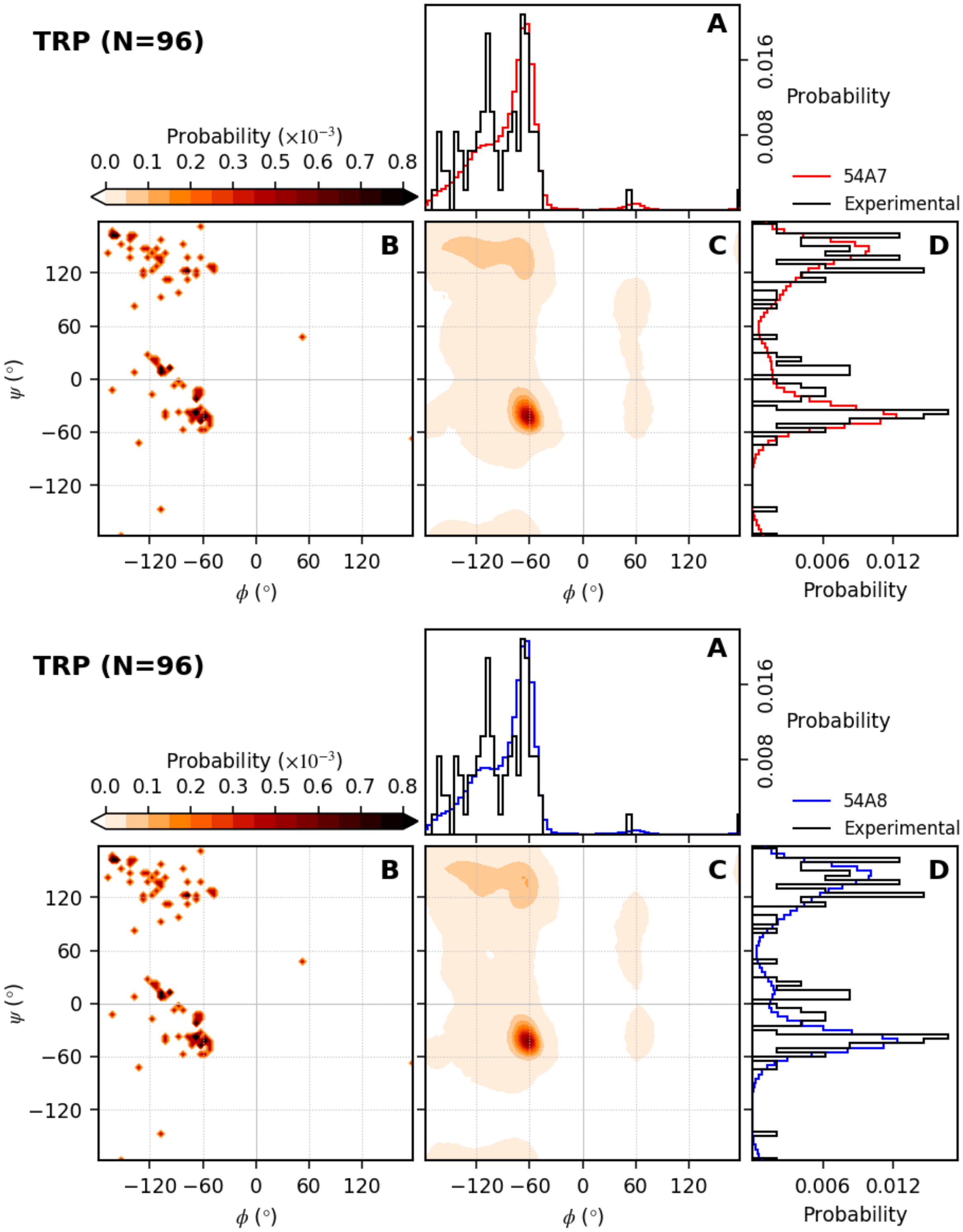

Figure S23. Distribution of tryptophan (TRP) backbone dihedral angles.

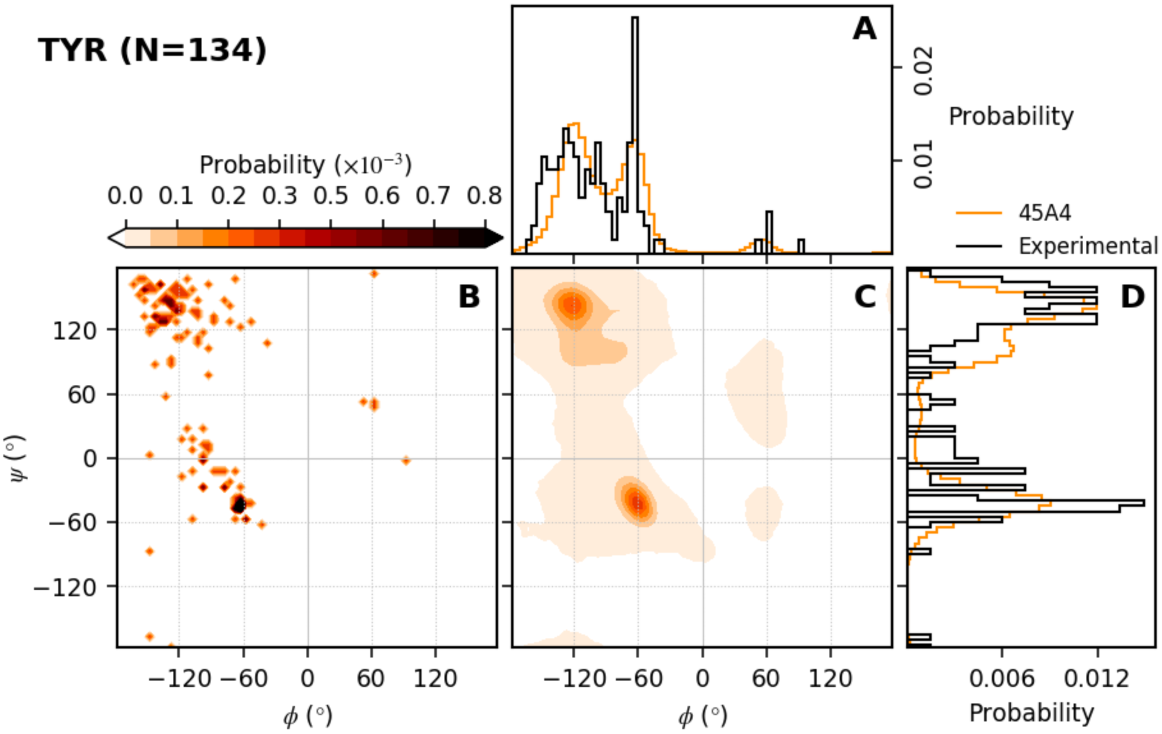
TYR (N=134)
A
B
C
D
Probability (×10⁻³)
0.0 0.1 0.2 0.3 0.5 0.6 0.7 0.8
Probability
0.01
0.02
45A4
Experimental
120
60
0
−60
−120
ψ (°)
−120 −60 0 60 120
ϕ (°)
0.006 0.012

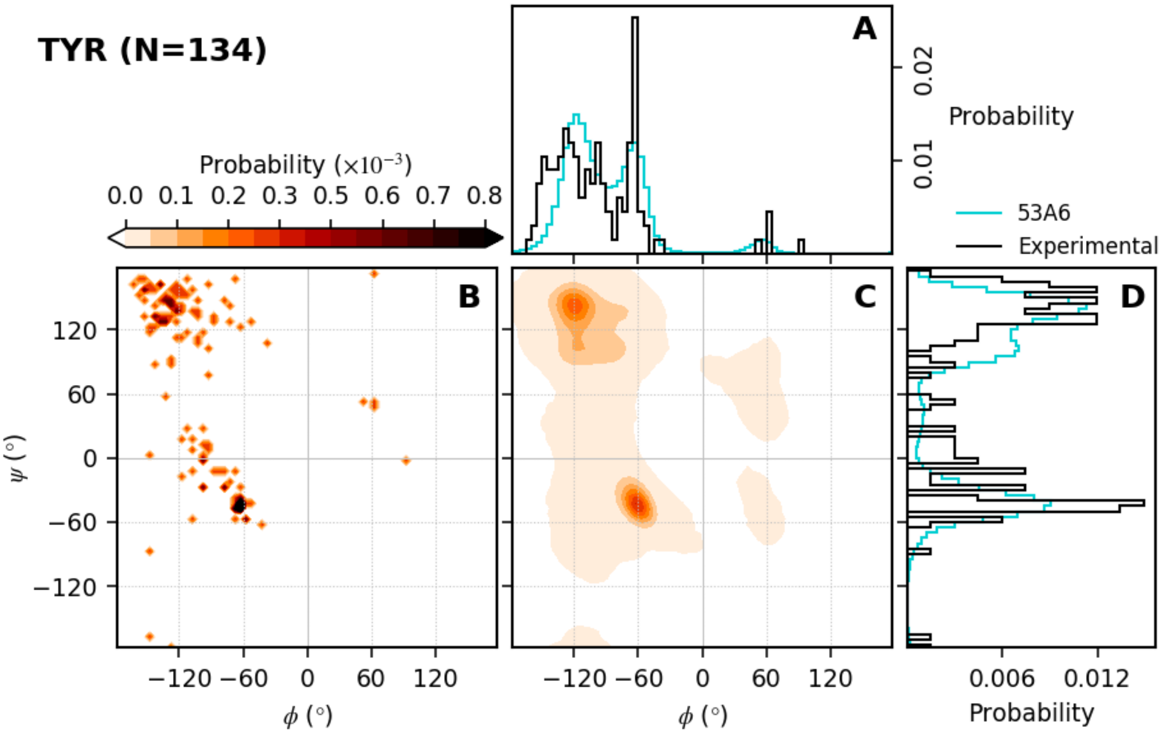
TYR (N=134)
A
B
C
D
Probability (×10⁻³)
0.0 0.1 0.2 0.3 0.5 0.6 0.7 0.8
Probability
0.01
0.02
53A6
Experimental
120
60
0
−60
−120
ψ (°)
−120 −60 0 60 120
ϕ (°)
0.006 0.012

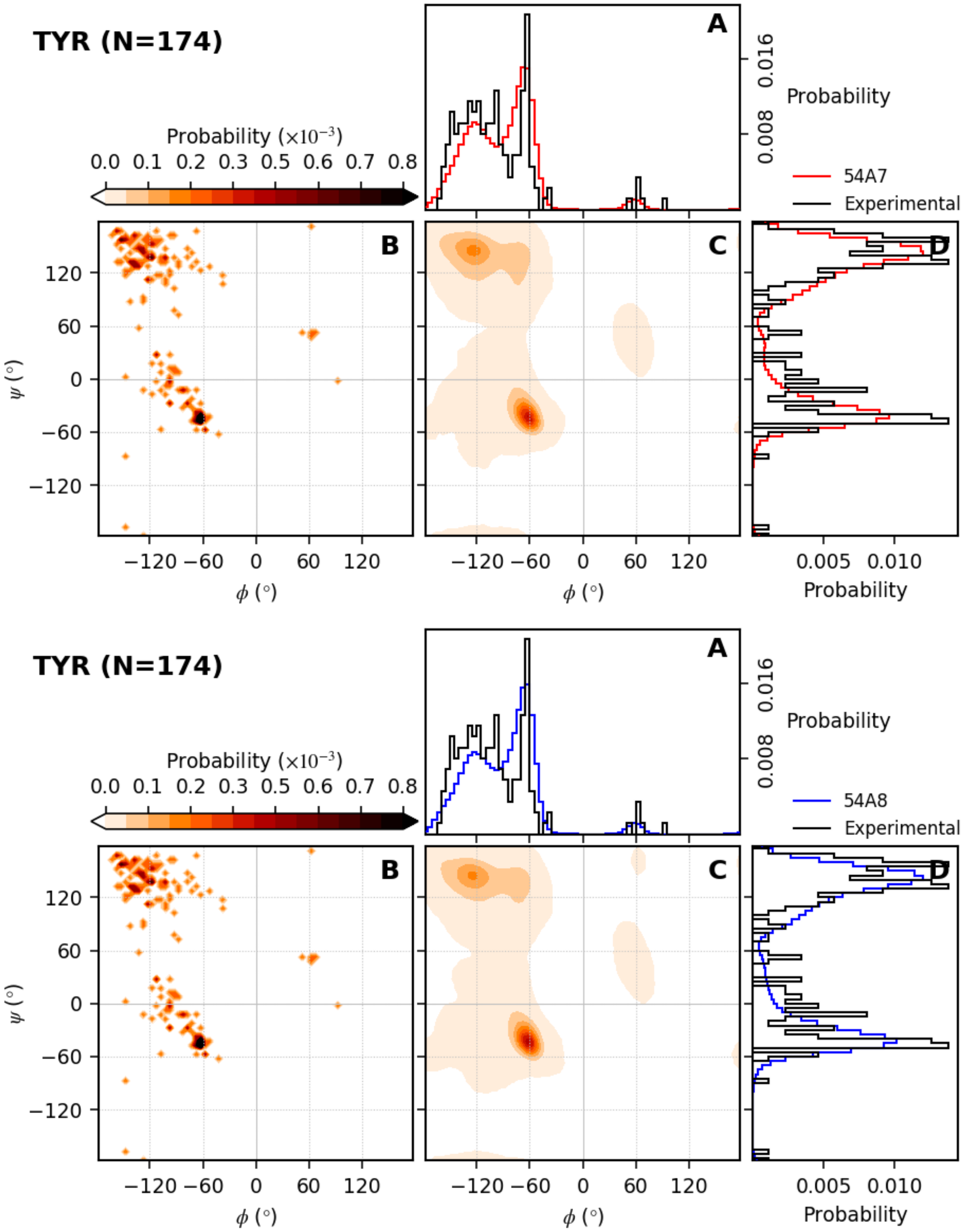

Figure S24. Distribution of tyrosine (TYR) backbone dihedral angles.

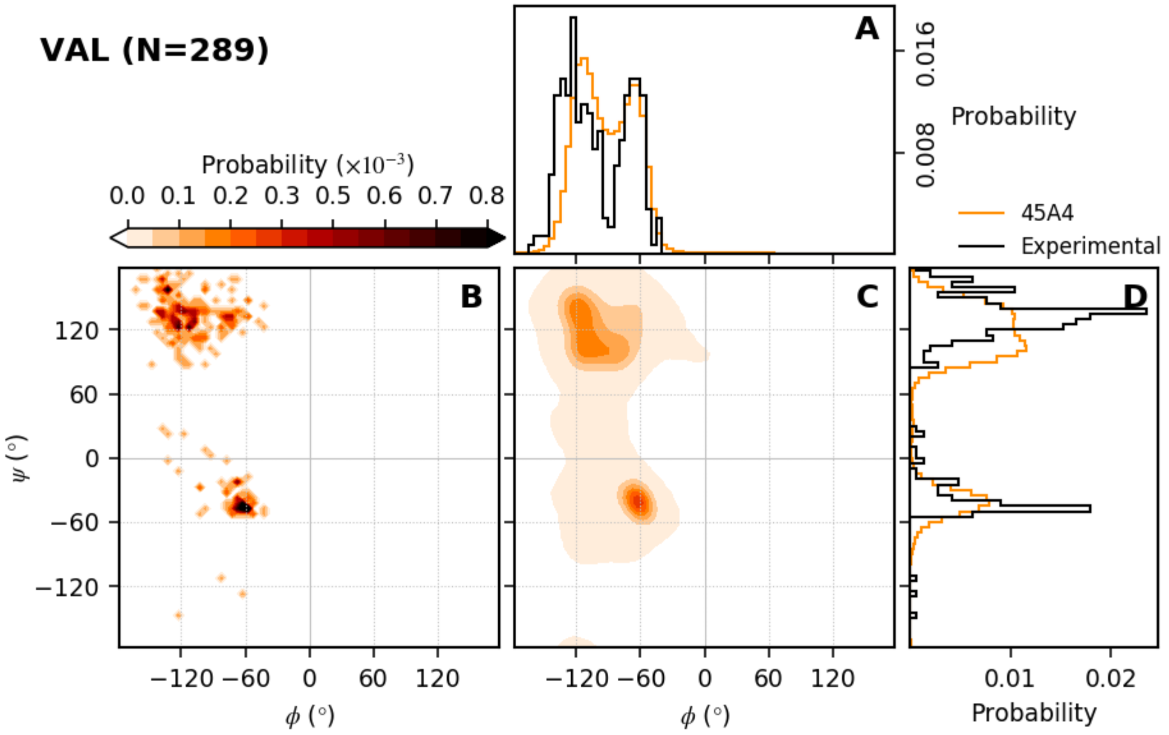
VAL (N=289)
Probability (×10⁻³)
0.0 0.1 0.2 0.3 0.5 0.6 0.7 0.8
A
B
C
D
0.016
0.008
Probability
45A4
Experimental
120
60
0
−60
−120
ψ (°)
−120 −60 0 60 120
ϕ (°)
0.01 0.02
Probability

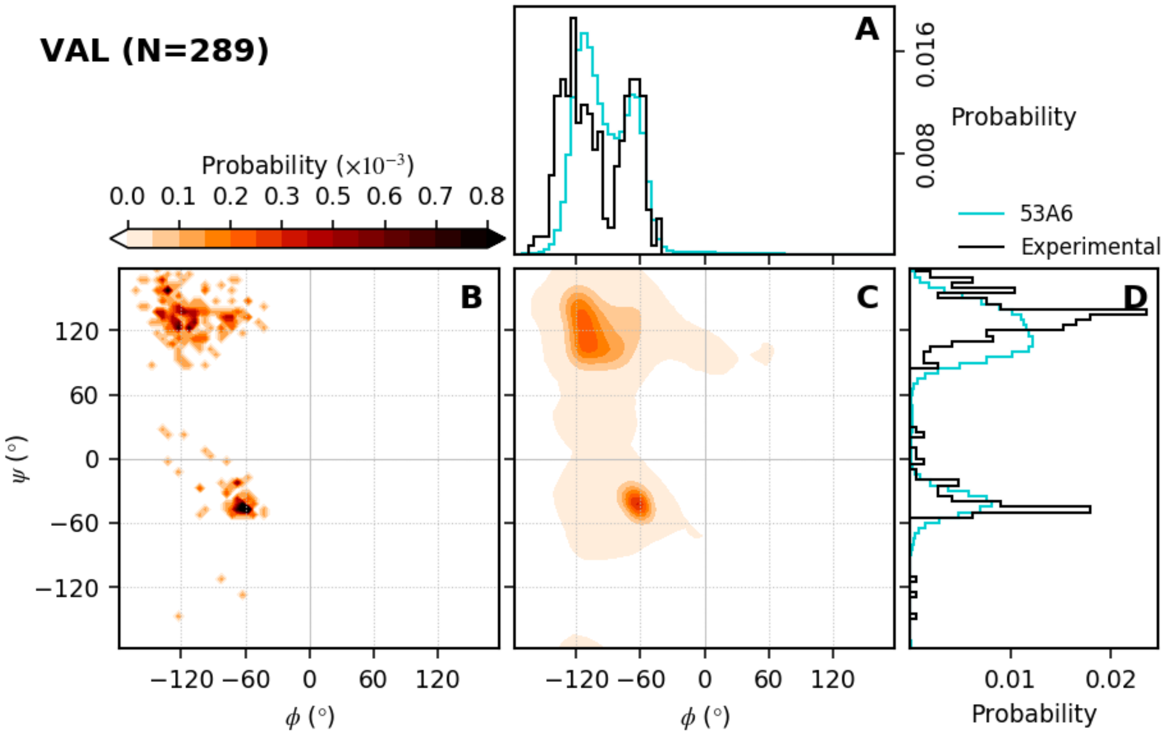
VAL (N=289)
Probability (×10⁻³)
0.0 0.1 0.2 0.3 0.5 0.6 0.7 0.8
A
B
C
D
0.016
0.008
Probability
53A6
Experimental
120
60
0
−60
−120
ψ (°)
−120 −60 0 60 120
ϕ (°)
0.01 0.02
Probability

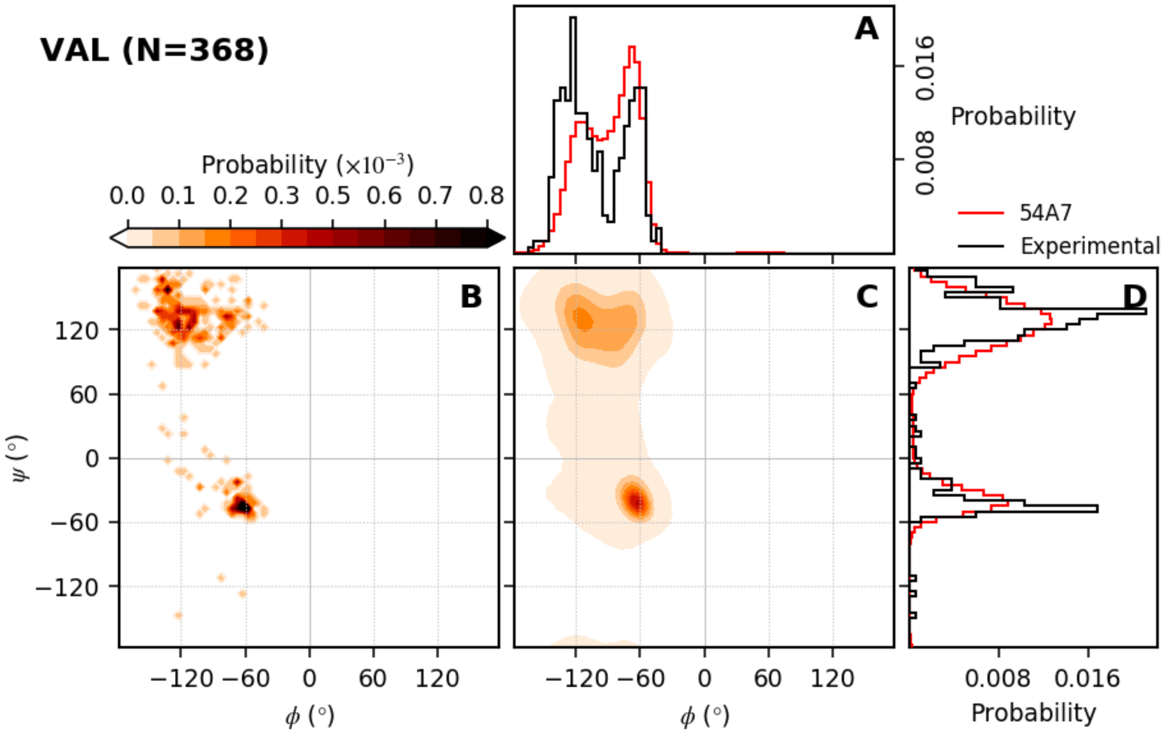

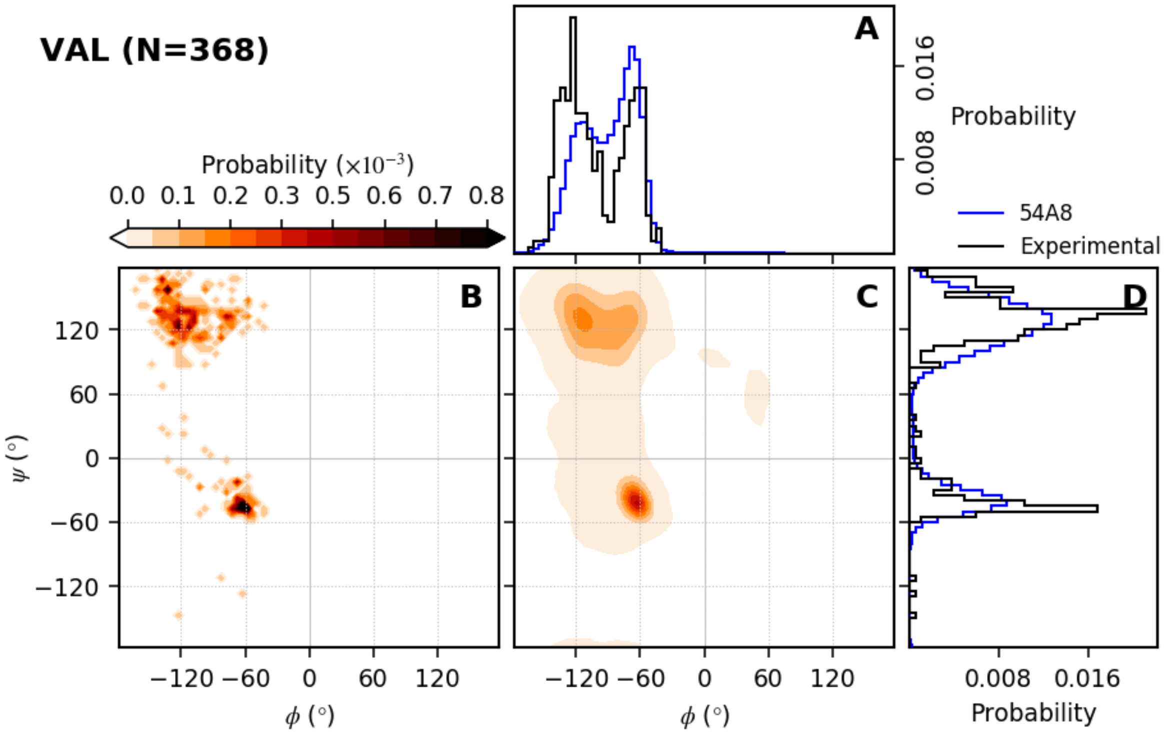

Figure S25. Distribution of valine (VAL) backbone dihedral angles.
